# Exposure to Per- and Polyfluoroalkyl Substances and the Risk of Sarcopenia: The Mediating Role of Serum Albumin

**DOI:** 10.3390/toxics14060478

**Published:** 2026-05-29

**Authors:** Mingkun Sun, Chu Chu, Kun Zhao, Zhengmin (Min) Qian, Mario Schootman, Stephen Edward McMillin, Jiaxiang Dong, Wenwen Bao, Muhammad Amjad, Haseeb Tufail Moryani, Yang Zhou, Yan Yang, Peipei Wang

**Affiliations:** 1 Joint International Research Laboratory of Environment and Health, Ministry of Education, Guangdong Provincial Engineering Technology Research Center of Environmental Pollution and Health Risk Assessment, Department of Occupational and Environmental Health, School of Public Health, Sun Yat-sen University, Guangzhou 510080, China; 2 Department of Epidemiology and Biostatistics, College for Public Health & Social Justice, Saint Louis University, Saint Louis, MO 63104, USA; 3 Department of Internal Medicine, College of Medicine, University of Arkansas for Medical Sciences, Springdale, AR 72762, USA; 4 School of Social Work, Saint Louis University, Saint Louis, MO 63103, USA; 5 Thornlea Secondary School, York Region District School Board, 8075 Bayview Avenue, Thornhill, ON L3T 4N4, Canada; 6 Engineering Research Center of Small Molecule Drugs, Ministry of Education, College of Public Health, Guangdong Pharmaceutical University, Guangzhou 510310, China; 7 Sleep Medicine Department, Sanya Central Hospital (The Third People’s Hospital of Hainan Province), Sanya 572029, China

**Keywords:** PFAS isomer, muscle loss, skeletal muscle, bio-electrical impedance analysis, liver dysfunction

## Abstract

Widespread exposure to per- and polyfluoroalkyl substances (PFAS) is a growing public health concern, but its link to muscle damage remains largely unexplored. As PFAS exposure is associated with liver dysfunction, which is an established risk factor for muscle damage, we examined their associations and potential mediating pathways. A total of 1261 participants were recruited from Guangdong province, China, from November 2018 to August 2019 and examined for muscle mass, strength, serum PFAS levels, and biomarkers of liver function. The key results demonstrated significant positive associations between serum PFAS exposure and sarcopenia risk. Specifically, a per ln ng/mL increase in linear perfluorooctane sulfonate (PFOS), branch PFOS, and perfluorooctanoic acid (PFOA) was associated with adjusted odds ratios of 2.32 (95% CI: 1.77 to 3.00), 2.18 (95% CI: 1.67 to 2.90) and 3.01 (95% CI: 1.96 to 4.70), respectively. Analysis of PFAS mixtures via the BKMR model revealed a linear dose–response relationship of sarcopenia, with PFOS and PFOA being the primary contributor. Importantly, mediation analyses showed that liver function biomarkers served as significant mediators of the PFAS–sarcopenia association. Notably, liver synthesis function markers (albumin and globin) mediated a substantial proportion of the association, ranging from 3.48% to 82.42%, whereas liver injury markers (aspartate aminotransferase and gamma-glutamyl transferase) accounted for only 1.54% to 15.44%. This study underscores the need to be aware of the increased risk of muscle damage associated with PFAS exposure, which may primarily operate through liver function abnormalities.

## 1. Introduction

Per- and polyfluoroalkyl substances (PFAS) constitute a class of synthetic fluorocarbons used extensively in industrial manufacturing and consumer products, leading to widespread distribution in environmental media (e.g., air, soil, water, and food) and human specimens (e.g., muscle, serum, and liver) [1,2,3,4]. PFAS exhibit a unique pharmacokinetic feature of strong binding affinity to proteins, with predominant accumulation occurring in the protein-rich organs. For instance, perfluorooctane sulfonate (PFOS) mainly accumulates in the liver and kidneys, followed by the blood and muscles, at concentrations of 6.3 to 13.6 ng/g, 2.2 to 6.4 ng/g, 1.5 to 5.1 ng/g, and 1.0 ng/g, respectively [5,6]. Emerging findings from both in vivo and in vitro experiments increasingly indicate that muscle appears to be a target organ of PFAS, across a wide spectrum from physiologically relevant (0.01 μM) to experimentally elevated (100 μM) concentrations [7,8,9,10,11]. However, epidemiological evidence on their association with muscle damage remains limited and inconsistent. One study from Sweden has linked PFAS exposure to reduced fat-free mass, a proxy for muscle mass, suggesting a potential association of higher PFAS concentrations with muscle composition impairment [12]. Conversely, another study using the 2011–2016 National Health and Nutrition Examination Survey (NHANES) found an inverse association between PFAS and sarcopenia [13]. Therefore, given the growing clinical and public health significance of sarcopenia, there remains a critical unmet need for further targeted studies in this field.

Sarcopenia, an age-related degenerative disorder defined by the progressive loss of muscle mass and function, significantly elevates the risk of multiple adverse health outcomes, such as falls, fractures, and mortality [14,15]. The worldwide prevalence of adult sarcopenia is 10–14%, with nearly one-third of patients experiencing falls or fractures [16,17,18]. The Global Burden of Disease showed that years lived with disability (YLD) caused by musculoskeletal disorders including sarcopenia continues to rise from 2010 to 2021, ranking as the fifth main cause of YLD in 2021 [19]. Given that sarcopenia poses a significant public health challenge worldwide, identifying its risk factors for primary prevention is critical. Previous evidence links sarcopenia to legacy pollutants like heavy metals and particulate matter [20,21], but the relationship and pathogenic mechanisms for PFAS, the emerging environmental pollutants, are still elusive.

Liver dysfunction is increasingly implicated in sarcopenia pathogenesis. Impaired liver function may systematically affect extrahepatic systems, including skeletal muscle [22]. Sarcopenia has been reported to be prevalent in 40–70% of cirrhosis patients, establishing itself as the foremost complication of chronic liver disease [23]. Furthermore, emerging genetic evidence, including a Mendelian randomization study, supports a causal link between liver disease and sarcopenia [24]. Notably, specific liver enzyme abnormalities may serve as predictive biomarkers for sarcopenia [25,26,27]. As the primary metabolic and detoxification organ, the liver is particularly vulnerable to PFAS accumulation and toxicity [5]. Existing epidemiological studies and animal experiments demonstrate that even low PFAS concentrations exhibit hepatotoxicity [28,29]. Recent studies based on data from the general U.S. population have reported positive associations of PFOA and PFOS exposure with the prevalence of metabolic dysfunction-associated fatty liver disease (MAFLD) and non-alcoholic fatty liver disease (NAFLD) [30,31]. However, the potential interaction between PFAS-induced liver dysfunction and sarcopenia development remains limited. Importantly, while PFAS may contribute to muscle injury through hepatic impairment, the multifactorial mechanism of sarcopenia must be further considered.

Therefore, a population-based study was conducted among Chinese adults to (1) examine associations between PFAS exposure and muscle damage; (2) assess the potential mediating roles of liver function; and (3) identify the predominant PFAS compound contributing to muscle damage and its key mediating pathway biomarker.

## 2. Methods

### 2.1. Study Population

From November 2018 to August 2019, we recruited 1524 residents from communities in Guangzhou, Guangdong Province, China, to investigate the potential effect of PFAS exposure and health outcomes in adults. The recruitment method for the study population has been described previously [32]. Briefly, three districts were randomly selected from the 11 districts in Guangzhou, namely Panyu, Yuexiu and Conghua, which represent the central business district, a suburban area, and an urban–rural integration area. One community per district was then randomly select. Finally, we recruited eligible individuals from approximately 100 randomly selected households: (1) resided in the community ≥ 2 years; (2) non-pregnant and non-lactating; (3) without severe chronic diseases. The participants provided three categories of data via face-to-face questionnaires, including basic demographic information, lifestyle factors, and health status [33]. Quality control was ensured by double-entering all data. Additionally, muscle mass and muscle strength were measured using bioelectrical impedance analysis (BIA) and a grip dynamometer, as these were non-invasive, convenient, and accurate methods, making them particularly suitable for community-based sarcopenia screening and diagnosis. Subsequently, 263 participants were excluded due to age < 18 years (*n* = 78), had a malignancy (*n* = 20), had missing data on the questionnaire (*n* = 134), or had missing muscle examinations (*n* = 31). The final analytic sample comprised 1261 adults, including 767 women and 494 men.

The study was conducted in accordance with the Declaration of Helsinki, and approved by the Ethics Committee of Sun Yat-Sen University on 15 June 2018 (approval number: L2018057). All participants provided written informed consent.

### 2.2. Liver Function Measurement

After an eight-hour fast, blood samples were collected, and we performed the following procedures: Serums were separated by centrifuging at 3000 rpm for 10 min at 4 °C. Then, we used the Roche Cobas c702 type autoanalyzer (Roche Ltd., Mannheim, Germany) to detect biomarkers of liver function. Serum albumin (ALB), globin (GLB), total protein (TP), alanine aminotransferase (ALT), aspartate aminotransferase (AST), and gamma-glutamyl transferase (GGT) were selected as biomarkers of liver function, which have been widely used as an index of liver health. Liver function tests are a cornerstone of clinical workflows for evaluating hepatic conditions. Specifically, GGT, AST, and ALT are markers of hepatocellular injury, with serum levels rising upon hepatocyte damage [25,26,27]. In contrast, TP, ALB, and GLB, which are synthesized by hepatocytes, serve as markers of hepatic synthetic function [34,35].

### 2.3. Serum PFAS Measurement

Serum 32 PFAS concentrations were quantitatively measured. Detailed analytic methods are described elsewhere [32]. Briefly, PFAS were extracted from 0.2 mL serum samples using solid-phase extraction (SPE) with Oasis-HLB cartridges (Corporation, Milford, MA, USA). Prior to extraction, the cartridges were conditioned with 2 mL methanol and 2 mL of 0.1 M formic acid. After sample loading, the cartridges were sequentially washed with 3 mL of 0.1 M formic acid, 6 mL 50% methanol/50% formic acid, and ammonium hydroxide to remove potential impurities. PFAS were then eluted with 2 mL 1% ammonium hydroxide in acetonitrile. The eluates were evaporated to near-dryness under nitrogen at 40 °C and subsequently reconstituted in 70 μL methanol and 30 μL of 20 mM ammonium formate. Finally, PFAS concentrations were quantified using ultra-performance liquid chromatography–tandem mass spectrometry (UPLC-MS/MS, Agilent Technologies Inc., Santa Clara, CA, USA). Quantification of PFAS was performed using an internal standards method with a nine-point calibration curve (0.05–100 ng/mL), blank controls and standard solution reagents. Detailed methods are provided in the Supplementary Materials. The limit of detection (LOD) was defined as the peak level of the detected sample with a signal-to-noise ratio (S/N) of 3. Those concentrations below the LOD were replaced by LOD/√2. Considering the concentration of samples below the detection limit, we eventually incorporated all PFAS at a detection rate greater than 85% in our analyses (Table S1). Concentrations of the linear and all branched isomers for the concentrations of perfluorooctane sulfonate (PFOS) and perfluorohexane sulfonate (PFHxS) were summed, and all the isomers were also included in the analyses to distinguish the specific effect.

### 2.4. Outcomes, Definition and Measurement

The muscle assessment of participants included both muscle mass and strength measurements, characterized by appendicular skeletal muscle mass index (ASMI) and maximum grip strength, respectively. We measured fat-free mass (FFM) as a proxy for muscle mass, using the bio-electric impedance method. Participants stood barefoot on a Tanita Body Composition Analyzer (Tanita Corporation, Tokyo, Japan) and held electrodes to complete the body composition test under medical supervision. At the same time, the maximum grip strength was measured using a grip dynamometer. We calculated the FFM Index (FFMI) and grip index (GripI) standardized by height squared (kg/m^2^). ASMI was calculated using the FFMI formula, which has been verified and developed in the Asian population [36]. The Asian Working Group for Sarcopenia (2025) consensus recommends that sarcopenia be identified when both low muscle mass and low muscle strength are present in community screening [37]. Sarcopenia in people over 65 years was defined as ASMI < 7.0 kg/m^2^ and grip < 28 kg in men and ASMI < 5.7 kg/m^2^ and grip < 18 kg in women, which is validated in the Asian population. For the youngest people, sarcopenia was defined as ASMI < 7.6 kg/m^2^ and grip < 34 kg in men and ASMI < 5.7 kg/m^2^ and grip < 20 kg in women [37].

### 2.5. Covariates

Covariates were selected based on previous research on the association of PFAS with muscle. Data was obtained from the questionnaire, including: (1) sociodemographic characteristics: sex (men or women), age (years), education (<high school or ≥high school), family income per year (≤30,000 CNY, 30,000–100,000 CNY, or >100,000 CNY), body mass index (BMI, kg/m^2^); (2) lifestyle factors: smoking (yes or no), alcohol consumption (yes or no), exercise (yes or no). Participants who smoked ≥1 cigarette a day for at least one year were defined as “smoking”; drinking alcoholic beverages every week was defined as “alcohol consumption”; exercising at least three days per week for ≥20 min a day was considered “exercise” [32].

### 2.6. Statistical Analysis

#### 2.6.1. Descriptive Analysis

Q-Q plots and the Shapiro–Wilk test were applied to check the normality of continuous variables. Median (quartile 1, Q1 and quartile 3, Q3) was used for presenting variables with skewed distribution, with mean (standard deviations, SDs) for variables with normal distribution, and numbers (percentages) for categorical variables. To compare continuous variables between the two groups, the analysis used either the Student *t*-test or the Wilcoxon rank-sum test, depending on the characteristics of the variable. The chi-square test was employed to assess the significance of differences in proportions across categories. Similarly, PFAS concentrations, biomarkers of liver function and muscle parameters were compared among three participant groups [districts (Panyu, Yuexiu, Conghua)] using the Kruskal–Wallis rank-sum test or one-way ANOVA.

#### 2.6.2. Single PFAS Model Analysis

The PFAS levels were transformed using the natural logarithm before analysis. A generalized linear model (GLM) was employed to analyze the associations between individual PFAS compounds and outcomes. Specifically, a GLM with a binomial distribution and a logit link was applied for sarcopenia, and a GLM with a Gaussian distribution and identity link was used for the continuous outcomes of ASMI and GripI. All models were adjusted for age, sex, education, alcohol drinking, smoking, family income, exercise, BMI, and district. The subgroup analyses were also stratified by age (<60 years and ≥60 years) and sex (men and women).

#### 2.6.3. PFAS Mixture Model Analysis

The Bayesian kernel machine regression (BKMR) was applied to analyze the effect of PFAS mixtures on sarcopenia. Considering that PFAS compounds are highly correlated, this approach, regressing an exposure–response function iteratively by a Gaussian kernel function, can explore the interactions between the PFAS mixture and sarcopenia. Therefore, this study set a BKMR model with 10,000 iterations by a Markov Chain Monte Carlo (MCMC) algorithm and used a hierarchical variable selection approach by dividing 16 PFAS into three groups, namely isomers of PFOS, other perfluoroalkane sulfonic acids (PFSAs), and perfluorocarboxylic acids (PFCAs). The group posterior inclusion probabilities (group PIPs and conditional PIPs) were calculated to identify the main effect between PFAS compounds and sarcopenia using a threshold value of 0.5. The health effect of an individual PFAS was estimated as it changed from the 25th percentile to the 75th percentile while keeping the other PFAS compounds at a fixed level.

#### 2.6.4. Mediation Analysis

The “mediation” package of R (4.3.3 version) was used to explore whether biomarkers of liver health are potential mediators of the association between PFAS exposure and sarcopenia, including estimating the direct effect (DE), indirect effect (IE), and total effect (TE). The DE represents the effect of PFAS on sarcopenia in the absence of mediation, the IE estimates the effect of PFAS on sarcopenia through these mediators, and the TE reflects both the direct and indirect pathways.

#### 2.6.5. Sensitivity Analysis

The robustness of the main results was evaluated by conducting several sensitivity analyses. (1) Smokers (*n* = 270) and alcohol drinkers (*n* = 206) were excluded from the analysis, since some unhealthy lifestyles were linked to lower muscle mass [38,39]. (2) In addition, participants with specific diseases recognized as causes of muscle loss were also excluded [39]. Participants with diabetes (*n* = 118), identified through a questionnaire survey, were excluded. Participants with osteoporosis (*n* = 217), defined as a bone mineral density T-score < −2.5, were also excluded [40]. Participants with malnutrition (*n* = 64), defined as BMI < 18.5 kg/m^2^, were also excluded [41]. (3) To address potential bias from constant-value substitution (LOD/√2) for PFAS concentrations below the LOD, concentrations below the LOQ were excluded from the primary analyses. (4) The interpretation of liver function as a mediator in the PFAS–sarcopenia association is potentially confounded by several diseases that affect liver enzyme levels, namely chronic kidney disease (CKD), malnutrition, and inflammation. Therefore, participants with these conditions were excluded from the mediation analysis. CKD was defined as an estimated glomerular filtration rate (eGFR) < 60 mL/minute/1.73 m^2^, with eGFR calculated using the CKD-EPI 2009 formulas [42]. Inflammation was defined as a neutrophil-to-lymphocyte ratio (NLR) > 3 [43]. (5) To minimize potential interference from medication-induced liver abnormalities or sarcopenia (e.g., due to statins or other cardiovascular drugs), subjects with coronary heart disease (CHD), stroke, or dyslipidemia were excluded [44].

Statistical significance was defined as *p* < 0.05, using a 2-tailed test. All statistical analyses were performed using R 4.3.3.

## 3. Results

### 3.1. Population Characteristics

Table 1 shows the characteristics of 1261 eligible participants in the study. The overall prevalence of sarcopenia was 11.42%, with an average age of 62.76 ± 13.02 years among affected individuals. The prevalence of sarcopenia was 14.98% in men and 9.13% in women. Compared to those without sarcopenia, participants screened for sarcopenia had less muscle tissue (6.95 vs. 5.67 kg/m^2^, *p* < 0.001) and lower grip strength (28.65 vs. 21.01 kg, *p* < 0.001). Participants from different districts showed significant differences in muscle parameters, liver enzyme levels, and PFAS concentrations (*p* < 0.05, Table S2).

### 3.2. Serum PFAS Concentrations

Table 2 shows the distributions of serum PFAS concentrations. Among the 16 PFAS with a detection proportion of >85%, the median concentration of serum linear PFOS was the highest (10.83 ng/mL), followed by the PFOA (8.93 ng/mL) and branched PFOS (3.66 ng/mL). PFAS concentrations were generally higher among sarcopenia than non-sarcopenia participants. The correlations between PFAS ranged from −0.02 to 0.94 (Figure S1).

### 3.3. Associations Between Individual PFAS Exposure and Sarcopenia

Table 3 presents the results from covariate-adjusted logistic regression models examining associations between PFAS and sarcopenia. Overall, higher serum PFAS was associated with increased odds of sarcopenia, with each ln-unit (ng/mL) increase in PFOS elevating the risk by 1.24 to 3.01-fold. Specifically, linear PFOS (OR = 2.32, 95% CI: 1.77 to 3.09, *p* < 0.001) showed a stronger association than its branched isomers, which included 1m-PFOS (OR = 1.50, 95% CI: 1.15 to 1.97, *p* = 0.003), iso-PFOS (OR = 1.79, 95% CI: 1.39 to 2.19, *p* < 0.001), 3 + 4 + 5m-PFOS (OR = 1.71, 95% CI: 1.39 to 2.19, *p* < 0.001), and ∑m2-PFOS (OR = 1.45, 95% CI: 1.12 to 1.86, *p* = 0.004).

Specifically, all PFAS compounds were negatively associated with ASMI, such as each ln-unit (ng/mL) increase in total PFOS, and PFOA were associated with estimated changes in ASMI of −0.78 (95% CI: −0.84, −0.73; *p* < 0.001) kg/m^2^ and −0.58 (95% CI: −0.65, −0.50; *p* < 0.001) kg/m^2^, respectively. Among PFOS isomers, linear PFOS showed a more pronounced reduction in ASMI (−0.70 kg/m^2^ per ln-unit increase), whereas the effect of branched isomers was substantially weaker, ranging from −0.10 (for 3 + 4 + 5m-PFOS) to −0.67 (for iso-PFOS). In addition, 3 + 4 + 5m-PFOS was also negatively associated with grip strength (β = −0.29; 95% CI: −0.36, −0.22; *p* < 0.001), whereas linear PFOS showed no significant association.

### 3.4. Association Between PFAS Mixture Exposure and Sarcopenia

**Figure 1:**
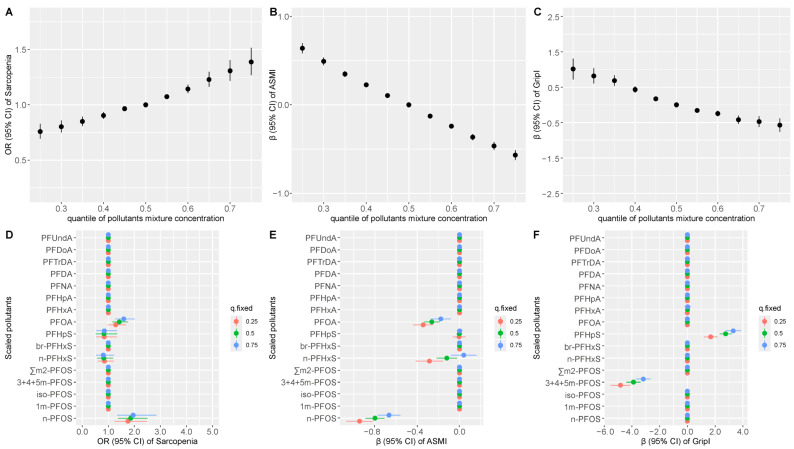
The overall effect of PFAS mixture and single exposure–response of PFAS with sarcopenia in Bayesian kernel machine regression (BKMR) model. The model was adjusted for age, sex, education, alcohol drinking, smoking, family income, exercise, BMI, and district. (**A**): The overall effect of PFAS mixture on the odds ratio of sarcopenia. (**B**): The overall effect of PFAS mixture on the β of ASMI. (**C**): The overall effect of PFAS mixture on the β of GripI. (**D**): Single PFAS exposure–response on the odds ratio of sarcopenia when all other PFAS were fixed at the 25th, 50th and 75th percentiles. (**E**): Single PFAS exposure–response on the β of ASMI when all other PFAS were fixed at the 25th, 50th and 75th percentiles. (**F**): Single PFAS exposure–response on the β of GripI when all other PFAS were fixed at the 25th, 50th and 75th percentile. Abbreviations: ASMI, appendicular skeletal muscle mass index; GripI, grip index; n-PFOS, linear PFOS; Br-PFOS, branched PFOS.

BKMR models showed that the PFAS mixture had positive joint associations with sarcopenia risk and negative joint associations with ASMI and GripI (Figure 1). For example, fixing the PFAS mixture concentration at the 75th percentile of the exposure distribution was associated with a higher risk of sarcopenia (OR: 1.39; 95% CI: 1.27, 1.52; Table S4) compared with the 50th percentile. The contribution of individual PFAS components to the joint associations with sarcopenia was further characterized. Linear PFOS and PFOAs were identified as the dominant contributors to the joint association with sarcopenia. When other PFAS were fixed at the 75th percentile, n-PFOS was associated with 1.95-fold (95% CI: 1.34, 2.85) higher odds of sarcopenia, and PFOA with 1.59-fold (95% CI: 1.26, 2.02) higher odds (Table S5). For ASMI, linear PFOS, PFOA, and linear PFHxS were the major contributors, whereas for grip strength, 3 + 4 + 5m-PFOS contributed the most, with posterior inclusion probabilities (PIPs) approaching 1 (Table S3).

### 3.5. Mediating Pathways Between PFAS Exposure and Sarcopenia

Given the significant associations of PFAS with biomarkers of liver function and the considerable association of biomarkers and sarcopenia (Tables S3 and S4), the mediating roles of the biomarkers in the PFAS–sarcopenia association were further examined. The mediation analysis revealed that liver function significantly mediated the associations between PFAS and sarcopenia. For biomarkers of liver injury, the proportions mediated by AST and GGT between PFAS exposure and sarcopenia ranged from 1.55% to 15.44% (Table 4 and Tables S8). Moreover, liver synthesis dysfunction explained a substantial proportion of the association between PFAS and sarcopenia. For instance, the mediated proportions for ALB were 81.74% (95% CI: 29.76, 247.31) for 1m-PFOS, 46.43% (95% CI: 14.42, 108.60) for iso-PFOS, 8.31% (95% CI: 2.38, 18.50) for 3 + 4 + 5m-PFOS, 82.42% (95% CI: 36.07, 249.96) for ∑m2-PFOS, and 22.31% (95% CI: 6.95, 46.18) for PFOA. Similarly, the level of GLB also mediated 3.48 to 16.14% of the associations between leaner PFOS and branched PFOS with sarcopenia. After excluding potential confounding factors, the results remained consistent with the main findings regarding the mediating effect of liver function (Tables S11 and S12). Furthermore, these biomarkers of liver function also accounted for substantial proportions of the associations in sarcopenia parameters (Tables S9 and S10).

### 3.6. Sensitivity Analysis Results Between PFAS Exposure and Sarcopenia

In subgroup analyses, women were more likely to be affected by PFAS exposure (Table S13). Despite a trend towards a stronger effect of PFAS in the elderly population, no statistically significant difference was found between the elderly and younger groups (Table S14). Results from the sensitivity analyses suggest that the main results were robust (Tables S15–S22).

## 4. Discussion

This study observed associations between PFAS exposure and muscle status in a community-based adult population. Overall PFAS mixture exposure was associated with higher odds of sarcopenia, with PFOS and PFOA appearing to contribute substantially to the mixture effect. In addition, PFAS were also associated with lower ASMI and reduced GripI, indicating that PFAS exposure may be linked to adverse changes in both muscle quantity and muscle function. Mediation analysis further suggested that selected biomarker of liver function, particularly ALB, may be implicated in these associations. These findings offer a potential new perspective on the link between PFAS exposure and muscle health, specifically by highlighting liver function changes as a possible intermediary factor.

To date, epidemiological evidence has been limited regarding the damaging effects of PFAS on muscle health. A recent study has evaluated the relationship between PFAS and FFM among 502 participants with an average age of 50 years in Sweden, finding a negative association between PFAS exposure and FFM, for example, PFHxS exposure in males (β: −0.22, 95% CI: −0.42, −0.01) and PFDA exposure in females (β: −0.31, 95% CI: −0.61, −0.01) [12]. These findings are consistent with our results. However, Tao et al. analyzed 2106 participants from NHANES and found a significant negative association between PFOS and sarcopenia (OR = 0.77, 95% CI: 0.62, 0.95), whereas no such association was found for PFOA, PFNA, or PFHxS [13]. Against this backdrop of inconsistent evidence, the present study adds further epidemiological clues suggesting that higher PFAS exposure may be associated with increased odds of sarcopenia, potentially partly through lower ASMI.

Evaluating the combined effects of PFAS mixtures on muscle health better reflects real-world exposure scenarios. The observed mixture results suggested potential associations between combined PFAS exposure and poorer muscle health. These findings stand in contrast to previous evidence. For instance, a cross-sectional study of 1067 U.S. adolescents aged 12–18 years found that a four-PFAS mixture (PFOS, PFHxS, PFOA, and PFNA) was positively associated with FFMI [45]. This discrepancy may stem from adolescence being a period of rapid muscle development, where growth peaks could mask the adverse effects of PFAS on muscle health that may become more detectable in populations with slower muscle turnover or older age groups [46,47,48]. Additionally, no association was observed between the PFAS mixture (PFHxS, PFOA, PFOS, PFNA) and sarcopenia in a study of 2106 adults using WQS analysis [13]. The inconsistency across results may further arise from differences in study design, population characteristics, and exposure concentrations.

Furthermore, this study explores the potential isomer-specific associations between PFAS exposure and muscle health parameters. Linear PFAS isomers demonstrated the strongest negative correlations with sarcopenia prevalence and ASMI, whereas branched isomers showed preferential association with reduced grip strength. The emerging evidence suggests that different PFAS isomers exhibit distinct toxicological profiles. For example, the Isomers of C8 Health Project demonstrated that linear PFOS displayed a stronger association with overweight/obesity than branched PFOS (OR: 1.45, 95% CI: 1.06, 1.99 vs. 1.33, 95% CI: 1.00, 1.77) [49]. Similarly, our prior work identified branched PFOS as significantly associated with reduced bone mineral density (β = −0.12, 95% CI: −0.20, −0.04) [40]. These isomer-specific effects likely stem from their distinct pharmacokinetic properties, including tissue-specific accumulation patterns (linear PFOS dominates in muscle tissue [50]), receptor binding affinities, and transcriptional activities [51,52]. Mechanistically, pronounced effects of linear isomers on muscle mass directly correlate with their tissue-specific bioaccumulation. Conversely, the grip strength reduction associated with branched isomers may reflect compromised bone-muscle crosstalk, as skeletal integrity provides essential biomechanical support for force generation [53]. Further mechanistic studies are needed to clarify these differential effects fully.

Although epidemiological evidence linking PFAS to grip strength is limited, recent toxicological studies suggest that PFAS may impair muscle function. Male C57BL/6 mice exposed to PFOS exhibited reduced motor activity and grip strength, whereas PFOA exposure showed a non-significant decreasing trend in grip strength [10]. Similarly, another experiment found that PFOS impaired motor function in adult zebrafish at doses >0.3 mg/L [11]. Transcriptomic analysis of muscle cells from zebrafish larvae exposed to 16 μM PFOS identified 95 differentially expressed genes, resulting in impaired metabolism and overall development [54]. Together, these toxicological findings provide biological support for the observed associations and, given the public health relevance of sarcopenia, highlight the need for further longitudinal and mechanistic studies on PFAS exposure and muscle function.

A key finding of this study is that biomarkers of liver injury partially mediated the association between PFAS exposure and sarcopenia, accounting for 1.54% to 15.44% the effect. Specifically, AST and GGT appeared to be the main contributors. These enzymes are predominantly expressed in hepatocytes, and elevated serum levels are considered clinical indicators of liver damage [55]. PFAS exposure has been associated with liver damage and hepatic dysfunction, consistent with epidemiological links between PFAS levels and abnormal liver biomarkers [56,57,58,59,60]. For instance, a study from NHANES demonstrated that PFAS exposure was positively associated with elevated ALT and AST levels [61]. Abnormal liver function can usually predict sarcopenia. On the one hand, hyperammonemia resulting from impaired hepatic ammonia metabolism is a well-established key mediator within the liver–intestinal axis and contributes to sarcopenia pathogenesis by inducing mitochondrial dysfunction and upregulating myostatin expression [62,63]. On the other hand, hepatic dysfunction compromises gastrointestinal function, leading to insufficient intake and absorption of macronutrients, thereby directly impairing the synthesis of skeletal muscle proteins [64]. However, another study reports that alkaline phosphatase mediated a negative association between PFOS and sarcopenia (2.87%, 95% CI: 0.07%, 8.00%) [13]. This discrepancy may be explained by two factors. Firstly, the median serum PFOS concentration among the 2106 US participants was 5.10 ng/mL, substantially lower than that in the present study. Secondly, demographic differences between study populations and the choice of covariates may have influenced the findings.

Moreover, results of the mediation analysis provided hints that the abnormal liver synthesis function served as a primary mediator in the association between PFAS and sarcopenia. ALB and GLB are specifically produced by hepatocytes in the liver. Hence, their circulating levels are routinely measured as biomarkers of hepatic protein-synthesizing capacity and contribute to the diagnostic process for liver disorders [65]. Furthermore, the liver can respond to exogenous toxic insults by initiating hepatocyte regeneration and compensatorily enhancing hepatic synthesis [66]. Consistent with the present findings, animal evidence from male mice exposed to 0–10 mg/g PFOA and its alternatives demonstrated apparent dose-dependent hepatic toxicity. The observed alterations included hepatocyte hypertrophy, elevated serum AST, ALT and ALB concentrations, as well as the activation of fatty acid metabolism and the PPAR signaling pathway [67]. Although serum albumin can be influenced by various non-hepatic factors, potential confounding from chronic kidney disease and malnutrition were largely minimized in the present study by excluding individuals with these conditions through targeted sensitivity analysis. Consequently, our findings may retain some relevance in the context of public health, particularly with regard to the identification of mediating factors potentially linking PFAS exposure to sarcopenia. However, the mechanisms by which serum ALB and GLB levels contribute to this association remain unclear, and further studies could help clarify these effects and explore the potential mechanisms involved.

The other mechanisms underlying the increased risk of sarcopenia associated with PFAS exposure remain poorly understood. Oxidative stress may be another critical factor contributing to the associations between PFAS and muscle damage. Toxicological studies have shown that human muscle cells exposed for 24 h to short-chain PFAS (1 nM to 1 μM) disturbed the balance of antioxidant defense, leading to cytotoxicity [68]. Moreover, toxicological evidence identifies inflammation as a fundamental mechanism in PFAS-related pathologies, with zebrafish studies showing PFOS exposure induces muscle damage and functional impairment accompanied by high levels of inflammation [69]. Similarly, experiments using zebrafish have shown that exposure to PFOS induces oxidative stress and lipid peroxidation; these pathological processes, in turn, result in karyopyknosis and the disintegration of striated muscle cells [70]. It is widely recognized that mitochondrial dysfunction acts as a pivotal driver in sarcopenia pathogenesis, and this notion is supported by a prior study of muscle biopsies from 119 men of diverse ethnic backgrounds [71,72,73]. Specifically, individuals with sarcopenia consistently exhibited reduced expression of mitochondrial function-linked genes in their skeletal muscle [71]. Extending this line of inquiry into toxicological contexts, further research has found that exposing L4-stage C. elegans to 25 nM or 50 nM PFAS for 7 days induces mitochondrial autophagy dysfunction and impairs motor behavior [74].

This study has some major strengths. The use of a community-based population sample may improve the generalizability of findings regarding the association between PFAS exposure and sarcopenia. Secondly, sarcopenia was assessed using both measures of muscle mass and muscle strength. This dual-parameter approach reduces the risk of misdiagnosis, as defining the condition solely through either muscle mass or grip strength can overestimate its severity. In addition, this study suggested the potential role of liver-function-related biomarkers in the association between PFAS exposure and muscle health, which may help inform future mechanistic and longitudinal studies.

This study also has several limitations. Firstly, cross-sectional studies are unable to make causal inferences. However, the half-life of PFAS may reflect the time sequence of exposure–outcome to some extent. Secondly, biomarkers of liver function are dynamic processes, and measuring levels of the biomarkers at only one time point may introduce assessment bias. In particular, serum ALB is not a liver-specific biomarker and can be influenced by multiple non-hepatic factors, including nutritional status, renal function, inflammation, and systemic diseases. Therefore, the interpretation of findings should be cautious. Nevertheless, sensitivity analyses excluding participants with chronic kidney disease, malnutrition and inflammation were conducted to partially reduce the potential influence of these confounding factors. Thirdly, although several potential confounders related to muscle health were adjusted in our study, we were unable to account for all possible confounders, such as dietary habits, nutritional supplements, and medicine. To partially address the potential influence, participants with these special diseases were excluded in sensitivity analyses, and the results remained generally stable. Finally, this study did not assess other environmental pollution [75,76]. Future prospective studies are needed to systematically evaluate these individual and combined effects of pollutants on muscle health deterioration.

## 5. Conclusions

This study suggests that higher PFAS exposure, particularly PFOA and PFOS, may be associated with poorer muscle health and increased odds of sarcopenia. Liver dysfunction, particularly disrupted hepatic synthetic function, may serve as a mediator in the association between PFAS exposure and muscle damage. The observed links among PFAS exposure, liver function, and sarcopenia highlight the need for future longitudinal and mechanistic studies, as well as public health efforts to reduce population-level PFAS exposure and protect muscle health in environmentally exposed populations.

## Figures and Tables

**Table 1:** Descriptive statistics of study participants (N = 1261).

Variables	Total (*n* = 1261)	Non-Sarcopenia (*n* = 1117)	Sarcopenia (*n* = 144)	*p*-Value
Demographic characteristics				
Age, Mean ± SD, years	54.94 ± 15.36	53.93 ± 15.35	62.76 ± 13.02	<0.001
BMI, Mean ± SD, kg/m^2^	23.81 ± 3.64	24.09 ± 3.59	21.64 ± 3.30	<0.001
Women, No. (%)	767 (60.82)	697 (62.40)	70 (48.61)	0.001
Education ≥ high school, No. (%)	735 (58.29)	670 (59.98)	65 (45.14)	<0.001
Family Income, No. (%), CNY/year				0.306
<30,000	58 (4.60)	50 (4.48)	8 (5.56)	
30,000–100,000	515 (40.84)	449 (40.20)	66 (45.83)	
>100,000	688 (54.56)	618 (55.33)	70 (48.61)	
District, No. (%)				<0.0001
Conghua	233 (18.48%)	221 (19.79%)	12 (8.33%)	
Panyu	719 (57.02%)	612 (54.79%)	107 (74.31%)	
Yuexiu	309 (24.50%)	284 (25.43%)	25 (17.36%)	
Alcohol drinking, No. (%)	206 (16.34)	181 (16.20)	25 (17.36)	0.724
Smoking, No. (%)	270 (21.41)	233 (20.86)	37 (25.69)	0.183
Exercise, No. (%)	867 (68.75)	753 (67.41)	114 (79.17)	0.004
Parameters of muscle assessment				
ASMI, Mean ± SD, kg/m^2^	6.81 ± 1.36	6.95 ± 1.34	5.67 ± 0.96	<0.001
Grip, Mean ± SD, kg	27.78 ± 10.01	28.65 ± 10.02	21.01 ± 6.84	<0.001
Biomarkers of liver function				
ALB, Mean ± SD, g/L	48.34 ± 4.49	48.08 ± 4.50	50.42 ± 3.82	<0.001
GLB, Mean ± SD, g/L	29.80 ± 4.30	29.55 ± 4.13	31.80 ± 5.01	<0.001
TP, Mean ± SD, g/L	77.73 ± 4.03	77.78 ± 4.00	77.41 ± 4.22	0.330
ALT, Mean ± SD, U/L	21.50 ± 15.93	21.68 ± 16.33	20.08 ± 12.40	0.259
AST, Mean ± SD, U/L	22.32 ± 19.39	21.73 ± 8.97	26.92 ± 51.59	0.230
GGT, Mean ± SD, U/L	32.83 ± 53.65	31.82 ± 46.39	40.67 ± 92.19	0.062

Abbreviations: BMI, body mass index; CNY, Chinese Yuan; ASMI, appendicular skeletal muscle mass index; ALB, albumin; GLB, globin; TP, total protein; ALT, alanine aminotransferase; AST, aspartate aminotransferase; GGT, gamma-glutamyl transferase; SD, standard deviation.

**Table 2:** Serum concentrations (ng/mL) of PFAS measured among study participants (N = 1261).

PFAS (ng/mL)	Total (*n* = 1261)	Non-Sarcopenia (*n* = 1117)	Sarcopenia (*n* = 144)	*p*-Value
	Median (Q1, Q3)			
Total PFOS	14.66 (8.30, 25.39)	13.76 (7.91, 22.91)	24.86 (15.75, 39.42)	<0.001
n-PFOS	10.83 (6.21, 18.77)	10.23 (5.87, 17.43)	18.87 (11.40, 30.33)	<0.001
Br-PFOS	3.66 (1.79, 6.16)	3.37 (1.64, 5.74)	6.11 (3.71, 9.35)	<0.001
1m-PFOS	0.49 (0.24, 0.89)	0.47 (0.22, 0.83)	0.74 (0.45, 1.14)	<0.001
iso-PFOS	0.74 (0.42, 1.40)	0.70 (0.40, 1.30)	1.29 (0.73, 2.19)	<0.001
3 + 4 + 5m-PFOS ^a^	2.35 (0.96, 4.11)	2.11 (0.83, 3.78)	4.00 (2.37, 6.37)	<0.001
∑m2-PFOS ^a^	0.04 (0.02, 0.07)	0.04 (0.02, 0.06)	0.06 (0.03, 0.09)	<0.001
PFHpS	0.33 (0.17, 0.51)	0.31 (0.16, 0.49)	0.44 (0.30, 0.64)	<0.001
Total PFHxS	0.89 (0.49, 1.39)	0.84 (0.46, 1.37)	1.10 (0.76, 1.46)	<0.001
n-PFHxS	0.87 (0.47, 1.37)	0.82 (0.44, 1.36)	1.06 (0.73, 1.42)	<0.001
Br-PFHxS	0.02 (0.01, 0.03)	0.02 (0.01, 0.03)	0.02 (0.01, 0.03)	0.376
PFOA	8.93 (5.37, 13.53)	8.60 (5.09, 12.92)	12.89 (8.73, 16.28)	<0.001
PFHpA	0.03 (0.02, 0.06)	0.03 (0.02, 0.06)	0.04 (0.02, 0.08)	<0.001
PFHxA	0.04 (0.01, 0.07)	0.04 (0.01, 0.08)	0.04 (0.01, 0.07)	0.620
PFNA	1.11 (0.69, 1.65)	1.05 (0.67, 1.60)	1.51 (1.10, 2.30)	<0.001
PFDA	0.84 (0.52, 1.40)	0.80 (0.49, 1.30)	1.38 (0.87, 1.87)	<0.001
PFUnDA	0.73 (0.44, 1.14)	0.68 (0.42, 1.09)	1.02 (0.71, 1.50)	<0.001
PFDoDA	0.06 (0.03, 0.10)	0.06 (0.03, 0.09)	0.09 (0.06, 0.14)	<0.001
PFTrDA	0.27 (0.16, 0.44)	0.26 (0.15, 0.42)	0.39 (0.24, 0.56)	<0.001

Abbreviations of PFAS, see Table S1. ^a^ Isomers of PFOS; m: the perfluoromethyl branch; the preceding number: the carbon position.

**Table 3:** Effect for the association between serum PFAS and sarcopenia (N = 1261).

PFAS ^a^ (ng/mL)	Sarcopenia		ASMI		GripI	
	OR (95% CI)	*p*	β (95% CI)	*p*	β (95% CI)	*p*
Total PFOS	**2.49 (1.86, 3.36)**	**<0.001**	**−0.78 (−0.84, −0.73)**	**<0.001**	−0.08 (−0.30, 0.15)	0.490
n-PFOS	**2.32 (1.77, 3.09)**	**<0.001**	**−0.70 (−0.75, −0.65)**	**<0.001**	0.14 (−0.06, 0.35)	0.171
Br-PFOS	**2.18 (1.67, 2.90)**	**<0.001**	**−0.40 (−0.45, −0.35)**	**<0.001**	**−0.51 (−0.68, −0.35)**	**<0.001**
1m-PFOS	**1.50 (1.15, 1.97)**	**0.003**	**−0.55 (−0.60, −0.50)**	**<0.001**	0.21 (0.01, 0.40)	0.036
iso-PFOS	**1.79 (1.39, 2.33)**	**<0.001**	**−0.67 (−0.72, −0.61)**	**<0.001**	0.19 (−0.02, 0.40)	0.075
3 + 4 + 5m-PFOS	**1.71 (1.39, 2.19)**	**<0.001**	**−0.10 (−0.12, −0.07)**	**<0.001**	**−0.29 (−0.36, −0.22)**	**<0.001**
∑m2-PFOS	**1.45 (1.12, 1.86)**	**0.004**	**−0.60 (−0.65, −0.54)**	**<0.001**	0.19 (−0.02, 0.40)	0.078
PFHpS	**1.65 (1.23, 2.22)**	**0.001**	**−0.61 (−0.67, −0.56)**	**<0.001**	0.18 (−0.03, 0.39)	0.099
Total PFHxS	1.30 (0.97, 1.77)	0.089	**−0.57 (−0.63, −0.51)**	**<0.001**	0.19 (−0.04, 0.41)	0.100
n-PFHxS	1.26 (0.95, 1.70)	0.125	**−0.56 (−0.62, −0.50)**	**<0.001**	0.19 (−0.03, 0.41)	0.087
Br-PFHxS	**1.24 (1.02, 1.53)**	**0.038**	**−0.28 (−0.33, −0.23)**	**<0.001**	−0.06 (−0.22, 0.09)	0.435
PFOA	**3.01 (1.96, 4.70)**	**<0.001**	**−0.58 (−0.65, −0.50)**	**<0.001**	−0.06 (−0.32, 0.21)	0.682
PFHpA	**1.26 (1.05, 1.52)**	**0.017**	**−0.24 (−0.28, −0.20)**	**<0.001**	0.05 (−0.09, 0.18)	0.514
PFHxA	1.06 (0.90, 1.24)	0.491	**−0.09 (−0.14, −0.05)**	**<0.001**	0.05 (−0.07, 0.17)	0.444
PFNA	**2.41 (1.70, 3.46)**	**<0.001**	**−0.82 (−0.88, −0.75)**	**<0.001**	0.21 (−0.05, 0.46)	0.116
PFDA	**2.18 (1.64, 2.94)**	**<0.001**	**−0.68 (−0.73, −0.62)**	**<0.001**	0.15 (−0.06, 0.36)	0.169
PFUnDA	**1.83 (1.40, 2.41)**	**<0.001**	**−0.62 (−0.67, −0.56)**	**<0.001**	0.12 (−0.08, 0.33)	0.226
PFDoDA	**1.51 (1.21, 1.92)**	**<0.001**	**−0.48 (−0.53, −0.43)**	**<0.001**	0.12 (−0.06, 0.29)	0.192
PFTrDA	**1.53 (1.19, 2.00)**	**0.001**	**−0.44 (−0.49, −0.38)**	**<0.001**	0.16 (−0.02, 0.34)	0.088

Adjusted for age, sex, education, alcohol drinking, smoking, family income, exercise, BMI, district. Bolded values indicate that associations were statistically significant (*p* < 0.05). ^a^ The PFAS concentrations were natural-log transformed. Abbreviations: ASMI, appendicular skeletal muscle mass index; GripI, grip index; n-PFOS, linear PFOS; Br-PFOS, branched PFOS.

**Table 4:** Mediating effects of liver function in the associations of serum PFOS and PFOA with sarcopenia.

PFAS	ALB	GLB	TP	ALT	AST	GGT
TotalPFOS	16.84 (−9.91, 49.56)	**16.14 (5.43, 30.20)**	−0.00 (−3.16, 2.67)	−0.09 (−5.37, 2.53)	4.46 (−1.04, 11.29)	**3.97 (0.41, 8.28)**
n-PFOS	16.61 (−7.87, 49.60)	**15.76 (4.86, 28.51)**	−0.03 (−2.73, 2.81)	0.02 (−3.94, 2.01)	4.02 (−0.46, 10.62)	**3.31 (0.25, 7.72)**
Br-PFOS	**17.42 (3.58, 39.56)**	**9.56 (4.61, 17.96)**	0.11 (−1.13, 1.61)	−0.24 (−5.33, 2.44)	3.53 (−0.37, 9.27)	**3.66 (0.01, 8.23)**
1m-PFOS	**81.74 (29.76, 247.31)**	**28.77 (11.14, 89.44)**	0.69 (−5.51, 6.54)	0.74 (−14.05, 10.02)	**12.01 (1.02, 42.78)**	**9.43 (1.21, 31.25)**
iso-PFOS	**46.43 (14.42, 108.60)**	**23.59 (10.36, 53.93)**	0.19 (−4.14, 4.79)	0.29 (−7.13, 4.80)	7.27 (−0.03, 19.62)	**6.08 (0.29, 13.44)**
3 + 4 + 5m-PFOS	**8.31 (2.38, 18.50)**	**3.48 (1.15, 8.27)**	0.00 (−0.47, 0.61)	−0.04 (−1.85, 1.20)	**1.55 (0.02, 4.68)**	**1.93 (0.09, 4.81)**
∑2-PFOS	**82.42 (36.07, 249.96)**	**36.15 (15.47, 133.52)**	0.90 (−4.48, 8.58)	0.67 (−12.00, 11.13)	**15.44 (1.23, 49.47)**	**10.85 (1.12, 28.57)**
PFOA	**22.31 (6.95, 46.18)**	**11.12 (5.41, 22.12)**	0.19 (−1.32, 2.22)	0.28 (−6.28, 3.88)	4.60 (−0.36, 11.30)	**3.79 (0.36, 7.69)**

The model was adjusted for age, sex, education, alcohol drinking, smoking, family income, exercise, BMI, and district. Bolded values indicate that associations were statistically significant (*p* < 0.05). Abbreviations: ALB, albumin; GLB, globin; TP, total protein; ALT, alanine aminotransferase; AST, aspartate aminotransferase; GGT, gamma-glutamyl transferase; ASMI, appendicular skeletal muscle mass index; GripI, grip index; n-PFOS, linear PFOS; Br-PFOS, branched PFOS.

## Data Availability

The raw data supporting the conclusions of this article will be made available by the authors on request.

## Supplementary Materials

The following supporting information can be downloaded at https://www.mdpi.com/article/10.3390/toxics14060478/s1, PFAS Measurement. Table S1: Abbreviations, detection rate, limit of detection (LOD) of PFAS (ng/mL) measured in this study (N = 1261). Table S2: The PFAS, muscle parameters, and biomarkers of liver function according to district (N = 1261). Table S3: PIPs for group inclusion and conditional inclusion using BKMR model (N = 1261). Table S4: The overall effects of PFAS mixtures on muscle parameters in the Bayesian kernel machine regression (BKMR) model which were shown in Figure 1. (a)–(c). Table S5: The individual effects of PFAS mixtures on muscle parameters in the Bayesian kernel machine regression (BKMR) model which were shown in Figure 1. (d)–(f). Table S6: Effect for the association between markers of liver function and sarcopenia (N = 1261). Table S7: Adjusted estimated change (β) and 95% confidence intervals (CIs) for markers of liver function with PFAS (N = 1261). Table S8: The mediation of markers of liver function in the association between sarcopenia and PFAS in serum (N = 1261). Table S9: The mediation of markers of liver function in the association between ASMI and PFAS in serum (N = 1261). Table S10: The mediation of markers of liver function in the association between GripI and PFAS in serum (N = 1261). Table S11: The mediation of markers of liver function in the association between sarcopenia and PFAS in serum excluding CKD, malnutrition, and inflammation (N = 989). Table S12: The mediation of markers of liver function in the association between sarcopenia and PFAS in serum excluding CHD, stroke, and dyslipidemia (N = 1155). Table S13: Odds ratios (ORs) and 95% confidence intervals (CIs) for association between sarcopenia and PFAS in serum by sex (N = 1261). Table S14: Odds ratios (ORs) and 95% confidence intervals (CIs) for association between sarcopenia and PFAS in serum by age (N = 1261). Table S15: Odds ratios (ORs) and 95% confidence intervals (CIs) for association between sarcopenia and PFAS in serum excluding 206 alcohol drinkers (N = 1055). Table S16: Odds ratios (ORs) and 95% confidence intervals (CIs) for association between sarcopenia and PFAS in serum excluding 270 smokers (N = 991). Table S17: Odds ratios (ORs) and 95% confidence intervals (CIs) for association between sarcopenia and PFAS in serum excluding 118 participants with diabetes (N = 1143). Table S18: Odds ratios (ORs) and 95% confidence intervals (CIs) for association between sarcopenia and PFAS in serum excluding 217 participants with osteoporosis (N = 1044). Table S19: Odds ratios (ORs) and 95% confidence intervals (CIs) for association between sarcopenia and PFAS in serum excluding 64 participants with malnutrition (N = 1197). Table S20: Odds ratios (ORs) and 95% confidence intervals (CIs) for association between sarcopenia and PFAS in serum excluding 35 participants with CHD and stroke (N = 1226). Table S21: Odds ratios (ORs) and 95% confidence intervals (CIs) for association between sarcopenia and PFAS in serum excluding 80 participants with dyslipidemia (N = 1181). Table S22: Odds ratios (ORs) and 95% confidence intervals (CIs) for association between sarcopenia and PFAS in serum excluding participants with PFAS levels below the limit of quantitation (LOQ) (N = 735). Figure S1: Spearman correlation coefficients among serum PFAS.
