# Supplementary material for: Exposure to Per- and Polyfluoroalkyl Substances and the Risk of Sarcopenia: The Mediating Role of Serum Albumin
Source: Toxics. 2026 May 29;14(6):478. doi: 10.3390/toxics14060478 (PMC13307807; doi:10.3390/toxics14060478)
Supplement: Supplementary file 1 [file toxics-14-00478-s001.zip › toxics-4253138-supplementary.pdf]

Exposure to Per- and Polyfluoroalkyl Substance and the Risk of Sarcopenia: The Mediating Role of Serum Albumin

Supplementary Information

Mingkun Sun [1,†], Chu Chu [1,†], Kun Zhao [1], Zhengmin (Min) Qian [2], Mario Schootman [3], Stephen Edward McMillin [4], Jiaxiang Dong [5], Wenwen Bao [1], Muhammad Amjad [1], Haseeb Tufail Moryani [1], Yang Zhou [1], Yan Yang [6,*] and Peipei Wang [7,*]

PFAS Measurement.. 3
Table S1 Abbreviations, detection rate, limit of detection (LOD) of PFAS (ng/mL) measured in this study (N = 1,261). 6
Table S2.The PFAS, muscle parameters, and biomarkers of liver function according to district (N = 1,261).8
Table S3. PIPs for group inclusion and conditional inclusion using BKMR model (N=1261)..................... 10
Table S4.The overall effects of PFAS mixtures on muscle parameters in the Bayesian kernel machine regression (BKMR) model which were shown in Figure 1. (a)-(c)*.. 11
Table S5.The individual effects of PFAS mixtures on muscle parameters in the Bayesian kernel machine regression (BKMR) model which were shown in Figure 1. (d)-(f)*.. 12
Table S6. Effect for the association between markers of liver function and sarcopenia (N=1,261)........... 14
Table S7. Adjusted estimated change (β) and 95% confidence intervals (CIs) for markers of liver function with PFAS (N=1,261).. 15
Table S8. The mediation of markers of liver function in the association between sarcopenia and PFAS in serum (N=1,261). 16
Table S9. The mediation of markers of liver function in the association between ASMI and PFAS in serum (N=1,261).. 21
Table S10. The mediation of markers of liver function in the association between GripI and PFAS in serum (N=1,261).. 26
Table S11. The mediation of markers of liver function in the association between sarcopenia and PFAS in serum excluding CKD, malnutrition, and inflammation (N=989). 31
Table S12. The mediation of markers of liver function in the association between sarcopenia and PFAS in serum excluding CHD, stroke, and dyslipidemia(N=1155). 36
Table S13. Odds ratios (ORs) and 95% confidence intervals (CIs) for association between sarcopenia and PFAS in serum by sex(N=1,261)... 41
Table S14. Odds ratios (ORs) and 95% confidence intervals (CIs) for association between sarcopenia and PFAS in serum by age (N=1261). 42
Table S15. Odds ratios (ORs) and 95% confidence intervals (CIs) for association between sarcopenia and PFAS in serum excluding 206 alcohol drinkers (N=1,055).. 43
Table S16. Odds ratios (ORs) and 95% confidence intervals (CIs) for association between sarcopenia and PFAS in serum excluding 270 smokers (N=991)... 44
Table S17. Odds ratios (ORs) and 95% confidence intervals (CIs) for association between sarcopenia and PFAS in serum excluding 118 participants with diabetes (N=1,143)... 45
Table S18. Odds ratios (ORs) and 95% confidence intervals (CIs) for association between sarcopenia and PFAS in serum excluding 217 participants with osteoporosis (N=1,044)... 46
Table S19. Odds ratios (ORs) and 95% confidence intervals (CIs) for association between sarcopenia and PFAS in serum excluding 64 participants with malnutrition (N=1,197).. 47
Table S20. Odds ratios (ORs) and 95% confidence intervals (CIs) for association between sarcopenia and PFAS in serum excluding 35 participants with CHD and stroke (N=1,226)... 48
Table S21. Odds ratios (ORs) and 95% confidence intervals (CIs) for association between sarcopenia and PFAS in serum excluding 80 participants with dyslipidemia (N=1,181). 49
Table S22. Odds ratios (ORs) and 95% confidence intervals (CIs) for association between sarcopenia and PFAS in serum excluding participants with PFAS levels below the limit of quantitation (LOQ) (N = 735).50
Figure S1 Spearman correlation coefficients among serum PFAS... 51

PFAS Measurement

Supplemental Methods for PFAS Measurement

Standards and reagents

All PFAS standards were purchased from Wellington Laboratories (Guelph, ON, Canada). A total of 32 PFASs, including 26 linear PFASs and 6 PFAS isomers, were measured in serum samples. The nomenclature of PFOS isomers followed that defined by Benskin et al. (2007). Detailed abbreviations of PFASs and the nomenclature of PFAS isomers are provided in Table S1.

High-performance liquid chromatography (HPLC)-grade formic acid and ammonium hydroxide (28.0-30.0%) were purchased from Sigma-Aldrich (Milwaukee, WI, USA). HPLC-grade methanol was obtained from Burdick & Jackson (Honeywell International Inc., USA), and HPLC-grade acetonitrile was purchased from Fisher Scientific (Thermo Fisher Scientific Inc., USA). Distilled water was prepared using a Milli-Q water purification system (EPED, China).

Sample extraction

For each sample, 0.2 mL of serum was mixed with 2 mL of 0.1 M formic acid, followed by the addition of 25 μL of a 20 ppb internal standard mixture. Method blanks and spiked recovery samples were prepared using the same procedure and were also supplemented with 25 μL of the 20 ppb internal standard mixture.

Solid-phase extraction was performed using HLB cartridges. Each cartridge was conditioned sequentially with 2 mL of methanol and 2 mL of 0.1 M formic acid. The prepared sample was then loaded onto the cartridge. The cartridge was washed sequentially with 3 mL of 0.1 M formic acid, 6 mL of 50% 0.1 M formic acid/50% methanol, and 1 mL of 1% ammonium hydroxide. Residual liquid in the cartridge was removed under vacuum. PFASs were eluted into a 15 mL centrifuge tube with 2 mL of 1% ammonium hydroxide in acetonitrile, followed by vacuum-assisted drying.

The eluate was evaporated to near dryness under nitrogen at 40°C. The residue was reconstituted with 70 μL of methanol and 30 μL of 20 mM ammonium formate, followed by vortex mixing to dissolve any precipitate. The sample was centrifuged at 4,500 rpm for 10 min at 4°C, and the supernatant was transferred to a microcentrifuge tube. After a second centrifugation at 12,000 rpm for 10 min at 4°C, 20 μL of the supernatant was transferred to an autosampler vial for instrumental analysis.

UPLC-MS/MS analysis

PFASs were analyzed using ultra-performance liquid chromatography coupled with tandem mass spectrometry (UPLC-MS/MS). Electrospray ionization was operated in negative ion mode. The source temperature was set at 100°C, the ionization voltage at 4,000 V, the nebulizing gas was nitrogen, the gas temperature was 350°C, the gas flow rate was 11 L/min, and the nebulizing gas pressure was 40 psi. Quantitative detection was performed in multiple reaction monitoring (MRM) mode.

The mobile phase consisted of 20 mM ammonium formate as solvent A and 100% methanol

as solvent B, with a flow rate of 0.3 mL/min. The autosampler temperature was maintained at 4°C, the injection volume was 5 μL, and the column oven temperature was set at 38°C. Gradient elution was performed as follows: the initial condition of 55% A and 45% B was maintained for 3 min; solvent B was increased to 60% at 3 min, 63% at 15 min, and 95% at 15.5 min; this condition was maintained until 18.5 min. The gradient was then returned to the initial condition at 19 min and maintained for 4 min for column re-equilibration.

Quality control and quality assurance

To minimize background signals and reduce exogenous contamination, all experimental materials, including pipette tips, microcentrifuge tubes, centrifuge tubes, autosampler vials, and vial septa, were soaked in methanol for more than 4 h before use.

PFAS concentrations in serum samples were determined using the internal standard quantification method, and calibration curves prepared with internal standards were used to correct sample concentrations. Nine calibration points ranging from 0.05 to 100 ng/mL were used, and the coefficient of determination (R^2) for each analyte was required to be greater than 0.99.

For each batch of 22 samples, one method blank and three reagent blanks were included to monitor potential exogenous contamination. The method blank underwent the same pretreatment procedure as the serum samples but without serum addition. The reagent blanks consisted of 70% methanol and were analyzed to monitor potential contamination from mobile-phase reagents or residual PFASs in the instrume

Table S1 Abbreviations, detection rate, limit of detection (LOD) of PFAS (ng/mL) measured in this study (N = 1,261).

Abbreviation	Full name	Detection rate (%)	LOD (ng/mL)	LOQ (ng/mL)	Recovery (%) (RSD%)
Total PFOS	Sum of linear and branched perfluoro-1-octane sulfonate	**100.00**	-	-	-
n-PFOS	Perfluoro-1-octane sulfonate	**100.00**	0.0016	0.0055	86.2 ± 11.4 (6.8)
Br-PFOS	Sum of all branched isomers PFOS	**100.00**	-	-	-
1m-PFOS	Linear perfluoro-1-methyl-hptanesulfonate	**100.00**	0.0003	0.0009	109.0 ± 19.4 (9.0)
iso-PFOS	Perfluoro-6-methyl-heptanesulfonate	**100.00**	0.0010	0.0034	106.0 ± 7.3 (11.0)
Σ3 + 4 + 5m-PFOS	Sum of 3m, 4m and 5m-PFOS	**92.15**	0.0013	0.0044	109.0 ± 8.6 (8.1)
Σm2-PFOS	Sum of all dimethyl isomers PFOS	**100.00**	0.0008	0.0026	142.0 ± 29.5 (17.6)
PFBS	Perfluoro-1-butansulfonic acid	59.87	0.0095	0.0316	101.2 ± 8.2 (2.2)
PFPeS	Perfluoro-1-pentanesulfonic acid	54.32	0.0029	0.0097	97.6 ± 7.3 (5.1)
PFHpS	Perfluoro-1-heptanesulfonic acid	**99.84**	0.0021	0.0069	102.6 ± 8.3 (5.6)
Total PFHxS	Sum of linear and branched perfluoro-1-hexane sulfonate	**100.00**	-	-	-
n-PFHxS	Linear perfluoro-1-hexane sulfonate	**100.00**	0.0011	0.0037	102.0 ± 15.3 (4.6)
Br-PFHxS	Sum of all branched isomers PFHxS	**94.87**	0.0011	0.0037	101.9 ± 10.3 (5.8)
PFNS	Perfluoro-1-nonane sulfonic acid	31.88	0.0060	0.0198	94.9 ± 9.3 (7.0)
PFDS	Perfluoro-1-decane sulfonic acid	28.79	0.0038	0.0125	80.1 ± 19.7 (6.8)
PFOA	Perfluoro-n-octanoic acid	**100.00**	0.0025	0.0084	99.1 ± 10.5 (1.2)
PFBA	Perfluoro-n-nbutanoic acid	27.99	0.0081	0.0271	102.0 ± 16.5 (1.5)
PFPeA	Perfluoro-n-pentanoic acid	6.34	0.0179	0.0598	103.1 ± 8.2 (1.6)
PFHxA	Perfluoro-n-hexanoic acid	**86.04**	0.0035	0.0117	77.9 ± 1.3 (1.7)
PFHpA	Perfluoro-n-heptanoic acid	**94.53**	0.0016	0.0052	100.2 ± 9.1 (1.7)
PFNA	Perfluoro-n-nonaoic acid	**100.00**	0.0024	0.008	105.0 ± 17.7 (2.0)
PFDA	Perfluoro-n-decanoic acid	**100.00**	0.0048	0.0159	111.0 ± 11.1 (1.2)

Abbreviation	Full name	Detection rate (%)	LOD (ng/mL)	LOQ (ng/mL)	Recovery (%) (RSD%)
PFUnDA	Perfluoro-n-undecanoic acid	**100**	0.0049	0.0163	76.9 ± 7.7 (2.4)
PFDoDA	Perfluoro-n-dodecanoic acid	**95.48**	0.0057	0.0189	87.7 ± 20.9 (3.3)
PFTrDA	Perfluoro-n-tridecanoic acid	**98.73**	0.0106	0.0352	121.7 ± 10.3 (3.3)
PFTeDA	Perfluoro-n-tetradecanoic acid	78.75	0.0036	0.0121	102.3 ± 9.2 (4.3)
HFPO-DA	Hexafluoro-1-propylene oxide dimer acid	12.37	0.5025	1.6752	75.3 ± 10.6 (6.0)
FOSA	Perfluoro-1-octanesulfonamide	69.39	0.0011	0.0037	99.7 ± 8.7 (2.0)
N-MeFOSAA	N-methylperfluoro-1-octanesulfonamidoacetic acid	29.98	0.0044	0.0148	103.8 ± 15.3 (5.7)
N-EtFOSAA	N-ethylperfluoro-1-octanesulfonamidoacetic acid	41.40	0.0016	0.0054	104.9 ± 9.5 (4.8)
4:2FTSA	1H, 1H, 2H, 2H-perfluoro-1-hexanesulfonic acid	6.26	0.0073	0.0243	100.5 ± 8.5 (2.0)
6:2FTSA	1H, 1H, 2H, 2H-perfluoro-1-octanesulfonic acid	7.69	0.0106	0.0354	100.3 ± 9.4 (2.3)
8:2FTSA	1H, 1H, 2H, 2H-perfluoro-1-decanesulfonic acid	12.05	0.0017	0.0058	101.6 ± 17.5 (2.6)

Table S2.The PFAS, muscle parameters, and biomarkers of liver function according to district (N = 1,261).

Variables	Overall N = 1,261	Conghua N = 233	Panyu N = 719	Yuexiu N = 309	P
Total PFOS	14.66 (8.30, 25.39)	9.23 (5.00, 16.86)	17.97 (10.13, 30.15)	14.07 (8.16, 19.52)	<0.001
n-PFOS	10.83 (6.21, 18.77)	6.48 (3.83, 13.23)	13.64 (7.33, 23.22)	10.38 (6.27, 15.29)	<0.001
Br-PFOS	3.66 (1.79, 6.16)	1.91 (0.87, 3.97)	4.54 (2.25, 7.56)	3.27 (1.79, 5.30)	<0.001
1m-PFOS	0.49 (0.24, 0.89)	0.26 (0.10, 0.46)	0.58 (0.29, 1.04)	0.51 (0.29, 0.77)	<0.001
iso-PFOS	0.74 (0.42, 1.40)	0.40 (0.20, 0.71)	0.98 (0.55, 1.82)	0.63 (0.44, 1.05)	<0.001
3 + 4 + 5m-PFOS[a]	2.35 (0.96, 4.11)	1.29 (0.37, 2.75)	2.88 (1.40, 4.85)	2.03 (0.82, 3.48)	<0.001
∑m2-PFOS[a]	0.04 (0.02, 0.07)	0.02 (0.01, 0.04)	0.05 (0.03, 0.08)	0.03 (0.02, 0.05)	<0.001
PFHpS	0.89 (0.49, 1.39)	0.64 (0.27, 1.05)	0.88 (0.51, 1.36)	1.13 (0.66, 1.65)	<0.001
Total PFHxS	0.87 (0.47, 1.37)	0.62 (0.26, 1.03)	0.86 (0.49, 1.33)	1.09 (0.64, 1.62)	<0.001
n-PFHxS	0.02 (0.01, 0.03)	0.01 (0.00, 0.02)	0.02 (0.01, 0.03)	0.03 (0.02, 0.04)	<0.001
Br-PFHxS	0.33 (0.17, 0.51)	0.24 (0.09, 0.39)	0.35 (0.19, 0.54)	0.35 (0.21, 0.49)	<0.001
PFOA	8.93 (5.37, 13.53)	2.82 (1.48, 4.56)	12.33 (8.81, 15.56)	6.67 (5.22, 9.14)	<0.001
PFHpA	0.04 (0.01, 0.07)	0.02 (0.00, 0.04)	0.03 (0.01, 0.06)	0.08 (0.04, 0.12)	<0.001
PFHxA	0.03 (0.02, 0.06)	0.02 (0.01, 0.04)	0.04 (0.02, 0.07)	0.03 (0.02, 0.05)	<0.001
PFNA	1.11 (0.69, 1.65)	0.83 (0.51, 1.37)	1.19 (0.75, 1.78)	1.14 (0.76, 1.60)	<0.001
PFDA	0.84 (0.52, 1.40)	0.69 (0.38, 1.15)	0.93 (0.57, 1.56)	0.82 (0.54, 1.22)	<0.001
PFUnDA	0.73 (0.44, 1.14)	0.64 (0.34, 0.97)	0.76 (0.46, 1.20)	0.70 (0.45, 1.07)	0.001
PFDoDA	0.06 (0.03, 0.10)	0.06 (0.03, 0.09)	0.06 (0.03, 0.10)	0.06 (0.04, 0.09)	0.131
PFTrDA	0.27 (0.16, 0.44)	0.25 (0.14, 0.41)	0.31 (0.18, 0.46)	0.23 (0.14, 0.37)	<0.001
ASMI, Mean ± SD,	6.81 ± 1.36	7.23 ± 1.18	6.59 ± 1.28	6.99 ± 1.56	<0.001

kg/m^2					
Grip, Mean ± SD, kg	10.82 ± 3.35	11.61 ± 4.33	10.33 ± 3.06	11.37 ± 2.94	<0.001
ALB, Mean ± SD, g/L	48.34 ± 4.49	46.92 ± 4.54	49.04 ± 4.51	47.80 ± 4.05	<0.001
GLB, Mean ± SD, g/L	29.80 ± 4.30	28.22 ± 3.66	29.89 ± 4.46	30.80 ± 4.02	<0.001
TP, Mean ± SD, g/L	77.73 ± 4.03	77.66 ± 3.89	77.54 ± 4.16	78.24 ± 3.79	0.012
ALT, Mean ± SD, U/L	21.49 ± 15.93	20.51 ± 18.88	21.88 ± 15.90	21.33 ± 13.41	0.01
AST, Mean ± SD, U/L	22.32 ± 19.39	22.17 ± 8.57	23.21 ± 24.79	20.39 ± 6.72	0.001
GGT, Mean ± SD, U/L	32.83 ± 53.65	28.31 ± 26.93	35.86 ± 67.44	29.20 ± 24.04	0.012

Abbreviations: ASMI, Appendicular Skeletal Muscle Mass Index; ALB, albumin; GLB, globin; TP, total protein; ALT, Alanine aminotransferase; AST, Aspartate Aminotransferase; GGT, Gamma-Glutamyl Transferase.

Table S3. PIPs for group inclusion and conditional inclusion using BKMR model (N=1261).

PFAS	Group	Sarcopenia	ASMI	GripI
		Group PIP (cond PIP)	Group PIP (cond PIP)	Group PIP (cond PIP)
n-PFOS	1	**1.0000 (0.4052)**	**1.0000 (1.0000)**	1.0000 (0.0000)
1m-PFOS	1	1.0000 (0.0000)	1.0000 (0.0000)	1.0000 (0.0000)
iso-PFOS	1	1.0000 (0.0000)	1.0000 (0.0000)	1.0000 (0.0000)
3 + 4 + 5m-PFOS	1	1.0000 (0.5838)	1.0000 (0.0000)	**1.0000 (1.0000)**
∑m2-PFOS	1	1.0000 (0.0000)	1.0000 (0.0000)	1.0000 (0.0000)
PFHpS	2	0.9814 (0.7338)	1.0000 (0.9650)	1.0000 (0.3548)
n-PFHxS	2	0.9814 (0.0110)	1.0000 (0.0000)	1.0000 (0.0000)
Br-PFHxS	2	0.9814 (0.2551)	1.0000 (0.0350)	1.0000 (0.6452)
PFOA	3	**1.0000 (1.0000)**	**1.0000 (1.0000)**	0.5430 (0.0000)
PFHpA	3	1.0000 (0.0000)	1.0000 (0.0000)	0.5430 (0.8755)
PFHxA	3	1.0000 (0.0000)	1.0000 (0.0000)	0.5430 (0.0884)
PFNA	3	1.0000 (0.0000)	1.0000 (0.0000)	0.5430 (0.0000)
PFDA	3	1.0000 (0.0000)	1.0000 (0.0000)	0.5430 (0.0026)
PFUnDA	3	1.0000 (0.0000)	1.0000 (0.0000)	0.5430 (0.0099)
PFDoDA	3	1.0000 (0.0000)	1.0000 (0.0000)	0.5430 (0.0000)
PFTrDA	3	1.0000 (0.0000)	1.0000 (0.0000)	0.5430 (0.0236)

Abbreviations: BKMR, Bayesian kernel machine regression; PIP, posterior inclusion probability; condPIP, conditional posterior inclusion probability.

Adjusted for age, sex, education, alcohol drinking, smoking, family income, exercise, BMI, and district.

Table S4. The overall effects of PFAS mixtures on muscle parameters in the Bayesian kernel machine regression (BKMR) model which were shown in Figure 1. (a)-(c)*

Quantile	Sarcopenia<br>OR (95% CI)	ASMI<br>β (95% CI)	GripI<br>β (95% CI)
25 th	0.76 (0.69, 0.83)	0.64 (0.58, 0.70)	1.01 (0.72, 1.31)
30 th	0.80 (0.75, 0.86)	0.49 (0.45, 0.53)	0.82 (0.60, 1.03)
35 th	0.85 (0.81, 0.89)	0.35 (0.32, 0.38)	0.69 (0.53, 0.84)
40 th	0.90 (0.87, 0.94)	0.23 (0.20, 0.25)	0.43 (0.34, 0.53)
45 th	0.97 (0.95, 0.98)	0.10 (0.09, 0.12)	0.17 (0.12, 0.22)
50 th	1.00 (1.00, 1.00)	0.00 (0.00, 0.00)	0.00 (0.00, 0.00)
55 th	1.07 (1.05, 1.09)	-0.13 (-0.14, -0.11)	-0.16 (-0.20, -0.11)
60 th	1.14 (1.10, 1.18)	-0.24 (-0.26, -0.22)	-0.24 (-0.32, -0.16)
65 th	1.23 (1.16, 1.30)	-0.37 (-0.40, -0.33)	-0.42 (-0.54, -0.30)
70 th	1.31 (1.22, 1.40)	-0.46 (-0.51, -0.42)	-0.47 (-0.63, -0.32)
75 th	1.39 (1.27, 1.52)	-0.57 (-0.62, -0.51)	-0.57 (-0.77, -0.38)

Abbreviations: BKMR, Bayesian kernel machine regression; ASMI, Appendicular Skeletal Muscle Mass Index.

Adjusted for age, sex, education, alcohol drinking, smoking, family income, exercise, BMI, and district.

Table S5. The individual effects of PFAS mixtures on muscle parameters in the Bayesian kernel machine regression (BKMR) model which were shown in Figure 1. (d)-(f)*

PFAS	Quantile	Sarcopenia<br>OR (95% CI)	ASMI<br>β (95% CI)	GripI<br>β (95% CI)
	25 th	1.75 (1.24, 2.48)	-0.95 (-1.07, -0.82)	0.00 (0.00, 0.00)
n-PFOS	50 th	1.86 (1.38, 2.51)	-0.80 (-0.89, -0.71)	0.00 (0.00, 0.00)
	75 th	1.95 (1.34, 2.85)	-0.67 (-0.77, -0.56)	0.00 (0.00, 0.00)
	25 th	1.00 (1.00, 1.00)	0.00 (0.00, 0.00)	0.00 (0.00, 0.00)
1m-PFOS	50 th	1.00 (1.00, 1.00)	0.00 (0.00, 0.00)	0.00 (0.00, 0.00)
	75 th	1.00 (1.00, 1.00)	0.00 (0.00, 0.00)	0.00 (0.00, 0.00)
	25 th	1.00 (1.00, 1.00)	0.00 (0.00, 0.00)	0.00 (0.00, 0.00)
iso-PFOS	50 th	1.00 (1.00, 1.00)	0.00 (0.00, 0.00)	0.00 (0.00, 0.00)
	75 th	1.00 (1.00, 1.00)	0.00 (0.00, 0.00)	0.00 (0.00, 0.00)
	25 th	1.00 (1.00, 1.00)	0.00 (0.00, 0.00)	-4.82 (-5.53, -4.11)
3 + 4 + 5m-PFOS	50 th	1.00 (1.00, 1.00)	0.00 (0.00, 0.00)	-3.88 (-4.39, -3.38)
	75 th	1.00 (1.00, 1.00)	0.00 (0.00, 0.00)	-3.18 (-3.70, -2.65)
	25 th	1.00 (1.00, 1.00)	0.00 (0.00, 0.00)	0.00 (0.00, 0.00)
∑m2-PFOS	50 th	1.00 (1.00, 1.00)	0.00 (0.00, 0.00)	0.00 (0.00, 0.00)
	75 th	1.00 (1.00, 1.00)	0.00 (0.00, 0.00)	0.00 (0.00, 0.00)
	25 th	0.84 (0.53, 1.34)	-0.00 (-0.06, 0.06)	1.68 (1.18, 2.17)
PFHpS	50 th	0.83 (0.52, 1.34)	-0.00 (-0.03, 0.03)	2.76 (2.34, 3.18)
	75 th	0.85 (0.54, 1.34)	0.00 (-0.03, 0.03)	3.31 (2.76, 3.86)
	25 th	0.85 (0.60, 1.21)	-0.28 (-0.41, -0.15)	0.00 (0.00, 0.00)
n-PFHxS	50 th	0.82 (0.57, 1.19)	-0.12 (-0.21, -0.02)	0.00 (0.00, 0.00)
	75 th	0.81 (0.54, 1.21)	0.04 (-0.08, 0.16)	0.00 (0.00, 0.00)
	25 th	1.00 (1.00, 1.00)	0.00 (0.00, 0.00)	0.00 (0.00, 0.00)
Br-PFHxS	50 th	1.00 (1.00, 1.00)	0.00 (0.00, 0.00)	0.00 (0.00, 0.00)
	75 th	1.00 (1.00, 1.00)	0.00 (0.00, 0.00)	0.00 (0.00, 0.00)
	25 th	1.28 (0.98, 1.69)	-0.34 (-0.44, -0.25)	0.00 (0.00, 0.00)
PFOA	50 th	1.42 (1.15, 1.76)	-0.26 (-0.34, -0.19)	0.00 (0.00, 0.00)
	75 th	1.59 (1.26, 2.02)	-0.18 (-0.27, -0.08)	0.00 (0.00, 0.00)
	25 th	1.00 (1.00, 1.00)	0.00 (0.00, 0.00)	0.00 (-0.02, 0.02)
PFHpA	50 th	1.00 (1.00, 1.00)	0.00 (0.00, 0.00)	-0.00 (-0.02, 0.02)
	75 th	1.00 (1.00, 1.00)	0.00 (0.00, 0.00)	-0.00 (-0.02, 0.01)
	25 th	1.00 (1.00, 1.00)	0.00 (0.00, 0.00)	0.00 (0.00, 0.00)
PFHxA	50 th	1.00 (1.00, 1.00)	0.00 (0.00, 0.00)	0.00 (0.00, 0.00)
	75 th	1.00 (1.00, 1.00)	0.00 (0.00, 0.00)	0.00 (0.00, 0.00)
	25 th	1.00 (1.00, 1.00)	0.00 (0.00, 0.00)	-0.01 (-0.13, 0.12)
PFNA	50 th	1.00 (1.00, 1.00)	0.00 (0.00, 0.00)	0.01 (-0.12, 0.13)
	75 th	1.00 (1.00, 1.00)	0.00 (0.00, 0.00)	0.02 (-0.21, 0.25)
	25 th	1.00 (1.00, 1.00)	0.00 (0.00, 0.00)	0.00 (0.00, 0.00)
PFDA	50 th	1.00 (1.00, 1.00)	0.00 (0.00, 0.00)	0.00 (0.00, 0.00)
	75 th	1.00 (1.00, 1.00)	0.00 (0.00, 0.00)	0.00 (0.00, 0.00)
PFUnDA	25 th	1.00 (1.00, 1.00)	0.00 (0.00, 0.00)	-0.00 (-0.04, 0.04)

	50 th	1.00 (1.00, 1.00)	0.00 (0.00, 0.00)	0.00 (-0.04, 0.04)
	75 th	1.00 (1.00, 1.00)	0.00 (0.00, 0.00)	0.00 (-0.06, 0.06)
	25 th	1.00 (1.00, 1.00)	0.00 (0.00, 0.00)	-0.00 (-0.07, 0.07)
PFDoDA	50 th	1.00 (1.00, 1.00)	0.00 (0.00, 0.00)	-0.00 (-0.03, 0.03)
	75 th	1.00 (1.00, 1.00)	0.00 (0.00, 0.00)	0.00 (-0.03, 0.03)
	25 th	1.00 (1.00, 1.00)	0.00 (0.00, 0.00)	-0.00 (-0.03, 0.03)
PFTrDA	50 th	1.00 (1.00, 1.00)	0.00 (0.00, 0.00)	0.00 (-0.02, 0.02)
	75 th	1.00 (1.00, 1.00)	0.00 (0.00, 0.00)	0.00 (-0.03, 0.04)

Abbreviations: BKMR, Bayesian kernel machine regression; ASMI, Appendicular Skeletal Muscle Mass Index.

Adjusted for age, sex, education, alcohol drinking, smoking, family income, exercise, BMI, and district.

Table S6. Effect for the association between markers of liver function and sarcopenia (N=1,261).

Markers	Sarcopenia	ASMI	GripI
	OR (95% CI)	β (95% CI)	
ALB	**1.12 (1.07, 1.18)**	**-0.14 (-0.15, -0.13)**	0.04 (-0.00, 0.08)
GLB	**1.09 (1.05, 1.15)**	**-0.08 (-0.09, -0.07)**	0.01 (-0.02, 0.05)
TP	1.01 (0.97, 1.06)	**-0.02 (-0.04, -0.01)**	0.03 (-0.01, 0.07)
ALT	1.00 (0.99, 1.02)	-0.00 (-0.01, 0.00)	0.00 (-0.01, 0.02)
AST	**1.06 (1.02, 1.09)**	**-0.03 (-0.04, -0.02)**	0.01 (-0.02, 0.04)
GGT	**1.01 (1.01, 1.02)**	**-0.01 (-0.01, -0.00)**	-0.00 (-0.00, 0.00)

Abbreviations: ALB, albumin; GLB, globin; TP, total protein; ALT, Alanine aminotransferase; AST, Aspartate Aminotransferase; GGT, Gamma-Glutamyl Transferase.

Adjusted model was adjusted for age, sex, education, alcohol drinking, smoking, family income, exercise, BMI, district.

Bolding indicates that associations were statistically significant ($P < 0{\cdot}05$).

Table S7. Adjusted estimated change (β) and 95% confidence intervals (CIs) for markers of liver function with PFAS (N=1,261).

PFAS[a] (ng/mL)	ALB	GLB	TP	ALT	AST	GGT
Total PFOS	**3.90 (3.65, 4.15)**	**1.90 (1.61, 2.19)**	**0.47 (0.18, 0.77)**	**1.62 (0.47, 2.76)**	**0.98 (0.55, 1.40)**	**3.44 (1.90, 4.99)**
n-PFOS	**3.45 (3.22, 3.67)**	**1.74 (1.48, 1.99)**	**0.42 (0.15, 0.69)**	**1.07 (0.05, 2.10)**	**0.77 (0.38, 1.15)**	**2.72 (1.32, 4.11)**
Br-PFOS	**2.01 (1.79, 2.24)**	**0.80 (0.57, 1.03)**	0.15 (-0.08, 0.38)	**1.58 (0.71, 2.46)**	**0.65 (0.32, 0.98)**	**2.66 (1.47, 3.85)**
1m-PFOS	**2.91 (2.68, 3.14)**	**1.15 (0.89, 1.40)**	**0.35 (0.09, 0.60)**	**2.41 (1.45, 3.38)**	**1.00 (0.64, 1.37)**	**3.12 (1.80, 4.44)**
iso-PFOS	**3.44 (3.20, 3.67)**	**1.54 (1.27, 1.81)**	**0.43 (0.16, 0.70)**	**1.66 (0.62, 2.71)**	**0.95 (0.55, 1.34)**	**3.37 (1.95, 4.79)**
3 + 4 + 5m-PFOS	**0.47 (0.36, 0.58)**	**0.18 (0.07, 0.28)**	0.00 (-0.10, 0.10)	0.35 (-0.04, 0.74)	**0.15 (0.00, 0.30)**	**0.94 (0.40, 1.47)**
∑m2-PFOS	**3.15 (2.89, 3.41)**	**1.39 (1.11, 1.67)**	**0.39 (0.10, 0.67)**	**2.04 (0.96, 3.12)**	**1.27 (0.87, 1.68)**	**3.65 (2.18, 5.11)**
PFHpS	**3.40 (3.17, 3.64)**	**1.25 (0.98, 1.52)**	**0.45 (0.18, 0.72)**	**2.39 (1.35, 3.43)**	**1.11 (0.72, 1.51)**	**3.72 (2.30, 5.14)**
Total-PFHxS	**3.09 (2.81, 3.37)**	**1.07 (0.76, 1.37)**	**0.31 (0.02, 0.61)**	**2.46 (1.33, 3.60)**	**1.13 (0.70, 1.56)**	**3.52 (1.98, 5.07)**
n-PFHxS	**3.03 (2.75, 3.30)**	**1.07 (0.77, 1.36)**	**0.34 (0.05, 0.64)**	**2.45 (1.34, 3.56)**	**1.12 (0.70, 1.54)**	**3.45 (1.93, 4.96)**
Br-PFHxS	**1.55 (1.33, 1.78)**	**0.49 (0.27, 0.72)**	0.08 (-0.14, 0.30)	0.81 (-0.03, 1.65)	**0.62 (0.30, 0.93)**	**1.50 (0.36, 2.65)**
PFOA	**2.84 (2.48, 3.19)**	**1.22 (0.87, 1.57)**	0.29 (-0.06, 0.63)	**2.64 (1.31, 3.96)**	**1.19 (0.69, 1.69)**	**3.14 (1.33, 4.96)**
PFHpA	**1.41 (1.21, 1.61)**	**0.47 (0.28, 0.67)**	**0.25 (0.06, 0.45)**	**1.31 (0.57, 2.05)**	**0.62 (0.34, 0.90)**	**2.30 (1.29, 3.30)**
PFHxA	**0.37 (0.18, 0.56)**	**0.22 (0.04, 0.40)**	0.10 (-0.08, 0.27)	0.18 (-0.49, 0.86)	**0.32 (0.06, 0.57)**	0.28 (-0.64, 1.20)
PFNA	**4.07 (3.78, 4.37)**	**1.80 (1.47, 2.13)**	**0.47 (0.13, 0.80)**	**1.77 (0.48, 3.05)**	**1.08 (0.60, 1.57)**	**4.76 (3.02, 6.50)**
PFDA	**3.21 (2.96, 3.46)**	**1.57 (1.30, 1.85)**	**0.29 (0.01, 0.57)**	0.54 (-0.54, 1.61)	**0.58 (0.18, 0.99)**	**2.45 (1.00, 3.91)**
PFUnDA	**2.77 (2.51, 3.03)**	**1.40 (1.13, 1.67)**	0.21 (-0.06, 0.49)	0.21 (-0.83, 1.26)	**0.46 (0.07, 0.85)**	**2.02 (0.60, 3.44)**
PFDoDA	**2.44 (2.22, 2.67)**	**1.06 (0.82, 1.29)**	0.11 (-0.13, 0.35)	0.33 (-0.59, 1.26)	**0.39 (0.04, 0.74)**	**1.34 (0.08, 2.59)**
PFTrDA	**2.03 (1.78, 2.29)**	**1.03 (0.78, 1.28)**	0.12 (-0.13, 0.37)	0.50 (-0.46, 1.46)	0.35 (-0.01, 0.71)	**1.33 (0.03, 2.64)**

Abbreviations: ALB, albumin; GLB, globin; TP, total protein; ALT, Alanine aminotransferase; AST, Aspartate Aminotransferase; GGT, Gamma-Glutamyl Transferase.

Model was adjusted for age, sex, education, alcohol drinking, smoking, family income, exercise, BMI, district.

Bolding indicates that associations were statistically significant ($P < 0{\cdot}05$).

[a] The variables were natural-log transformed.

Table S8. The mediation of markers of liver function in the association between sarcopenia and PFAS in serum (N=1,261).

Pathways	Total effect	Direct effect	Indirect effect	Prop mediated
**PFAS[a]->ALB->Sarcopenia**				
Total PFOS	**0.0144 (0.0089, 0.0192)**	**0.0120 (0.0065, 0.0177)**	0.0024 (-0.0014, 0.0081)	16.8445 (-9.9113, 49.5615)
n-PFOS	**0.0194 (0.0136, 0.0233)**	**0.0162 (0.0099, 0.0220)**	0.0032 (-0.0014, 0.0102)	16.6113 (-7.8728, 49.6017)
Br-PFOS	**0.0411 (0.0303, 0.0483)**	**0.0340 (0.0199, 0.0431)**	**0.0072 (0.0015, 0.0141)**	**17.4214 (3.5761, 39.5584)**
1m-PFOS	**0.0405 (0.0124, 0.0821)**	0.0074 (-0.0226, 0.0493)	**0.0331 (0.0166, 0.0519)**	**81.7376 (29.7645, 247.3071)**
iso-PFOS	**0.0613 (0.0308, 0.0945)**	0.0328 (-0.0035, 0.0708)	**0.0285 (0.0098, 0.0517)**	**46.4319 (14.4213, 108.6040)**
3 + 4 + 5m-PFOS	**0.0358 (0.0213, 0.0563)**	**0.0328 (0.0183, 0.0543)**	**0.0030 (0.0011, 0.0050)**	**8.3090 (2.3788, 18.4970)**
∑m2-PFOS	**0.0648 (0.0170, 0.1250)**	0.0114 (-0.0272, 0.0692)	**0.0534 (0.0234, 0.0843)**	**82.4185 (36.0714, 249.9645)**
PFHpS	**0.0544 (0.0170, 0.1175)**	0.0119 (-0.0277, 0.0730)	**0.0425 (0.0194, 0.0719)**	**78.0627 (26.7551, 241.7180)**
Total PFHxS	0.0225 (-0.0009, 0.0539)	-0.0118 (-0.0362, 0.0208)	**0.0343 (0.0186, 0.0514)**	152.6156 (-623.6439, 822.1754)
n-PFHxS	0.0198 (-0.0007, 0.0509)	-0.0150 (-0.0380, 0.0184)	**0.0348 (0.0195, 0.0499)**	175.8203 (-725.2339, 1119.2336)
Br-PFHxS	**0.0333 (0.0047, 0.0770)**	0.0105 (-0.0131, 0.0523)	**0.0228 (0.0115, 0.0357)**	**68.4757 (26.8086, 283.2690)**
PFOA	**0.0243 (0.0164, 0.0294)**	**0.0189 (0.0121, 0.0234)**	**0.0054 (0.0014, 0.0124)**	**22.3148 (6.9480, 46.1816)**
PFHpA	**0.0317 (0.0052, 0.0742)**	0.0123 (-0.0099, 0.0516)	**0.0194 (0.0095, 0.0301)**	**61.2974 (25.1661, 264.7031)**
PFHxA	0.0059 (-0.0063, 0.0309)	0.0019 (-0.0085, 0.0255)	**0.0040 (0.0014, 0.0077)**	**67.2753 (-551.3029, 489.9159)**
PFNA	**0.0830 (0.0487, 0.1239)**	**0.0553 (0.0148, 0.1013)**	**0.0277 (0.0063, 0.0504)**	**33.3655 (6.6102, 74.6920)**
PFDA	**0.0804 (0.0504, 0.1166)**	**0.0590 (0.0262, 0.1006)**	**0.0214 (0.0034, 0.0389)**	**26.5745 (4.4305, 53.1362)**
PFUnDA	**0.0628 (0.0327, 0.1025)**	**0.0385 (0.0065, 0.0814)**	**0.0242 (0.0100, 0.0396)**	**38.6336 (13.6302, 82.6684)**
PFDoDA	**0.0631 (0.0269, 0.1217)**	0.0282 (-0.0043, 0.0868)	**0.0349 (0.0150, 0.0563)**	**55.2877 (19.8987, 112.3683)**
PFTrDA	**0.0467 (0.0161, 0.0921)**	0.0233 (-0.0056, 0.0679)	**0.0233 (0.0115, 0.0368)**	**49.9984 (21.0529, 129.0202)**
**PFAS[a]->GLB->Sarcopenia**				
Total PFOS	**0.0145 (0.0094, 0.0192)**	**0.0121 (0.0081, 0.0162)**	**0.0023 (0.0006, 0.0053)**	**16.1411 (5.4324, 30.2024)**
n-PFOS	**0.0190 (0.0141, 0.0234)**	**0.0160 (0.0121, 0.0202)**	**0.0030 (0.0009, 0.0061)**	**15.7621 (4.8645, 28.5072)**
Br-PFOS	**0.0420 (0.0309, 0.0500)**	**0.0380 (0.0261, 0.0457)**	**0.0040 (0.0019, 0.0067)**	**9.5630 (4.6072, 17.9595)**

1m-PFOS	**0.0427 (0.0114, 0.0910)**	**0.0304 (0.0006, 0.0772)**	**0.0123 (0.0063, 0.0195)**	**28.7674 (11.1426, 89.4431)**
iso-PFOS	**0.0596 (0.0283, 0.0921)**	**0.0456 (0.0138, 0.0757)**	**0.0141 (0.0063, 0.0227)**	**23.5944 (10.3591, 53.9343)**
3 + 4 + 5m-PFOS	**0.0389 (0.0228, 0.0607)**	**0.0375 (0.0213, 0.0598)**	**0.0014 (0.0005, 0.0025)**	**3.4778 (1.1482, 8.2660)**
∑m2-PFOS	**0.0589 (0.0112, 0.1216)**	0.0376 (-0.0048, 0.0955)	**0.0213 (0.0104, 0.0347)**	**36.1455 (15.4733, 133.5154)**
PFHpS	**0.0622 (0.0223, 0.1167)**	**0.0469 (0.0093, 0.0979)**	**0.0153 (0.0075, 0.0258)**	**24.5588 (11.3552, 61.8882)**
Total PFHxS	**0.0256 (0.0012, 0.0590)**	0.0153 (-0.0080, 0.0474)	**0.0103 (0.0056, 0.0165)**	**40.3700 (13.2346, 257.4503)**
n-PFHxS	0.0222 (-0.0034, 0.0553)	0.0119 (-0.0127, 0.0439)	**0.0103 (0.0058, 0.0170)**	**46.4934 (-194.4324, 310.4169)**
Br-PFHxS	**0.0351 (0.0050, 0.0817)**	0.0278 (-0.0000, 0.0721)	**0.0074 (0.0030, 0.0130)**	**20.9291 (7.6122, 80.3942)**
PFOA	**0.0246 (0.0155, 0.0297)**	**0.0219 (0.0142, 0.0260)**	**0.0027 (0.0009, 0.0058)**	**11.1160 (5.4075, 22.1239)**
PFHpA	**0.0275 (0.0043, 0.0669)**	0.0213 (-0.0007, 0.0602)	**0.0062 (0.0023, 0.0117)**	**22.5513 (7.1117, 116.1289)**
PFHxA	0.0058 (-0.0058, 0.0285)	0.0036 (-0.0078, 0.0250)	**0.0023 (0.0003, 0.0049)**	**38.7493 (-298.9702, 464.0681)**
PFNA	**0.0829 (0.0485, 0.1212)**	**0.0676 (0.0352, 0.1060)**	**0.0152 (0.0071, 0.0247)**	**18.3659 (7.7543, 33.5824)**
PFDA	**0.0793 (0.0464, 0.1199)**	**0.0658 (0.0339, 0.1047)**	**0.0136 (0.0056, 0.0226)**	**17.1127 (6.8686, 31.5690)**
PFUnDA	**0.0624 (0.0304, 0.1078)**	**0.0489 (0.0179, 0.0946)**	**0.0135 (0.0058, 0.0214)**	**21.5822 (8.6131, 43.1914)**
PFDoDA	**0.0608 (0.0187, 0.1187)**	**0.0456 (0.0072, 0.1025)**	**0.0152 (0.0072, 0.0252)**	**25.0432 (11.1630, 62.8005)**
PFTrDA	**0.0464 (0.0144, 0.0955)**	**0.0345 (0.0034, 0.0840)**	**0.0118 (0.0059, 0.0190)**	**25.4874 (10.0320, 75.9639)**
**PFAS[a]->TP->Sarcopenia**				
Total PFOS	**0.0144 (0.0093, 0.0195)**	**0.0144 (0.0093, 0.0195)**	-0.0000 (-0.0005, 0.0004)	-0.0029 (-3.1640, 2.6686)
n-PFOS	**0.0192 (0.0136, 0.0232)**	**0.0192 (0.0137, 0.0233)**	-0.0000 (-0.0005, 0.0005)	-0.0347 (-2.7304, 2.8077)
Br-PFOS	**0.0420 (0.0319, 0.0508)**	**0.0420 (0.0318, 0.0506)**	0.0000 (-0.0005, 0.0007)	0.1069 (-1.1319, 1.6138)
1m-PFOS	**0.0442 (0.0156, 0.0887)**	**0.0439 (0.0152, 0.0884)**	0.0003 (-0.0020, 0.0027)	0.6918 (-5.5059, 6.5370)
iso-PFOS	**0.0603 (0.0302, 0.0909)**	**0.0602 (0.0301, 0.0912)**	0.0001 (-0.0023, 0.0025)	0.1852 (-4.1400, 4.7884)
3 + 4 + 5m-PFOS	**0.0425 (0.0238, 0.0641)**	**0.0425 (0.0238, 0.0641)**	0.0000 (-0.0002, 0.0002)	0.0028 (-0.4669, 0.6114)
∑m2-PFOS	**0.0581 (0.0157, 0.1132)**	**0.0576 (0.0150, 0.1126)**	0.0005 (-0.0022, 0.0043)	0.9028 (-4.4819, 8.5778)
PFHpS	**0.0639 (0.0219, 0.1200)**	**0.0635 (0.0206, 0.1195)**	0.0004 (-0.0025, 0.0036)	0.5602 (-4.7215, 7.8693)

Total PFHxS	**0.0243 (0.0003, 0.0600)**	0.0240 (-0.0000, 0.0596)	0.0003 (-0.0012, 0.0021)	1.4156 (-9.1665, 22.1957)
n-PFHxS	0.0211 (-0.0015, 0.0563)	0.0208 (-0.0023, 0.0564)	0.0004 (-0.0012, 0.0022)	1.7612 (-14.8463, 26.0813)
Br-PFHxS	**0.0299 (0.0031, 0.0716)**	**0.0297 (0.0026, 0.0714)**	0.0002 (-0.0007, 0.0014)	0.5498 (-2.9954, 8.2345)
PFOA	**0.0235 (0.0152, 0.0296)**	**0.0234 (0.0153, 0.0292)**	0.0000 (-0.0003, 0.0005)	0.1861 (-1.3165, 2.2195)
PFHpA	**0.0302 (0.0031, 0.0713)**	**0.0299 (0.0028, 0.0721)**	0.0003 (-0.0015, 0.0021)	1.0514 (-5.6780, 14.3547)
PFHxA	0.0052 (-0.0066, 0.0303)	0.0051 (-0.0067, 0.0302)	0.0001 (-0.0005, 0.0009)	2.3236 (-34.6284, 31.9120)
PFNA	**0.0861 (0.0504, 0.1277)**	**0.0859 (0.0497, 0.1274)**	0.0001 (-0.0023, 0.0028)	0.1492 (-2.5515, 3.1712)
PFDA	**0.0817 (0.0492, 0.1219)**	**0.0815 (0.0493, 0.1214)**	0.0001 (-0.0018, 0.0019)	0.1739 (-2.2442, 2.1930)
PFUnDA	**0.0639 (0.0317, 0.1083)**	**0.0638 (0.0315, 0.1083)**	0.0002 (-0.0010, 0.0017)	0.2631 (-1.8326, 3.0264)
PFDoDA	**0.0637 (0.0212, 0.1254)**	**0.0635 (0.0217, 0.1256)**	0.0002 (-0.0011, 0.0019)	0.2494 (-1.7942, 3.3960)
PFTrDA	**0.0523 (0.0163, 0.1095)**	**0.0522 (0.0161, 0.1093)**	0.0001 (-0.0009, 0.0012)	0.2332 (-2.1360, 3.0021)
**PFAS[a]->ALT->Sarcopenia**				
Total PFOS	**0.0144 (0.0091, 0.0192)**	**0.0145 (0.0091, 0.0194)**	-0.0000 (-0.0008, 0.0004)	-0.0946 (-5.3650, 2.5312)
n-PFOS	**0.0192 (0.0138, 0.0230)**	**0.0192 (0.0139, 0.0230)**	0.0000 (-0.0008, 0.0004)	0.0189 (-3.9412, 2.0061)
Br-PFOS	**0.0421 (0.0324, 0.0506)**	**0.0422 (0.0322, 0.0511)**	-0.0001 (-0.0023, 0.0010)	-0.2440 (-5.3323, 2.4362)
1m-PFOS	**0.0443 (0.0140, 0.0966)**	**0.0440 (0.0139, 0.0970)**	0.0003 (-0.0049, 0.0039)	0.7352 (-14.0541, 10.0202)
iso-PFOS	**0.0603 (0.0328, 0.0931)**	**0.0601 (0.0332, 0.0932)**	0.0002 (-0.0042, 0.0025)	0.2921 (-7.1252, 4.8036)
3 + 4 + 5m-PFOS	**0.0427 (0.0248, 0.0648)**	**0.0427 (0.0250, 0.0648)**	-0.0000 (-0.0008, 0.0004)	-0.0377 (-1.8495, 1.1969)
∑m2-PFOS	**0.0580 (0.0143, 0.1166)**	**0.0577 (0.0134, 0.1170)**	0.0004 (-0.0060, 0.0044)	0.6653 (-12.0006, 11.1341)
PFHpS	**0.0641 (0.0229, 0.1216)**	**0.0637 (0.0223, 0.1209)**	0.0004 (-0.0051, 0.0042)	0.5982 (-9.7140, 7.9651)
Total PFHxS	0.0245 (-0.0016, 0.0576)	0.0239 (-0.0019, 0.0566)	0.0006 (-0.0033, 0.0036)	2.4025 (-31.5569, 29.7912)
n-PFHxS	0.0213 (-0.0022, 0.0535)	0.0207 (-0.0025, 0.0535)	0.0006 (-0.0033, 0.0037)	2.7736 (-35.1459, 58.2125)
Br-PFHxS	**0.0299 (0.0019, 0.0725)**	**0.0295 (0.0020, 0.0717)**	0.0003 (-0.0015, 0.0020)	1.1440 (-12.8844, 10.0953)
PFOA	**0.0235 (0.0150, 0.0289)**	**0.0234 (0.0152, 0.0293)**	0.0001 (-0.0014, 0.0009)	0.2847 (-6.2812, 3.8776)
PFHpA	**0.0303 (0.0037, 0.0764)**	**0.0300 (0.0033, 0.0770)**	0.0003 (-0.0029, 0.0023)	0.8983 (-14.0273, 14.6204)
PFHxA	0.0053 (-0.0066, 0.0286)	0.0052 (-0.0066, 0.0286)	0.0001 (-0.0005, 0.0006)	1.1022 (-22.1896, 30.5182)
PFNA	**0.0862 (0.0519, 0.1266)**	**0.0859 (0.0522, 0.1261)**	0.0003 (-0.0044, 0.0027)	0.3489 (-5.3735, 3.3193)

PFDA	**0.0815 (0.0464, 0.1166)**	**0.0814 (0.0461, 0.1174)**	0.0001 (-0.0019, 0.0011)	0.1495 (-2.3692, 1.4974)
PFUnDA	**0.0638 (0.0322, 0.1078)**	**0.0637 (0.0322, 0.1078)**	0.0001 (-0.0013, 0.0008)	0.1002 (-1.9634, 1.3254)
PFDoDA	**0.0637 (0.0205, 0.1355)**	**0.0636 (0.0206, 0.1353)**	0.0001 (-0.0018, 0.0013)	0.1837 (-2.9513, 2.2500)
PFTrDA	**0.0527 (0.0189, 0.1036)**	**0.0525 (0.0198, 0.1035)**	0.0002 (-0.0016, 0.0013)	0.3776 (-3.2816, 2.7829)
**PFAS[a]->AST->Sarcopenia**				
Total PFOS	**0.0146 (0.0095, 0.0191)**	**0.0140 (0.0091, 0.0181)**	0.0007 (-0.0002, 0.0019)	4.4614 (-1.0410, 11.2932)
n-PFOS	**0.0193 (0.0140, 0.0233)**	**0.0185 (0.0135, 0.0225)**	0.0008 (-0.0001, 0.0022)	4.0157 (-0.4584, 10.6201)
Br-PFOS	**0.0415 (0.0314, 0.0496)**	**0.0400 (0.0298, 0.0486)**	0.0015 (-0.0002, 0.0036)	3.5337 (-0.3749, 9.2746)
1m-PFOS	**0.0433 (0.0120, 0.0858)**	**0.0381 (0.0071, 0.0793)**	**0.0052 (0.0005, 0.0108)**	**12.0120 (1.0249, 42.7807)**
iso-PFOS	**0.0600 (0.0307, 0.0903)**	**0.0557 (0.0259, 0.0867)**	0.0044 (-0.0000, 0.0096)	7.2676 (-0.0325, 19.6172)
3 + 4 + 5m-PFOS	**0.0411 (0.0244, 0.0633)**	**0.0405 (0.0238, 0.0624)**	**0.0006 (0.0000, 0.0016)**	**1.5453 (0.0177, 4.6803)**
∑m2-PFOS	**0.0566 (0.0131, 0.1168)**	**0.0478 (0.0077, 0.1076)**	**0.0087 (0.0007, 0.0180)**	**15.4442 (1.2251, 49.4726)**
PFHpS	**0.0623 (0.0214, 0.1184)**	**0.0560 (0.0156, 0.1129)**	**0.0063 (0.0007, 0.0131)**	**10.1122 (0.9182, 32.7335)**
Total PFHxS	0.0238 (-0.0001, 0.0574)	0.0184 (-0.0056, 0.0512)	0.0054 (0.0009, 0.0105)	22.5191 (-31.7948, 156.4681)
n-PFHxS	0.0207 (-0.0018, 0.0542)	0.0153 (-0.0070, 0.0494)	0.0053 (0.0009, 0.0106)	25.7293 (-85.8112, 186.1468)
Br-PFHxS	**0.0307 (0.0043, 0.0761)**	**0.0262 (0.0008, 0.0689)**	**0.0045 (0.0007, 0.0091)**	**14.6510 (0.4688, 57.1361)**
PFOA	**0.0239 (0.0158, 0.0294)**	**0.0228 (0.0151, 0.0279)**	0.0011 (-0.0001, 0.0030)	4.5988 (-0.3586, 11.2988)
PFHpA	**0.0294 (0.0034, 0.0728)**	**0.0256 (0.0006, 0.0689)**	**0.0038 (0.0004, 0.0078)**	**12.8957 (0.8320, 66.1948)**
PFHxA	0.0051 (-0.0062, 0.0278)	0.0033 (-0.0076, 0.0256)	0.0018 (0.0003, 0.0039)	35.0328 (-359.3937, 324.8599)
PFNA	**0.0836 (0.0484, 0.1228)**	**0.0793 (0.0437, 0.1186)**	0.0043 (-0.0001, 0.0100)	5.1825 (-0.1518, 12.8272)
PFDA	**0.0788 (0.0481, 0.1180)**	**0.0758 (0.0451, 0.1154)**	**0.0030 (0.0001, 0.0069)**	**3.8380 (0.1717, 9.5815)**
PFUnDA	**0.0610 (0.0296, 0.1027)**	**0.0583 (0.0272, 0.0999)**	**0.0027 (0.0002, 0.0063)**	**4.4426 (0.3334, 12.4297)**
PFDoDA	**0.0606 (0.0178, 0.1218)**	**0.0571 (0.0159, 0.1189)**	**0.0035 (0.0001, 0.0082)**	**5.8373 (0.1563, 18.0846)**
PFTrDA	**0.0490 (0.0150, 0.1018)**	**0.0468 (0.0130, 0.0998)**	**0.0022 (0.0000, 0.0057)**	**4.4945 (0.0101, 18.0626)**
**PFAS[a]->GGT->Sarcopenia**				
Total PFOS	**0.0144 (0.0095, 0.0192)**	**0.0139 (0.0091, 0.0184)**	**0.0006 (0.0001, 0.0014)**	**3.9664 (0.4142, 8.2786)**
n-PFOS	**0.0192 (0.0138, 0.0230)**	**0.0186 (0.0134, 0.0224)**	**0.0006 (0.0000, 0.0016)**	**3.3130 (0.2516, 7.7165)**

Br-PFOS	**0.0415 (0.0315, 0.0499)**	**0.0399 (0.0298, 0.0481)**	**0.0015 (0.0000, 0.0033)**	**3.6580 (0.0104, 8.2321)**
1m-PFOS	**0.0429 (0.0115, 0.0887)**	**0.0388 (0.0096, 0.0826)**	**0.0040 (0.0006, 0.0080)**	**9.4328 (1.2092, 31.2488)**
iso-PFOS	**0.0603 (0.0323, 0.0888)**	**0.0566 (0.0294, 0.0856)**	**0.0037 (0.0002, 0.0073)**	**6.0815 (0.2881, 13.4440)**
3 + 4 + 5m-PFOS	**0.0410 (0.0241, 0.0650)**	**0.0403 (0.0236, 0.0643)**	**0.0008 (0.0000, 0.0017)**	**1.9342 (0.0859, 4.8145)**
∑m2-PFOS	**0.0585 (0.0162, 0.1176)**	**0.0522 (0.0128, 0.1103)**	**0.0063 (0.0008, 0.0121)**	**10.8483 (1.1245, 28.5662)**
PFHpS	**0.0624 (0.0191, 0.1166)**	**0.0569 (0.0157, 0.1102)**	**0.0055 (0.0010, 0.0105)**	**8.8586 (1.7902, 24.0128)**
Total PFHxS	**0.0244 (0.0019, 0.0642)**	0.0202 (-0.0024, 0.0591)	**0.0041 (0.0007, 0.0081)**	16.9317 (-1.9174, 77.4402)
n-PFHxS	0.0210 (-0.0012, 0.0540)	0.0170 (-0.0050, 0.0503)	**0.0040 (0.0005, 0.0079)**	19.0884 (-76.3420, 156.7999)
Br-PFHxS	0.0304 (0.0019, 0.0781)	**0.0281 (0.0013, 0.0750)**	0.0022 (-0.0000, 0.0053)	7.3841 (-0.8078, 36.6123)
PFOA	**0.0234 (0.0150, 0.0291)**	**0.0225 (0.0147, 0.0277)**	**0.0009 (0.0001, 0.0019)**	**3.7931 (0.3565, 7.6926)**
PFHpA	**0.0307 (0.0054, 0.0746)**	**0.0274 (0.0032, 0.0708)**	**0.0033 (0.0008, 0.0069)**	**10.6135 (2.1121, 44.7030)**
PFHxA	0.0059 (-0.0059, 0.0317)	0.0058 (-0.0059, 0.0312)	0.0001 (-0.0013, 0.0014)	1.4834 (-72.2316, 65.2324)
PFNA	**0.0859 (0.0508, 0.1293)**	**0.0811 (0.0466, 0.1248)**	**0.0048 (0.0001, 0.0087)**	**5.5562 (0.1129, 12.0413)**
PFDA	**0.0812 (0.0491, 0.1193)**	**0.0787 (0.0470, 0.1173)**	**0.0026 (0.0002, 0.0053)**	**3.1406 (0.1960, 7.4113)**
PFUnDA	**0.0619 (0.0314, 0.1057)**	**0.0598 (0.0288, 0.1024)**	0.0021 (-0.0001, 0.0050)	3.3412 (-0.3443, 10.0248)
PFDoDA	**0.0598 (0.0201, 0.1214)**	**0.0578 (0.0190, 0.1204)**	0.0020 (-0.0005, 0.0052)	3.4243 (-0.9669, 12.7761)
PFTrDA	**0.0497 (0.0157, 0.0951)**	**0.0480 (0.0143, 0.0937)**	0.0017 (-0.0005, 0.0045)	3.4057 (-1.1923, 12.1838)

Abbreviations: ALB, albumin; GLB, globin; TP, total protein; ALT, Alanine aminotransferase; AST, Aspartate Aminotransferase; GGT, Gamma-Glutamyl Transferase.

Model was adjusted for age, sex, education, alcohol drinking, smoking, family income, exercise, BMI, district.

Bolding indicates that associations were statistically significant ($P < 0{\cdot}05$).

[a] The variables were natural-log transformed.

[b] The complete mediation.

Table S9. The mediation of markers of liver function in the association between ASMI and PFAS in serum (N=1,261).

Pathways	Total effect	Direct effect	Indirect effect	Prop mediated
**PFAS[a]->ALB->ASMI**				
Total PFOS	**-0.7817 (-0.8470, -0.7232)**	**-0.3519 (-0.4029, -0.3044)**	**-0.4298 (-0.4986, -0.3629)**	**45.0133 (39.2073, 51.5794)**
n-PFOS	**-0.7004 (-0.7601, -0.6485)**	**-0.3134 (-0.3613, -0.2712)**	**-0.3869 (-0.4537, -0.3279)**	**44.7541 (38.3453, 51.2160)**
Br-PFOS	**-0.3957 (-0.4554, -0.3402)**	**-0.2482 (-0.2957, -0.2083)**	**-0.1475 (-0.1885, -0.1107)**	**62.7283 (55.7900, 70.3387)**
1m-PFOS	**-0.5475 (-0.5984, -0.4929)**	**-0.3279 (-0.3746, -0.2862)**	**-0.2196 (-0.2781, -0.1595)**	**59.8956 (51.7837, 68.6271)**
iso-PFOS	**-0.6672 (-0.7301, -0.6099)**	**-0.3441 (-0.3938, -0.3013)**	**-0.3231 (-0.3865, -0.2693)**	**51.5762 (44.8004, 58.3970)**
3 + 4 + 5m-PFOS	**-0.0980 (-0.1188, -0.0769)**	**-0.0617 (-0.0770, -0.0470)**	**-0.0362 (-0.0519, -0.0199)**	**63.0169 (52.0778, 77.0008)**
∑m2-PFOS	**-0.5964 (-0.6647, -0.5301)**	**-0.3587 (-0.4086, -0.3137)**	**-0.2377 (-0.2928, -0.1801)**	**60.1504 (53.6484, 67.7272)**
PFHpS	**-0.6131 (-0.6828, -0.5504)**	**-0.3760 (-0.4281, -0.3284)**	**-0.2372 (-0.3051, -0.1677)**	**61.3220 (53.2421, 70.8068)**
Total PFHxS	**-0.5686 (-0.6341, -0.5100)**	**-0.3714 (-0.4204, -0.3254)**	**-0.1971 (-0.2640, -0.1351)**	**65.3280 (56.5442, 74.5794)**
n-PFHxS	**-0.5587 (-0.6177, -0.4960)**	**-0.3629 (-0.4121, -0.3207)**	**-0.1959 (-0.2620, -0.1337)**	**64.9433 (56.0727, 74.4022)**
Br-PFHxS	**-0.2824 (-0.3362, -0.2285)**	**-0.2034 (-0.2371, -0.1708)**	**-0.0790 (-0.1265, -0.0324)**	**72.0208 (60.8656, 87.1923)**
PFOA	**-0.5787 (-0.6792, -0.4935)**	**-0.3515 (-0.4139, -0.2920)**	**-0.2272 (-0.3035, -0.1636)**	**60.7418 (52.4721, 69.2389)**
PFHpA	**-0.2401 (-0.2892, -0.1972)**	**-0.1871 (-0.2183, -0.1560)**	**-0.0531 (-0.0890, -0.0193)**	**77.9032 (67.1856, 91.0790)**
PFHxA	**-0.0949 (-0.1566, -0.0464)**	**-0.0502 (-0.0784, -0.0215)**	**-0.0447 (-0.0936, -0.0045)**	**52.9196 (29.2472, 90.1930)**
PFNA	**-0.8188 (-0.8958, -0.7477)**	**-0.4089 (-0.4611, -0.3613)**	**-0.4099 (-0.4889, -0.3342)**	**49.9376 (43.8438, 56.6506)**
PFDA	**-0.6780 (-0.7356, -0.6169)**	**-0.3254 (-0.3706, -0.2850)**	**-0.3526 (-0.4148, -0.2899)**	**47.9958 (42.0550, 54.8179)**
PFUnDA	**-0.6169 (-0.6777, -0.5631)**	**-0.2980 (-0.3429, -0.2593)**	**-0.3190 (-0.3821, -0.2571)**	**48.2955 (41.5342, 55.7442)**
PFDoDA	**-0.4810 (-0.5358, -0.4306)**	**-0.2847 (-0.3245, -0.2476)**	**-0.1963 (-0.2508, -0.1426)**	**59.1877 (51.5077, 68.3550)**
PFTrDA	**-0.4374 (-0.5053, -0.3822)**	**-0.2478 (-0.2874, -0.2148)**	**-0.1896 (-0.2448, -0.1410)**	**56.6509 (49.2426, 64.9865)**
**PFAS[a]->GLB->ASMI**				
Total PFOS	**-0.7817 (-0.8497, -0.7172)**	**-0.0791 (-0.1076, -0.0535)**	**-0.7026 (-0.7646, -0.6377)**	**10.1203 (6.9274, 13.8014)**
n-PFOS	**-0.7004 (-0.7563, -0.6436)**	**-0.0712 (-0.0954, -0.0456)**	**-0.6292 (-0.6856, -0.5715)**	**10.1653 (6.5531, 13.6829)**
Br-PFOS	**-0.3957 (-0.4612, -0.3398)**	**-0.0597 (-0.0813, -0.0399)**	**-0.3361 (-0.3929, -0.2851)**	**15.0823 (10.8284, 20.0476)**

1m-PFOS	**-0.5475 (-0.6025, -0.4911)**	**-0.0735 (-0.0963, -0.0513)**	**-0.4740 (-0.5300, -0.4155)**	**13.4235 (9.4963, 17.4832)**
iso-PFOS	**-0.6672 (-0.7299, -0.6070)**	**-0.0786 (-0.1015, -0.0534)**	**-0.5886 (-0.6488, -0.5326)**	**11.7806 (8.2346, 15.0675)**
3 + 4 + 5m-PFOS	**-0.0980 (-0.1188, -0.0769)**	**-0.0150 (-0.0235, -0.0071)**	**-0.0830 (-0.1029, -0.0624)**	**15.2599 (7.4799, 23.8072)**
∑m2-PFOS	**-0.5964 (-0.6648, -0.5300)**	**-0.0856 (-0.1141, -0.0616)**	**-0.5108 (-0.5745, -0.4496)**	**14.3593 (10.4256, 18.6017)**
PFHpS	**-0.6131 (-0.6841, -0.5501)**	**-0.0782 (-0.1045, -0.0549)**	**-0.5349 (-0.6014, -0.4738)**	**12.7588 (9.2978, 16.7121)**
Total PFHxS	**-0.5686 (-0.6333, -0.5119)**	**-0.0795 (-0.1066, -0.0559)**	**-0.4891 (-0.5451, -0.4371)**	**13.9851 (10.0524, 18.2048)**
n-PFHxS	**-0.5587 (-0.6196, -0.5031)**	**-0.0789 (-0.1056, -0.0566)**	**-0.4799 (-0.5338, -0.4273)**	**14.1124 (10.1906, 18.3883)**
Br-PFHxS	**-0.2824 (-0.3395, -0.2304)**	**-0.0448 (-0.0635, -0.0251)**	**-0.2376 (-0.2862, -0.1906)**	**15.8693 (9.7531, 22.1119)**
PFOA	**-0.5787 (-0.6746, -0.4936)**	**-0.0907 (-0.1206, -0.0652)**	**-0.4880 (-0.5771, -0.4092)**	**15.6759 (11.2576, 20.8930)**
PFHpA	**-0.2401 (-0.2884, -0.1957)**	**-0.0369 (-0.0567, -0.0199)**	**-0.2033 (-0.2458, -0.1629)**	**15.3625 (8.8730, 22.3947)**
PFHxA	**-0.0949 (-0.1512, -0.0446)**	**-0.0190 (-0.0342, -0.0035)**	**-0.0759 (-0.1314, -0.0276)**	**19.9950 (4.8045, 45.6913)**
PFNA	**-0.8188 (-0.9016, -0.7449)**	**-0.0957 (-0.1257, -0.0652)**	**-0.7231 (-0.8066, -0.6524)**	**11.6827 (7.9831, 15.3664)**
PFDA	**-0.6780 (-0.7390, -0.6176)**	**-0.0812 (-0.1059, -0.0568)**	**-0.5968 (-0.6563, -0.5382)**	**11.9725 (8.4450, 15.4569)**
PFUnDA	**-0.6169 (-0.6819, -0.5586)**	**-0.0807 (-0.1054, -0.0561)**	**-0.5362 (-0.5980, -0.4751)**	**13.0823 (9.2938, 17.1588)**
PFDoDA	**-0.4810 (-0.5382, -0.4307)**	**-0.0686 (-0.0911, -0.0495)**	**-0.4124 (-0.4655, -0.3635)**	**14.2684 (10.4126, 18.5044)**
PFTrDA	**-0.4374 (-0.4978, -0.3773)**	**-0.0714 (-0.0945, -0.0512)**	**-0.3660 (-0.4275, -0.3068)**	**16.3308 (11.7559, 21.4256)**
**PFAS[a]->TP->ASMI**				
Total PFOS	**-0.7817 (-0.8502, -0.7189)**	**-0.0050 (-0.0124, 0.0002)**	**-0.7767 (-0.8425, -0.7148)**	0.6434 (-0.0260, 1.5660)
n-PFOS	**-0.7004 (-0.7589, -0.6443)**	**-0.0046 (-0.0117, 0.0003)**	**-0.6958 (-0.7525, -0.6406)**	0.6501 (-0.0410, 1.6429)
Br-PFOS	**-0.3957 (-0.4610, -0.3400)**	**-0.0032 (-0.0095, 0.0005)**	**-0.3926 (-0.4585, -0.3388)**	0.7984 (-0.1318, 2.2423)
1m-PFOS	**-0.5475 (-0.6054, -0.4929)**	**-0.0050 (-0.0123, -0.0001)**	**-0.5425 (-0.5993, -0.4899)**	**0.9169 (0.0265, 2.2086)**
iso-PFOS	**-0.6672 (-0.7300, -0.6045)**	**-0.0050 (-0.0125, -0.0000)**	**-0.6621 (-0.7236, -0.5998)**	**0.7500 (0.0037, 1.8401)**
3 + 4 + 5m-PFOS	**-0.0980 (-0.1191, -0.0764)**	**-0.0001 (-0.0024, 0.0022)**	**-0.0978 (-0.1190, -0.0767)**	0.1445 (-2.3885, 2.4405)
∑m2-PFOS	**-0.5964 (-0.6647, -0.5285)**	**-0.0057 (-0.0139, -0.0002)**	**-0.5907 (-0.6553, -0.5249)**	**0.9498 (0.0404, 2.2492)**

PFHpS	**-0.6131 (-0.6759, -0.5552)**	**-0.0055 (-0.0128, -0.0004)**	**-0.6077 (-0.6688, -0.5493)**	**0.8917 (0.0613, 2.0708)**
Total PFHxS	**-0.5686 (-0.6362, -0.5045)**	**-0.0055 (-0.0133, -0.0003)**	**-0.5631 (-0.6320, -0.5005)**	**0.9610 (0.0472, 2.3239)**
n-PFHxS	**-0.5587 (-0.6197, -0.4998)**	**-0.0057 (-0.0137, -0.0004)**	**-0.5530 (-0.6146, -0.4947)**	**1.0225 (0.0658, 2.4058)**
Br-PFHxS	**-0.2824 (-0.3353, -0.2305)**	**-0.0018 (-0.0071, 0.0027)**	**-0.2806 (-0.3320, -0.2314)**	0.6508 (-1.0194, 2.4947)
PFOA	**-0.5787 (-0.6831, -0.4924)**	**-0.0056 (-0.0146, 0.0006)**	**-0.5732 (-0.6776, -0.4860)**	0.9632 (-0.1050, 2.4782)
PFHpA	**-0.2401 (-0.2831, -0.1978)**	**-0.0047 (-0.0108, -0.0003)**	**-0.2354 (-0.2787, -0.1935)**	**1.9613 (0.1416, 4.4904)**
PFHxA	**-0.0949 (-0.1520, -0.0473)**	**-0.0022 (-0.0070, 0.0017)**	**-0.0927 (-0.1484, -0.0459)**	2.3195 (-1.7951, 8.6982)
PFNA	**-0.8188 (-0.8937, -0.7420)**	**-0.0061 (-0.0146, -0.0003)**	**-0.8126 (-0.8885, -0.7371)**	**0.7468 (0.0355, 1.7458)**
PFDA	**-0.6780 (-0.7380, -0.6200)**	**-0.0047 (-0.0115, -0.0001)**	**-0.6733 (-0.7338, -0.6157)**	**0.6889 (0.0217, 1.6658)**
PFUnDA	**-0.6169 (-0.6768, -0.5615)**	**-0.0040 (-0.0104, 0.0002)**	**-0.6130 (-0.6708, -0.5605)**	0.6459 (-0.0302, 1.7215)
PFDoDA	**-0.4810 (-0.5346, -0.4301)**	**-0.0023 (-0.0084, 0.0031)**	**-0.4787 (-0.5301, -0.4280)**	0.4836 (-0.6573, 1.7110)
PFTrDA	**-0.4374 (-0.5000, -0.3738)**	**-0.0026 (-0.0088, 0.0028)**	**-0.4348 (-0.4981, -0.3718)**	0.5935 (-0.6517, 1.9655)
**PFAS[a]->ALT->ASMI**				
Total PFOS	**-0.7823 (-0.8491, -0.7200)**	**-0.7828 (-0.8486, -0.7204)**	0.0005 (-0.0043, 0.0057)	-0.0679 (-0.7444, 0.5422)
n-PFOS	**-0.7009 (-0.7619, -0.6468)**	**-0.7004 (-0.7625, -0.6470)**	-0.0005 (-0.0043, 0.0031)	0.0697 (-0.4446, 0.5941)
Br-PFOS	**-0.3964 (-0.4600, -0.3417)**	**-0.3961 (-0.4607, -0.3417)**	-0.0002 (-0.0056, 0.0048)	0.0572 (-1.1656, 1.4213)
1m-PFOS	**-0.5483 (-0.6029, -0.4940)**	**-0.5524 (-0.6071, -0.4984)**	0.0040 (-0.0038, 0.0130)	-0.7353 (-2.3706, 0.6969)
iso-PFOS	**-0.6679 (-0.7318, -0.6101)**	**-0.6686 (-0.7317, -0.6105)**	0.0008 (-0.0042, 0.0066)	-0.1160 (-1.0124, 0.6283)
3 + 4 + 5m-PFOS	**-0.0987 (-0.1204, -0.0771)**	**-0.0980 (-0.1190, -0.0756)**	-0.0007 (-0.0029, 0.0005)	0.6923 (-0.5619, 2.9915)
∑m2-PFOS	**-0.5971 (-0.6634, -0.5343)**	**-0.5982 (-0.6631, -0.5363)**	0.0011 (-0.0062, 0.0085)	-0.1849 (-1.4299, 1.0270)
PFHpS	**-0.6134 (-0.6812, -0.5517)**	**-0.6169 (-0.6856, -0.5555)**	0.0035 (-0.0041, 0.0131)	-0.5689 (-2.1241, 0.6801)
Total PFHxS	**-0.5695 (-0.6329, -0.5089)**	**-0.5712 (-0.6354, -0.5111)**	0.0016 (-0.0068, 0.0107)	-0.2889 (-1.8530, 1.2139)
n-PFHxS	**-0.5597 (-0.6194, -0.5013)**	**-0.5615 (-0.6218, -0.5007)**	0.0018 (-0.0063, 0.0109)	-0.3255 (-1.9515, 1.1073)
Br-PFHxS	**-0.2826 (-0.3362, -0.2326)**	**-0.2813 (-0.3343, -0.2311)**	-0.0013 (-0.0059, 0.0018)	0.4736 (-0.6200, 2.0246)
PFOA	**-0.5799 (-0.6757, -0.4950)**	**-0.5799 (-0.6755, -0.4933)**	-0.0000 (-0.0092, 0.0093)	0.0009 (-1.7475, 1.6448)
PFHpA	**-0.2408 (-0.2866, -0.1984)**	**-0.2396 (-0.2838, -0.1976)**	-0.0012 (-0.0065, 0.0032)	0.4905 (-1.3506, 2.5609)

PFHxA	**-0.0952 (-0.1521, -0.0426)**	**-0.0947 (-0.1509, -0.0423)**	-0.0005 (-0.0034, 0.0014)	0.4878 (-1.7535, 3.8860)
PFNA	**-0.8197 (-0.8980, -0.7447)**	**-0.8197 (-0.8981, -0.7462)**	0.0001 (-0.0060, 0.0068)	-0.0076 (-0.8212, 0.7150)
PFDA	**-0.6783 (-0.7412, -0.6234)**	**-0.6774 (-0.7407, -0.6240)**	-0.0009 (-0.0045, 0.0019)	0.1321 (-0.2735, 0.6702)
PFUnDA	**-0.6172 (-0.6728, -0.5591)**	**-0.6167 (-0.6722, -0.5588)**	-0.0005 (-0.0040, 0.0022)	0.0788 (-0.3485, 0.6435)
PFDoDA	**-0.4818 (-0.5389, -0.4305)**	**-0.4811 (-0.5381, -0.4303)**	-0.0007 (-0.0040, 0.0019)	0.1427 (-0.4030, 0.8378)
PFTrDA	**-0.4383 (-0.5005, -0.3789)**	**-0.4374 (-0.5007, -0.3785)**	-0.0010 (-0.0045, 0.0012)	0.2179 (-0.2836, 1.0238)
**PFAS[a]->AST->ASMI**				
Total PFOS	**-0.7823 (-0.8478, -0.7204)**	**-0.7750 (-0.8390, -0.7127)**	-0.0073 (-0.0176, 0.0013)	0.9305 (-0.1559, 2.1840)
n-PFOS	**-0.7009 (-0.7565, -0.6444)**	**-0.6937 (-0.7501, -0.6366)**	-0.0072 (-0.0156, 0.0001)	1.0338 (-0.0185, 2.2075)
Br-PFOS	**-0.3964 (-0.4577, -0.3415)**	**-0.3866 (-0.4477, -0.3308)**	**-0.0098 (-0.0178, -0.0031)**	**2.4724 (0.7806, 4.5147)**
1m-PFOS	**-0.5483 (-0.6057, -0.4888)**	**-0.5400 (-0.5978, -0.4822)**	**-0.0084 (-0.0181, -0.0001)**	**1.5249 (0.0228, 3.2230)**
iso-PFOS	**-0.6679 (-0.7289, -0.6117)**	**-0.6603 (-0.7209, -0.6042)**	-0.0075 (-0.0175, 0.0010)	1.1273 (-0.1447, 2.6130)
3 + 4 + 5m-PFOS	**-0.0987 (-0.1215, -0.0770)**	**-0.0951 (-0.1167, -0.0737)**	**-0.0036 (-0.0074, -0.0004)**	**3.6215 (0.4845, 7.8390)**
∑m2-PFOS	**-0.5971 (-0.6649, -0.5318)**	**-0.5884 (-0.6548, -0.5237)**	-0.0087 (-0.0202, 0.0017)	1.4617 (-0.2983, 3.3429)
PFHpS	**-0.6134 (-0.6805, -0.5483)**	**-0.6051 (-0.6730, -0.5443)**	-0.0082 (-0.0183, 0.0010)	1.3424 (-0.1497, 3.0105)
Total PFHxS	**-0.5695 (-0.6335, -0.5129)**	**-0.5573 (-0.6217, -0.4995)**	**-0.0123 (-0.0234, -0.0020)**	**2.1521 (0.3562, 4.2401)**
n-PFHxS	**-0.5597 (-0.6224, -0.4981)**	**-0.5478 (-0.6141, -0.4857)**	**-0.0119 (-0.0250, -0.0013)**	**2.1226 (0.2315, 4.3988)**
Br-PFHxS	**-0.2826 (-0.3374, -0.2305)**	**-0.2716 (-0.3258, -0.2182)**	**-0.0111 (-0.0199, -0.0041)**	**3.9214 (1.4097, 7.2190)**
PFOA	**-0.5799 (-0.6774, -0.4937)**	**-0.5637 (-0.6600, -0.4779)**	**-0.0162 (-0.0294, -0.0059)**	**2.7968 (1.0114, 5.0154)**
PFHpA	**-0.2408 (-0.2868, -0.1977)**	**-0.2308 (-0.2771, -0.1878)**	**-0.0100 (-0.0178, -0.0036)**	**4.1675 (1.5290, 7.3074)**
PFHxA	**-0.0952 (-0.1499, -0.0422)**	**-0.0874 (-0.1427, -0.0375)**	**-0.0078 (-0.0146, -0.0025)**	**8.1819 (2.5801, 18.5990)**
PFNA	**-0.8197 (-0.8997, -0.7527)**	**-0.8093 (-0.8878, -0.7438)**	**-0.0103 (-0.0203, -0.0013)**	**1.2613 (0.1680, 2.4837)**
PFDA	**-0.6783 (-0.7383, -0.6217)**	**-0.6693 (-0.7278, -0.6121)**	**-0.0091 (-0.0169, -0.0027)**	**1.3354 (0.4062, 2.4995)**
PFUnDA	**-0.6172 (-0.6755, -0.5612)**	**-0.6086 (-0.6667, -0.5521)**	**-0.0086 (-0.0168, -0.0027)**	**1.3965 (0.4275, 2.7992)**
PFDoDA	**-0.4818 (-0.5335, -0.4287)**	**-0.4738 (-0.5246, -0.4217)**	**-0.0081 (-0.0160, -0.0020)**	**1.6721 (0.4329, 3.3163)**
PFTrDA	**-0.4383 (-0.5036, -0.3822)**	**-0.4316 (-0.4989, -0.3766)**	**-0.0067 (-0.0144, -0.0003)**	**1.5362 (0.0735, 3.3654)**

**PFAS[a]->GGT->ASMI**				
Total PFOS	**-0.7823 (-0.8540, -0.7182)**	**-0.7766 (-0.8496, -0.7136)**	-0.0057 (-0.0122, 0.0005)	0.7274 (-0.0598, 1.5313)
n-PFOS	**-0.7009 (-0.7643, -0.6482)**	**-0.6950 (-0.7586, -0.6412)**	**-0.0059 (-0.0119, -0.0009)**	**0.8448 (0.1268, 1.7483)**
Br-PFOS	**-0.3964 (-0.4606, -0.3412)**	**-0.3882 (-0.4524, -0.3324)**	**-0.0082 (-0.0148, -0.0028)**	**2.0712 (0.6962, 3.7402)**
1m-PFOS	**-0.5483 (-0.5983, -0.4956)**	**-0.5411 (-0.5903, -0.4889)**	**-0.0073 (-0.0147, -0.0021)**	**1.3240 (0.3914, 2.6707)**
iso-PFOS	**-0.6679 (-0.7339, -0.6070)**	**-0.6619 (-0.7280, -0.5998)**	-0.0059 (-0.0122, 0.0001)	0.8898 (-0.0185, 1.8404)
3 + 4 + 5m-PFOS	**-0.0987 (-0.1217, -0.0776)**	**-0.0945 (-0.1169, -0.0739)**	**-0.0042 (-0.0072, -0.0017)**	**4.2221 (1.7817, 7.3499)**
∑m2-PFOS	**-0.5971 (-0.6659, -0.5362)**	**-0.5893 (-0.6588, -0.5277)**	**-0.0078 (-0.0158, -0.0003)**	**1.3057 (0.0463, 2.6753)**
PFHpS	**-0.6134 (-0.6883, -0.5480)**	**-0.6065 (-0.6805, -0.5390)**	**-0.0069 (-0.0142, -0.0005)**	**1.1219 (0.0823, 2.3612)**
Total PFHxS	**-0.5695 (-0.6380, -0.5047)**	**-0.5598 (-0.6294, -0.4953)**	**-0.0097 (-0.0179, -0.0031)**	**1.7041 (0.5393, 3.1565)**
n-PFHxS	**-0.5597 (-0.6241, -0.4957)**	**-0.5502 (-0.6170, -0.4883)**	**-0.0095 (-0.0171, -0.0028)**	**1.6888 (0.4857, 3.0921)**
Br-PFHxS	**-0.2826 (-0.3393, -0.2310)**	**-0.2755 (-0.3319, -0.2228)**	**-0.0071 (-0.0133, -0.0021)**	**2.5296 (0.7765, 4.8521)**
PFOA	**-0.5799 (-0.6802, -0.4950)**	**-0.5683 (-0.6692, -0.4825)**	**-0.0116 (-0.0219, -0.0042)**	**2.0028 (0.6989, 3.8788)**
PFHpA	**-0.2408 (-0.2893, -0.2012)**	**-0.2317 (-0.2804, -0.1910)**	**-0.0091 (-0.0156, -0.0036)**	**3.7901 (1.4537, 6.5853)**
PFHxA	**-0.0952 (-0.1544, -0.0416)**	**-0.0924 (-0.1514, -0.0398)**	-0.0028 (-0.0073, 0.0017)	2.9771 (-2.0558, 10.0650)
PFNA	**-0.8197 (-0.9000, -0.7496)**	**-0.8136 (-0.8945, -0.7426)**	-0.0061 (-0.0157, 0.0030)	0.7409 (-0.3721, 1.9076)
PFDA	**-0.6783 (-0.7399, -0.6183)**	**-0.6712 (-0.7305, -0.6115)**	**-0.0072 (-0.0140, -0.0022)**	**1.0585 (0.3300, 2.1096)**
PFUnDA	**-0.6172 (-0.6803, -0.5582)**	**-0.6099 (-0.6739, -0.5498)**	**-0.0073 (-0.0131, -0.0021)**	**1.1796 (0.3533, 2.1092)**
PFDoDA	**-0.4818 (-0.5391, -0.4284)**	**-0.4753 (-0.5335, -0.4208)**	**-0.0065 (-0.0121, -0.0013)**	**1.3480 (0.2806, 2.5234)**
PFTrDA	**-0.4383 (-0.5023, -0.3816)**	**-0.4317 (-0.4950, -0.3750)**	**-0.0067 (-0.0129, -0.0012)**	**1.5192 (0.2665, 2.9904)**

Abbreviations: ALB, albumin; GLB, globin; TP, total protein; ALT, Alanine aminotransferase; AST, Aspartate Aminotransferase; GGT, Gamma-Glutamyl Transferase.

Model was adjusted for age, sex, education, alcohol drinking, smoking, family income, exercise, BMI, district.

Bolding indicates that associations were statistically significant (*P* < 0·05).

[a] The variables were natural-log transformed.

Table S10. The mediation of markers of liver function in the association between GripI and PFAS in serum (N=1,261).

Pathways	Total effect	Direct effect	Indirect effect	Prop mediated
**PFAS[a]->ALB->GripI**				
Total PFOS	-0.0794 (-0.5201, 0.2484)	-0.2416 (-0.7654, 0.1393)	0.1622 (0.0408, 0.2967)	-204.3347 (-1215.5354, 827.2590)
n-PFOS	0.1437 (-0.2565, 0.4358)	0.0692 (-0.4286, 0.4030)	0.0744 (-0.0256, 0.1929)	51.8054 (-528.9769, 535.2744)
Br-PFOS	-0.5127 (-0.7608, -0.3262)	-0.6296 (-0.8984, -0.4345)	0.1169 (0.0679, 0.1743)	-22.8012 (-41.8201, -11.8159)
1m-PFOS	0.2065 (-0.0975, 0.4403)	0.1621 (-0.1353, 0.4156)	0.0444 (-0.0324, 0.1265)	21.4927 (-92.1486, 215.6442)
iso-PFOS	0.1920 (-0.2215, 0.5196)	0.1302 (-0.3441, 0.5093)	0.0618 (-0.0478, 0.1782)	32.1663 (-331.4828, 438.5192)
3 + 4 + 5 m-PFOS	-0.2879 (-0.3615, -0.2288)	-0.3062 (-0.3770, -0.2467)	0.0183 (0.0075, 0.0309)	-6.3575 (-11.7231, -2.4149)
∑m2-PFOS	0.1913 (-0.0907, 0.4332)	0.1351 (-0.1727, 0.4205)	0.0562 (-0.0386, 0.1417)	29.3775 (-206.2343, 207.0367)
PFHpS	0.1777 (-0.1039, 0.4156)	0.1153 (-0.1920, 0.3771)	0.0624 (-0.0289, 0.1542)	35.1198 (-255.2671, 247.3936)
Total PFHxS	0.1891 (-0.0074, 0.3802)	0.1341 (-0.0837, 0.3425)	0.0550 (-0.0182, 0.1359)	29.1010 (-59.6953, 199.6285)
n-PFHxS	0.1931 (0.0085, 0.3959)	0.1403 (-0.0692, 0.3477)	0.0528 (-0.0255, 0.1297)	27.3573 (-36.4323, 196.2438)
Br-PFHxS	-0.0623 (-0.1972, 0.0897)	-0.1028 (-0.2508, 0.0460)	0.0405 (0.0027, 0.0745)	-65.0889 (-710.0523, 665.8719)
PFOA	-0.0557 (-0.3349, 0.2069)	-0.1367 (-0.4411, 0.1759)	0.0810 (-0.0096, 0.1719)	-145.4800 (-727.4604, 588.7380)
PFHpA	0.0455 (-0.0793, 0.1754)	0.0155 (-0.1121, 0.1540)	0.0300 (-0.0065, 0.0643)	65.9819 (-502.0960, 642.2178)
PFHxA	0.0481 (-0.0802, 0.1901)	0.0371 (-0.0890, 0.1866)	0.0110 (-0.0013, 0.0260)	22.9008 (-337.6175, 226.3317)
PFNA	0.2064 (-0.1850, 0.5202)	0.1264 (-0.3115, 0.4842)	0.0800 (-0.0345, 0.2041)	38.7553 (-280.8691, 395.2467)
PFDA	0.1470 (-0.2647, 0.4345)	0.0799 (-0.3591, 0.3969)	0.0670 (-0.0185, 0.1564)	45.6107 (-398.6190, 333.9373)
PFUnDA	0.1243 (-0.2688, 0.4124)	0.0634 (-0.3437, 0.3793)	0.0609 (-0.0144, 0.1345)	48.9662 (-561.1369, 418.9105)
PFDoDA	0.1151 (-0.1636, 0.3526)	0.0675 (-0.2314, 0.3150)	0.0476 (-0.0084, 0.1068)	41.3735 (-480.2004, 436.2846)
PFTrDA	0.1578 (-0.1047, 0.3666)	0.1194 (-0.1452, 0.3424)	0.0384 (-0.0112, 0.0908)	24.3326 (-224.1016, 207.7785)
**PFAS[a]->GLB->GripI**				
Total PFOS	-0.0794 (-0.5679, 0.2628)	-0.1262 (-0.6792, 0.2434)	0.0468 (-0.0336, 0.1410)	-58.9183 (-403.3274, 336.2726)
n-PFOS	0.1437 (-0.2466, 0.4327)	0.1340 (-0.3105, 0.4561)	0.0097 (-0.0678, 0.0900)	6.7514 (-206.1320, 174.4737)
Br-PFOS	-0.5127 (-0.7847, -0.3433)	-0.5423 (-0.8259, -0.3700)	0.0296 (0.0055, 0.0626)	-5.7762 (-12.5580, -1.1181)

1m-PFOS	0.2065 (-0.0730, 0.4308)	0.2014 (-0.0902, 0.4365)	0.0051 (-0.0374, 0.0524)	2.4705 (-44.9559, 77.0316)
iso-PFOS	0.1920 (-0.2794, 0.5034)	0.1864 (-0.3390, 0.5339)	0.0056 (-0.0615, 0.0816)	2.9140 (-126.7901, 137.3246)
3 + 4 + 5 m-PFOS	-0.2879 (-0.3554, -0.2250)	-0.2924 (-0.3622, -0.2317)	0.0045 (-0.0008, 0.0106)	-1.5586 (-3.9431, 0.2554)
∑m2-PFOS	0.1913 (-0.1084, 0.4459)	0.1833 (-0.1236, 0.4520)	0.0079 (-0.0446, 0.0580)	4.1427 (-76.9769, 95.1472)
PFHpS	0.1777 (-0.1027, 0.4113)	0.1689 (-0.1253, 0.4237)	0.0088 (-0.0426, 0.0558)	4.9537 (-103.2524, 124.1744)
Total PFHxS	0.1891 (-0.0063, 0.3973)	0.1795 (-0.0321, 0.3866)	0.0096 (-0.0275, 0.0486)	5.0835 (-38.8723, 72.6634)
n-PFHxS	0.1931 (-0.0111, 0.3832)	0.1840 (-0.0253, 0.3735)	0.0091 (-0.0332, 0.0525)	4.7201 (-33.4353, 43.3787)
Br-PFHxS	-0.0623 (-0.2171, 0.0750)	-0.0724 (-0.2304, 0.0679)	0.0102 (-0.0078, 0.0305)	-16.3602 (-162.7271, 121.8866)
PFOA	-0.0557 (-0.3146, 0.2591)	-0.0764 (-0.3487, 0.2534)	0.0208 (-0.0211, 0.0645)	-37.2861 (-257.2687, 204.1554)
PFHpA	0.0455 (-0.0752, 0.1732)	0.0397 (-0.0818, 0.1670)	0.0058 (-0.0085, 0.0232)	12.7821 (-162.6867, 159.8104)
PFHxA	0.0481 (-0.0743, 0.1793)	0.0454 (-0.0784, 0.1767)	0.0027 (-0.0040, 0.0111)	5.5719 (-54.6807, 51.6899)
PFNA	0.2064 (-0.2023, 0.5260)	0.1957 (-0.2416, 0.5337)	0.0107 (-0.0650, 0.0827)	5.1957 (-133.1000, 153.6924)
PFDA	0.1470 (-0.2420, 0.4490)	0.1361 (-0.2755, 0.4666)	0.0109 (-0.0577, 0.0743)	7.3996 (-154.5866, 122.6826)
PFUnDA	0.1243 (-0.2441, 0.4164)	0.1121 (-0.2802, 0.4264)	0.0122 (-0.0443, 0.0668)	9.8445 (-84.0940, 153.1098)
PFDoDA	0.1151 (-0.1767, 0.3371)	0.1059 (-0.1954, 0.3401)	0.0093 (-0.0261, 0.0473)	8.0601 (-103.7944, 90.8994)
PFTrDA	0.1578 (-0.1040, 0.3932)	0.1503 (-0.1299, 0.3833)	0.0075 (-0.0250, 0.0458)	4.7361 (-72.7691, 87.8912)
**PFAS[a]->TP->GripI**				
Total PFOS	-0.0794 (-0.5181, 0.2329)	-0.0948 (-0.5573, 0.2406)	0.0154 (-0.0084, 0.0502)	-19.4557 (-94.6925, 66.0352)
n-PFOS	0.1437 (-0.2403, 0.4346)	0.1309 (-0.2648, 0.4304)	0.0128 (-0.0095, 0.0453)	8.8897 (-87.9381, 87.6569)
Br-PFOS	-0.5127 (-0.7453, -0.3372)	-0.5166 (-0.7529, -0.3368)	0.0039 (-0.0045, 0.0171)	-0.7608 (-2.8836, 0.8767)
1m-PFOS	0.2065 (-0.0593, 0.4330)	0.1975 (-0.0796, 0.4311)	0.0090 (-0.0054, 0.0353)	4.3567 (-36.2840, 58.0835)
iso-PFOS	0.1920 (-0.2599, 0.5176)	0.1791 (-0.3004, 0.5129)	0.0129 (-0.0119, 0.0466)	6.7116 (-140.8014, 91.0335)
3 + 4 + 5 m-PFOS	-0.2879 (-0.3573, -0.2284)	-0.2878 (-0.3584, -0.2283)	-0.0002 (-0.0042, 0.0039)	0.0572 (-1.3412, 1.3210)
∑m2-PFOS	0.1913 (-0.0895, 0.4400)	0.1805 (-0.1147, 0.4407)	0.0107 (-0.0093, 0.0398)	5.6093 (-77.6993, 81.0761)

PFHpS	0.1777 (-0.1052, 0.4193)	0.1645 (-0.1521, 0.4203)	0.0132 (-0.0118, 0.0445)	7.4383 (-97.0481, 92.6909)
Total PFHxS	0.1891 (-0.0099, 0.3812)	0.1804 (-0.0226, 0.3670)	0.0088 (-0.0065, 0.0343)	4.6328 (-17.5096, 57.3947)
n-PFHxS	0.1931 (-0.0045, 0.3886)	0.1841 (-0.0231, 0.3810)	0.0090 (-0.0052, 0.0358)	4.6617 (-9.2203, 41.1714)
Br-PFHxS	-0.0623 (-0.2054, 0.0882)	-0.0655 (-0.2088, 0.0865)	0.0032 (-0.0052, 0.0174)	-5.2168 (-89.5640, 67.8569)
PFOA	-0.0557 (-0.3361, 0.2203)	-0.0624 (-0.3425, 0.2070)	0.0067 (-0.0081, 0.0359)	-12.1132 (-88.7885, 106.8099)
PFHpA	0.0455 (-0.0787, 0.1663)	0.0378 (-0.0857, 0.1567)	0.0077 (-0.0043, 0.0287)	16.9873 (-148.3432, 152.2414)
PFHxA	0.0481 (-0.0676, 0.1911)	0.0449 (-0.0692, 0.1859)	0.0031 (-0.0032, 0.0164)	6.5007 (-56.3791, 62.8150)
PFNA	0.2064 (-0.2196, 0.5051)	0.1929 (-0.2560, 0.4993)	0.0135 (-0.0135, 0.0502)	6.5278 (-85.9721, 116.8074)
PFDA	0.1470 (-0.2372, 0.4408)	0.1387 (-0.2605, 0.4414)	0.0083 (-0.0073, 0.0297)	5.6259 (-54.4118, 60.5339)
PFUnDA	0.1243 (-0.3071, 0.4232)	0.1182 (-0.3149, 0.4201)	0.0061 (-0.0056, 0.0248)	4.9170 (-53.5351, 46.7833)
PFDoDA	0.1151 (-0.1882, 0.3539)	0.1121 (-0.1919, 0.3484)	0.0031 (-0.0062, 0.0164)	2.6707 (-37.2436, 29.4477)
PFTrDA	0.1578 (-0.1321, 0.3889)	0.1541 (-0.1258, 0.3901)	0.0037 (-0.0053, 0.0185)	2.3626 (-30.2679, 29.1000)
**PFAS[a]->ALT->GripI**				
Total PFOS	-0.0794 (-0.5425, 0.2386)	-0.0884 (-0.5595, 0.2333)	0.0090 (-0.0189, 0.0404)	-11.3046 (-148.8160, 81.1523)
n-PFOS	0.1437 (-0.2994, 0.4338)	0.1408 (-0.3105, 0.4298)	0.0028 (-0.0180, 0.0256)	1.9668 (-37.3041, 37.7757)
Br-PFOS	-0.5127 (-0.7798, -0.3402)	-0.5309 (-0.8015, -0.3518)	0.0183 (-0.0031, 0.0427)	-3.5614 (-9.3319, 0.5757)
1m-PFOS	0.2065 (-0.0625, 0.4195)	0.2069 (-0.0687, 0.4327)	-0.0005 (-0.0307, 0.0344)	-0.2212 (-55.7297, 47.9936)
iso-PFOS	0.1920 (-0.2434, 0.5076)	0.1903 (-0.2584, 0.5105)	0.0017 (-0.0282, 0.0329)	0.8688 (-60.0944, 47.1497)
3 + 4 + 5 m-PFOS	-0.2879 (-0.3613, -0.2222)	-0.2924 (-0.3639, -0.2256)	0.0044 (-0.0012, 0.0119)	-1.5342 (-4.4550, 0.3849)
∑m2-PFOS	0.1913 (-0.1105, 0.4496)	0.1904 (-0.1216, 0.4483)	0.0008 (-0.0356, 0.0370)	0.4435 (-74.1467, 52.2513)
PFHpS	0.1777 (-0.1008, 0.4242)	0.1765 (-0.1076, 0.4157)	0.0012 (-0.0337, 0.0359)	0.6784 (-51.1951, 60.6725)
Total PFHxS	0.1891 (-0.0240, 0.3844)	0.1872 (-0.0295, 0.3860)	0.0019 (-0.0309, 0.0347)	1.0099 (-50.5603, 55.6703)
n-PFHxS	0.1931 (-0.0002, 0.3933)	0.1915 (-0.0040, 0.3908)	0.0016 (-0.0328, 0.0369)	0.8162 (-38.9248, 35.2337)
Br-PFHxS	-0.0623 (-0.2028, 0.0899)	-0.0655 (-0.2020, 0.0811)	0.0033 (-0.0080, 0.0173)	-5.2289 (-97.7534, 82.3206)
PFOA	-0.0557 (-0.3216, 0.2097)	-0.0655 (-0.3372, 0.2066)	0.0098 (-0.0295, 0.0452)	-17.5659 (-161.9806, 145.2325)
PFHpA	0.0455 (-0.0784, 0.1713)	0.0427 (-0.0837, 0.1732)	0.0029 (-0.0149, 0.0197)	6.2682 (-134.7454, 161.0949)

PFHxA	0.0481 (-0.0696, 0.1881)	0.0471 (-0.0703, 0.1874)	0.0009 (-0.0044, 0.0063)	1.9136 (-32.7106, 35.9855)
PFNA	0.2064 (-0.1996, 0.5303)	0.2036 (-0.2115, 0.5343)	0.0028 (-0.0316, 0.0399)	1.3536 (-76.6410, 59.7116)
PFDA	0.1470 (-0.2919, 0.4369)	0.1444 (-0.2895, 0.4296)	0.0026 (-0.0127, 0.0202)	1.7357 (-30.8464, 32.7642)
PFUnDA	0.1243 (-0.2795, 0.4033)	0.1222 (-0.2843, 0.3961)	0.0021 (-0.0093, 0.0156)	1.6669 (-24.1741, 27.5181)
PFDoDA	0.1151 (-0.2063, 0.3423)	0.1131 (-0.2162, 0.3399)	0.0020 (-0.0096, 0.0156)	1.7449 (-21.6231, 37.5815)
PFTrDA	0.1578 (-0.1166, 0.4018)	0.1560 (-0.1157, 0.3966)	0.0018 (-0.0096, 0.0161)	1.1616 (-24.4575, 27.2779)
**PFAS[a]->AST->GripI**				
Total PFOS	-0.0794 (-0.5432, 0.2600)	-0.0898 (-0.5496, 0.2525)	0.0105 (-0.0154, 0.0415)	-13.1671 (-80.8226, 90.8526)
n-PFOS	0.1437 (-0.3104, 0.4556)	0.1388 (-0.3172, 0.4517)	0.0048 (-0.0187, 0.0306)	3.3613 (-53.7901, 56.1279)
Br-PFOS	-0.5127 (-0.7892, -0.3365)	-0.5234 (-0.7979, -0.3494)	0.0107 (-0.0035, 0.0314)	-2.0897 (-6.7003, 0.6886)
1m-PFOS	0.2065 (-0.0577, 0.4417)	0.2037 (-0.0658, 0.4345)	0.0028 (-0.0263, 0.0333)	1.3565 (-50.8175, 46.7107)
iso-PFOS	0.1920 (-0.2583, 0.5037)	0.1878 (-0.2675, 0.5005)	0.0042 (-0.0225, 0.0337)	2.1984 (-33.8627, 44.3115)
3 + 4 + 5 m-PFOS	-0.2879 (-0.3546, -0.2201)	-0.2896 (-0.3573, -0.2220)	0.0016 (-0.0015, 0.0073)	-0.5671 (-2.5522, 0.5135)
∑m2-PFOS	0.1913 (-0.1019, 0.4447)	0.1871 (-0.1013, 0.4391)	0.0042 (-0.0293, 0.0394)	2.1732 (-33.0002, 57.5014)
PFHpS	0.1777 (-0.0885, 0.4057)	0.1732 (-0.0940, 0.3967)	0.0045 (-0.0272, 0.0409)	2.5527 (-41.7769, 53.0074)
Total PFHxS	0.1891 (-0.0242, 0.3797)	0.1842 (-0.0277, 0.3693)	0.0049 (-0.0225, 0.0402)	2.5803 (-35.1351, 57.8424)
n-PFHxS	0.1931 (0.0005, 0.4010)	0.1885 (0.0032, 0.3941)	0.0046 (-0.0235, 0.0387)	2.3787 (-26.1414, 41.3641)
Br-PFHxS	-0.0623 (-0.2045, 0.0892)	-0.0675 (-0.2115, 0.0853)	0.0052 (-0.0095, 0.0200)	-8.4014 (-112.1954, 108.7699)
PFOA	-0.0557 (-0.3217, 0.2187)	-0.0665 (-0.3380, 0.2201)	0.0108 (-0.0193, 0.0455)	-19.3822 (-168.2002, 111.2993)
PFHpA	0.0455 (-0.0720, 0.1688)	0.0413 (-0.0787, 0.1675)	0.0042 (-0.0106, 0.0199)	9.1811 (-124.5277, 117.7908)
PFHxA	0.0481 (-0.0697, 0.1765)	0.0458 (-0.0750, 0.1756)	0.0023 (-0.0066, 0.0121)	4.7568 (-48.1863, 99.7127)
PFNA	0.2064 (-0.2313, 0.5279)	0.2007 (-0.2438, 0.5167)	0.0057 (-0.0260, 0.0407)	2.7827 (-33.2970, 69.1754)
PFDA	0.1470 (-0.2488, 0.4283)	0.1428 (-0.2486, 0.4269)	0.0042 (-0.0137, 0.0257)	2.8439 (-34.8945, 56.3042)
PFUnDA	0.1243 (-0.2714, 0.4168)	0.1208 (-0.2715, 0.4151)	0.0036 (-0.0094, 0.0200)	2.8572 (-36.5672, 35.2101)
PFDoDA	0.1151 (-0.2092, 0.3359)	0.1120 (-0.2104, 0.3309)	0.0032 (-0.0097, 0.0172)	2.7506 (-35.1889, 44.4593)
PFTrDA	0.1578 (-0.1295, 0.3754)	0.1555 (-0.1278, 0.3726)	0.0023 (-0.0070, 0.0159)	1.4568 (-20.5606, 29.9891)

**PFAS[a]->GGT->GripI**				
Total PFOS	-0.0794 (-0.5396, 0.2428)	-0.0776 (-0.5368, 0.2450)	-0.0018 (-0.0174, 0.0184)	2.2184 (-50.6019, 53.1573)
n-PFOS	0.1437 (-0.2902, 0.4186)	0.1470 (-0.2857, 0.4250)	-0.0034 (-0.0164, 0.0114)	-2.3343 (-25.9492, 22.6789)
Br-PFOS	-0.5127 (-0.7754, -0.3298)	-0.5158 (-0.7762, -0.3324)	0.0031 (-0.0064, 0.0216)	-0.6009 (-4.4420, 1.3770)
1m-PFOS	0.2065 (-0.0689, 0.4310)	0.2100 (-0.0631, 0.4317)	-0.0035 (-0.0115, 0.0114)	-1.6990 (-19.9693, 13.7958)
iso-PFOS	0.1920 (-0.2467, 0.5107)	0.1971 (-0.2577, 0.5151)	-0.0051 (-0.0205, 0.0149)	-2.6360 (-30.4832, 27.8199)
3 + 4 + 5 m-PFOS	-0.2879 (-0.3627, -0.2242)	-0.2883 (-0.3641, -0.2255)	0.0003 (-0.0019, 0.0045)	-0.1184 (-1.5566, 0.6970)
∑m2-PFOS	0.1913 (-0.1061, 0.4442)	0.1981 (-0.1088, 0.4513)	-0.0069 (-0.0259, 0.0172)	-3.5829 (-37.3644, 42.8674)
PFHpS	0.1777 (-0.0869, 0.4128)	0.1813 (-0.0890, 0.4166)	-0.0036 (-0.0136, 0.0124)	-2.0202 (-26.3406, 27.2790)
Total PFHxS	0.1891 (-0.0102, 0.4040)	0.1915 (-0.0101, 0.4038)	-0.0024 (-0.0106, 0.0120)	-1.2623 (-20.4577, 14.2966)
n-PFHxS	0.1931 (-0.0013, 0.3903)	0.1955 (0.0012, 0.3913)	-0.0024 (-0.0097, 0.0103)	-1.2556 (-12.9923, 15.0872)
Br-PFHxS	-0.0623 (-0.2100, 0.0755)	-0.0618 (-0.2115, 0.0751)	-0.0004 (-0.0031, 0.0079)	0.6856 (-25.6642, 16.3557)
PFOA	-0.0557 (-0.3286, 0.2228)	-0.0534 (-0.3334, 0.2303)	-0.0023 (-0.0273, 0.0154)	4.0635 (-73.4019, 91.6091)
PFHpA	0.0455 (-0.0775, 0.1770)	0.0489 (-0.0755, 0.1807)	-0.0034 (-0.0231, 0.0114)	-7.4025 (-132.7688, 113.5140)
PFHxA	0.0481 (-0.0756, 0.1826)	0.0488 (-0.0754, 0.1851)	-0.0008 (-0.0052, 0.0040)	-1.5842 (-26.7658, 34.4961)
PFNA	0.2064 (-0.2336, 0.5314)	0.2114 (-0.2192, 0.5342)	-0.0050 (-0.0250, 0.0182)	-2.4364 (-37.0170, 37.2004)
PFDA	0.1470 (-0.2259, 0.4438)	0.1492 (-0.2316, 0.4451)	-0.0023 (-0.0132, 0.0093)	-1.5499 (-25.6808, 24.0078)
PFUnDA	0.1243 (-0.2988, 0.4053)	0.1258 (-0.2995, 0.4059)	-0.0015 (-0.0081, 0.0072)	-1.1738 (-18.4552, 13.9611)
PFDoDA	0.1151 (-0.1722, 0.3312)	0.1169 (-0.1711, 0.3331)	-0.0017 (-0.0104, 0.0071)	-1.5069 (-21.7605, 27.6936)
PFTrDA	0.1578 (-0.1229, 0.3806)	0.1598 (-0.1241, 0.3820)	-0.0020 (-0.0126, 0.0065)	-1.2718 (-25.1519, 30.6971)

Abbreviations: ALB, albumin; GLB, globin; TP, total protein; ALT, Alanine aminotransferase; AST, Aspartate Aminotransferase; GGT, Gamma-Glutamyl Transferase.

Model was adjusted for age, sex, education, alcohol drinking, smoking, family income, exercise, BMI, district.

Bolding indicates that associations were statistically significant ($P < 0{\cdot}05$).

[a] The variables were natural-log transformed.

Table S11. The mediation of markers of liver function in the association between sarcopenia and PFAS in serum excluding CKD, malnutrition, and inflammation (N=989).

Pathways	Total effect	Direct effect	Indirect effect	Prop mediated
PFASa->ALB->Sarcopenia				
Total PFOS	**0.0094 (0.0049, 0.0137)**	**0.0081 (0.0038, 0.0134)**	0.0012 (-0.0018, 0.0054)	13.1646 (-19.5616, 50.0064)
n-PFOS	**0.0130 (0.0077, 0.0177)**	**0.0113 (0.0057, 0.0163)**	0.0017 (-0.0020, 0.0083)	13.2468 (-16.8731, 51.7665)
Br-PFOS	**0.0324 (0.0248, 0.0402)**	**0.0285 (0.0188, 0.0374)**	0.0040 (-0.0008, 0.0098)	12.2204 (-2.5287, 31.8847)
1m-PFOS	**0.0450 (0.0090, 0.1084)**	0.0175 (-0.0174, 0.0786)	**0.0274 (0.0073, 0.0492)**	**61.0343 (10.5111, 236.3141)**
iso-PFOS	**0.0571 (0.0216, 0.0924)**	0.0361 (-0.0056, 0.0758)	**0.0209 (0.0003, 0.0449)**	**36.6897 (0.4513, 118.1986)**
3 + 4 + 5m-PFOS	**0.0411 (0.0268, 0.0574)**	**0.0394 (0.0245, 0.0562)**	**0.0017 (0.0002, 0.0036)**	**4.1853 (0.4153, 10.1287)**
∑m2-PFOS	**0.0758 (0.0137, 0.1573)**	0.0280 (-0.0224, 0.1067)	**0.0478 (0.0146, 0.0858)**	**63.0389 (21.3874, 235.7358)**
PFHpS	**0.0764 (0.0163, 0.1644)**	0.0414 (-0.0227, 0.1216)	**0.0350 (0.0063, 0.0706)**	**45.8730 (7.8501, 211.2108)**
Total PFHxS	**0.0336 (0.0031, 0.0775)**	0.0071 (-0.0246, 0.0507)	**0.0265 (0.0092, 0.0443)**	**78.9801 (18.0137, 494.4841)**
n-PFHxS	**0.0306 (0.0026, 0.0732)**	0.0042 (-0.0254, 0.0466)	**0.0264 (0.0100, 0.0460)**	**86.1740 (11.7523, 446.2630)**
Br-PFHxS	**0.0395 (0.0007, 0.1105)**	0.0170 (-0.0147, 0.0838)	**0.0224 (0.0078, 0.0397)**	**56.8709 (7.9364, 318.0616)**
PFOA	**0.0147 (0.0062, 0.0216)**	**0.0121 (0.0050, 0.0175)**	**0.0026 (0.0002, 0.0089)**	**17.7283 (1.6326, 47.4779)**
PFHpA	**0.0407 (0.0044, 0.1037)**	0.0218 (-0.0092, 0.0840)	**0.0188 (0.0061, 0.0328)**	**46.2870 (11.0658, 232.2786)**
PFHxA	0.0009 (-0.0075, 0.0230)	-0.0021 (-0.0095, 0.0182)	**0.0030 (0.0008, 0.0070)**	343.6940 (-759.5437, 646.7167)
PFNA	**0.0786 (0.0427, 0.1230)**	**0.0586 (0.0170, 0.1083)**	0.0200 (-0.0034, 0.0452)	25.4389 (-4.6761, 67.9425)
PFDA	**0.0783 (0.0451, 0.1219)**	**0.0630 (0.0272, 0.1108)**	0.0153 (-0.0050, 0.0373)	19.5744 (-6.3852, 50.9657)
PFUnDA	**0.0733 (0.0331, 0.1233)**	**0.0554 (0.0122, 0.1093)**	0.0180 (-0.0005, 0.0343)	24.4946 (-0.7064, 63.5027)
PFDoDA	**0.1122 (0.0403, 0.1935)**	**0.0840 (0.0159, 0.1787)**	**0.0282 (0.0008, 0.0575)**	**25.1656 (0.7984, 67.7488)**
PFTrDA	**0.0785 (0.0280, 0.1654)**	**0.0589 (0.0095, 0.1450)**	**0.0197 (0.0032, 0.0366)**	**25.0311 (3.6629, 68.7617)**
PFASa->GLB->Sarcopenia				
Total PFOS	**0.0094 (0.0048, 0.0143)**	**0.0075 (0.0039, 0.0110)**	**0.0019 (0.0006, 0.0050)**	**20.2927 (8.5484, 41.2750)**
n-PFOS	**0.0127 (0.0079, 0.0175)**	**0.0101 (0.0062, 0.0136)**	**0.0026 (0.0009, 0.0069)**	**20.2722 (8.8539, 44.5300)**
Br-PFOS	**0.0329 (0.0237, 0.0400)**	**0.0295 (0.0202, 0.0367)**	**0.0034 (0.0015, 0.0061)**	**10.2762 (4.6446, 19.8334)**
1m-PFOS	**0.0459 (0.0079, 0.1125)**	0.0328 (-0.0023, 0.0918)	**0.0131 (0.0067, 0.0221)**	**28.5299 (11.8036, 112.2955)**

iso-PFOS	**0.0544 (0.0192, 0.0897)**	0.0401 (0.0069, 0.0753)	**0.0143 (0.0068, 0.0232)**	**26.2861 (12.2140, 67.8527)**
3 + 4 + 5m-PFOS	**0.0413 (0.0271, 0.0580)**	0.0400 (0.0257, 0.0571)	**0.0012 (0.0004, 0.0025)**	**3.0037 (1.0526, 6.5768)**
∑m2-PFOS	**0.0713 (0.0111, 0.1484)**	0.0456 (-0.0035, 0.1151)	**0.0257 (0.0109, 0.0424)**	**36.0235 (15.5774, 122.4096)**
PFHpS	**0.0817 (0.0235, 0.1648)**	**0.0641 (0.0077, 0.1400)**	**0.0176 (0.0087, 0.0288)**	**21.5559 (9.7517, 66.6190)**
Total PFHxS	**0.0392 (0.0077, 0.0841)**	0.0290 (-0.0015, 0.0733)	**0.0102 (0.0051, 0.0168)**	**25.9363 (11.2540, 111.7224)**
n-PFHxS	**0.0351 (0.0032, 0.0812)**	0.0253 (-0.0052, 0.0687)	**0.0098 (0.0052, 0.0160)**	**27.9458 (11.1304, 170.1262)**
Br-PFHxS	**0.0481 (0.0054, 0.1128)**	**0.0393 (0.0007, 0.1029)**	**0.0088 (0.0029, 0.0163)**	**18.3139 (5.0881, 78.8659)**
PFOA	**0.0141 (0.0061, 0.0216)**	**0.0124 (0.0056, 0.0186)**	**0.0017 (0.0005, 0.0049)**	**12.0958 (5.0990, 24.8498)**
PFHpA	**0.0321 (0.0043, 0.0860)**	0.0252 (-0.0002, 0.0775)	**0.0069 (0.0020, 0.0142)**	**21.5959 (5.2499, 90.8303)**
PFHxA	0.0010 (-0.0074, 0.0247)	-0.0009 (-0.0086, 0.0219)	**0.0019 (0.0001, 0.0054)**	195.3509 (-318.2897, 467.9093)
PFNA	**0.0757 (0.0407, 0.1211)**	**0.0601 (0.0254, 0.1032)**	**0.0156 (0.0075, 0.0248)**	**20.6276 (9.4099, 39.9402)**
PFDA	**0.0770 (0.0420, 0.1202)**	**0.0622 (0.0265, 0.1047)**	**0.0149 (0.0069, 0.0243)**	**19.3333 (8.2654, 39.3657)**
PFUnDA	**0.0725 (0.0348, 0.1223)**	**0.0575 (0.0210, 0.1077)**	**0.0150 (0.0073, 0.0243)**	**20.6573 (8.5515, 41.9894)**
PFDoDA	**0.1065 (0.0354, 0.1898)**	**0.0862 (0.0222, 0.1664)**	**0.0202 (0.0092, 0.0316)**	**19.0180 (8.0117, 42.2867)**
PFTrDA	**0.0719 (0.0241, 0.1519)**	**0.0575 (0.0140, 0.1322)**	**0.0144 (0.0065, 0.0250)**	**20.0386 (8.3214, 46.6436)**
PFASa->TP->Sarcopenia				
Total PFOS	**0.0094 (0.0049, 0.0144)**	**0.0094 (0.0049, 0.0143)**	0.0001 (-0.0002, 0.0004)	0.5695 (-1.9227, 3.7307)
n-PFOS	**0.0129 (0.0079, 0.0173)**	**0.0129 (0.0079, 0.0173)**	0.0001 (-0.0003, 0.0004)	0.4786 (-2.1586, 3.2954)
Br-PFOS	**0.0333 (0.0257, 0.0408)**	**0.0333 (0.0256, 0.0408)**	0.0001 (-0.0003, 0.0005)	0.1618 (-0.8907, 1.4336)
1m-PFOS	**0.0482 (0.0091, 0.1079)**	**0.0477 (0.0088, 0.1062)**	0.0005 (-0.0010, 0.0025)	0.9732 (-3.4823, 6.3215)
iso-PFOS	**0.0557 (0.0235, 0.0925)**	**0.0553 (0.0233, 0.0916)**	0.0004 (-0.0011, 0.0025)	0.7446 (-2.5228, 5.0114)
3 + 4 + 5m-PFOS	**0.0455 (0.0305, 0.0613)**	**0.0455 (0.0305, 0.0614)**	-0.0000 (-0.0003, 0.0002)	-0.0265 (-0.6184, 0.5132)
∑m2-PFOS	**0.0670 (0.0124, 0.1501)**	**0.0663 (0.0125, 0.1497)**	0.0007 (-0.0014, 0.0044)	1.0840 (-2.6318, 7.4443)
PFHpS	**0.0827 (0.0231, 0.1618)**	**0.0820 (0.0229, 0.1611)**	0.0007 (-0.0012, 0.0032)	0.8644 (-1.8915, 5.1007)
Total PFHxS	**0.0371 (0.0041, 0.0793)**	**0.0369 (0.0040, 0.0793)**	0.0002 (-0.0010, 0.0014)	0.5407 (-3.9812, 7.6114)
n-PFHxS	**0.0341 (0.0043, 0.0782)**	**0.0339 (0.0036, 0.0784)**	0.0002 (-0.0009, 0.0017)	0.6917 (-3.9312, 9.7197)
Br-PFHxS	**0.0402 (0.0028, 0.1076)**	**0.0401 (0.0024, 0.1070)**	0.0001 (-0.0014, 0.0020)	0.3150 (-6.1688, 10.4585)

PFOA	**0.0144 (0.0062, 0.0215)**	**0.0144 (0.0063, 0.0214)**	0.0000 (-0.0001, 0.0003)	0.3007 (-0.7660, 1.9835)
PFHpA	**0.0399 (0.0050, 0.1078)**	**0.0393 (0.0047, 0.1075)**	0.0006 (-0.0017, 0.0031)	1.3995 (-4.5184, 14.7488)
PFHxA	-0.0003(-0.0076,0.0218)	-0.0003 (-0.0077, 0.0214)	-0.0000 (-0.0005, 0.0006)	3.6519 (-53.2559, 35.4885)
PFNA	**0.0800 (0.0434, 0.1267)**	**0.0796 (0.0425, 0.1269)**	0.0004 (-0.0013, 0.0023)	0.4973 (-1.7415, 3.1490)
PFDA	**0.0800 (0.0444, 0.1279)**	**0.0797 (0.0448, 0.1274)**	0.0003 (-0.0012, 0.0021)	0.3508 (-1.4919, 2.6696)
PFUnDA	**0.0754 (0.0356, 0.1280)**	**0.0752 (0.0358, 0.1279)**	0.0002 (-0.0012, 0.0020)	0.2629 (-1.7626, 2.6464)
PFDoDA	**0.1193 (0.0456, 0.1925)**	**0.1192 (0.0453, 0.1919)**	0.0001 (-0.0021, 0.0020)	0.0897 (-1.7821, 2.1532)
PFTrDA	**0.0861 (0.0272, 0.1623)**	**0.0860 (0.0272, 0.1624)**	0.0001 (-0.0012, 0.0016)	0.1452 (-1.6732, 2.0582)
PFASa->ALT->Sarcopenia				
Total PFOS	**0.0095 (0.0047, 0.0141)**	**0.0094 (0.0047, 0.0142)**	0.0001 (-0.0005, 0.0004)	0.5358 (-5.1189, 3.4033)
n-PFOS	**0.0130 (0.0075, 0.0176)**	**0.0129 (0.0076, 0.0175)**	0.0001 (-0.0005, 0.0004)	0.4665 (-3.6368, 2.7526)
Br-PFOS	**0.0332 (0.0248, 0.0416)**	**0.0330 (0.0245, 0.0415)**	0.0001 (-0.0014, 0.0010)	0.4510 (-4.5424, 3.5226)
1m-PFOS	**0.0482 (0.0101, 0.1153)**	**0.0471 (0.0093, 0.1154)**	0.0010 (-0.0047, 0.0043)	2.1709 (-13.0101, 13.8881)
iso-PFOS	**0.0556 (0.0233, 0.0921)**	**0.0550 (0.0236, 0.0926)**	0.0006 (-0.0030, 0.0026)	1.0769 (-5.8088, 5.2277)
3 + 4 + 5m-PFOS	**0.0457 (0.0290, 0.0621)**	**0.0457 (0.0290, 0.0622)**	0.0000 (-0.0006, 0.0005)	0.0982 (-1.3873, 1.1772)
∑m2-PFOS	**0.0657 (0.0122, 0.1472)**	**0.0644 (0.0117, 0.1463)**	0.0013 (-0.0074, 0.0053)	1.9975 (-12.6263, 13.2054)
PFHpS	**0.0829 (0.0228, 0.1649)**	**0.0815 (0.0228, 0.1648)**	0.0014 (-0.0056, 0.0057)	1.6491 (-9.2592, 8.9519)
Total PFHxS	**0.0371 (0.0058, 0.0789)**	**0.0360 (0.0056, 0.0785)**	0.0011 (-0.0034, 0.0044)	2.9690 (-12.7686, 18.9698)
n-PFHxS	**0.0343 (0.0054, 0.0800)**	**0.0332 (0.0034, 0.0785)**	0.0011 (-0.0032, 0.0041)	3.2118 (-14.6636, 20.2541)
Br-PFHxS	**0.0407 (0.0017, 0.1061)**	**0.0401 (0.0017, 0.1059)**	0.0006 (-0.0015, 0.0028)	1.5070 (-5.1946, 11.6017)
PFOA	**0.0144 (0.0062, 0.0214)**	**0.0142 (0.0063, 0.0212)**	0.0002 (-0.0008, 0.0007)	1.0696 (-5.2963, 4.3305)
PFHpA	**0.0408 (0.0043, 0.1075)**	**0.0400 (0.0048, 0.1076)**	0.0007 (-0.0030, 0.0033)	1.8311 (-11.8222, 16.5627)
PFHxA	-0.0004(-0.0077,0.0195)	-0.0004 (-0.0078, 0.0194)	0.0001 (-0.0006, 0.0006)	-14.5489 (-31.7746, 47.8286)
PFNA	**0.0806 (0.0436, 0.1240)**	**0.0798 (0.0433, 0.1242)**	0.0008 (-0.0043, 0.0033)	1.0089 (-5.0818, 4.5569)
PFDA	**0.0798 (0.0447, 0.1273)**	**0.0796 (0.0449, 0.1275)**	0.0003 (-0.0019, 0.0013)	0.3480 (-2.3116, 1.8662)
PFUnDA	**0.0753 (0.0386, 0.1278)**	**0.0752 (0.0384, 0.1280)**	0.0001 (-0.0018, 0.0011)	0.1665 (-2.5448, 1.5212)
PFDoDA	**0.1193 (0.0449, 0.1965)**	**0.1191 (0.0443, 0.1975)**	0.0002 (-0.0029, 0.0020)	0.1797 (-2.5459, 1.7606)
PFTrDA	**0.0884 (0.0305, 0.1780)**	**0.0880 (0.0303, 0.1776)**	0.0004 (-0.0022, 0.0019)	0.4455 (-2.5577, 2.0617)

PFASa->AST->Sarcopenia				
Total PFOS	**0.0096 (0.0049, 0.0142)**	**0.0090 (0.0046, 0.0133)**	0.0006 (-0.0002, 0.0020)	6.0929 (-1.8089, 16.4615)
n-PFOS	**0.0130 (0.0078, 0.0176)**	**0.0122 (0.0075, 0.0165)**	0.0007 (-0.0001, 0.0024)	5.7763 (-1.0664, 15.7981)
Br-PFOS	**0.0330 (0.0247, 0.0409)**	**0.0316 (0.0231, 0.0394)**	0.0013 (-0.0000, 0.0035)	4.0271 (-0.0741, 10.9745)
1m-PFOS	**0.0465 (0.0095, 0.1081)**	**0.0401 (0.0030, 0.0985)**	**0.0064 (0.0003, 0.0137)**	**13.7526 (0.4600, 51.0155)**
iso-PFOS	**0.0549 (0.0228, 0.0903)**	**0.0498 (0.0180, 0.0840)**	0.0051 (-0.0002, 0.0110)	9.2931 (-0.5277, 27.0904)
3 + 4 + 5m-PFOS	**0.0443 (0.0284, 0.0614)**	**0.0437 (0.0277, 0.0607)**	0.0006 (-0.0000, 0.0016)	1.3993 (-0.0140, 4.0237)
∑m2-PFOS	**0.0638 (0.0100, 0.1475)**	**0.0519 (0.0022, 0.1344)**	0.0119 (0.0006, 0.0260)	18.6385 (0.3800, 75.9311)
PFHpS	**0.0825 (0.0204, 0.1632)**	**0.0738 (0.0140, 0.1543)**	**0.0087 (0.0004, 0.0185)**	**10.5064 (0.3146, 37.4283)**
Total PFHxS	**0.0368 (0.0053, 0.0838)**	**0.0307 (0.0000, 0.0764)**	**0.0060 (0.0006, 0.0123)**	**16.4326 (0.2642, 76.5531)**
n-PFHxS	**0.0342 (0.0028, 0.0799)**	0.0282 (-0.0020, 0.0730)	**0.0060 (0.0008, 0.0126)**	**17.5596 (0.9209, 106.7948)**
Br-PFHxS	**0.0420 (0.0021, 0.1088)**	0.0352 (-0.0010, 0.0997)	**0.0068 (0.0009, 0.0160)**	**16.1524 (0.6289, 79.1564)**
PFOA	**0.0148 (0.0069, 0.0217)**	**0.0139 (0.0066, 0.0203)**	0.0008 (-0.0000, 0.0028)	5.5835 (-0.1253, 14.5529)
PFHpA	**0.0391 (0.0049, 0.1020)**	**0.0341 (0.0016, 0.0976)**	**0.0050 (0.0006, 0.0113)**	**12.8390 (0.5068, 59.2021)**
PFHxA	-0.0003(-0.0078,0.0258)	-0.0017 (-0.0087, 0.0235)	0.0014 (0.0000, 0.0039)	-405.4031 (-260.4901, 315.8284)
PFNA	**0.0773 (0.0403, 0.1211)**	**0.0720 (0.0351, 0.1157)**	0.0053 (-0.0008, 0.0115)	6.7992 (-1.1234, 18.1867)
PFDA	**0.0763 (0.0422, 0.1259)**	**0.0723 (0.0385, 0.1222)**	0.0039 (-0.0004, 0.0093)	5.1711 (-0.4646, 13.6285)
PFUnDA	**0.0705 (0.0326, 0.1216)**	**0.0669 (0.0293, 0.1188)**	0.0036 (-0.0002, 0.0085)	5.1123 (-0.2586, 14.8508)
PFDoDA	**0.1101 (0.0380, 0.1902)**	**0.1044 (0.0319, 0.1861)**	0.0057 (-0.0005, 0.0132)	5.2050 (-0.6230, 16.7726)
PFTrDA	**0.0799 (0.0270, 0.1647)**	**0.0759 (0.0236, 0.1619)**	0.0040 (-0.0001, 0.0095)	5.0041 (-0.1734, 16.7606)
PFASa->GGT->Sarcopenia				
Total PFOS	**0.0094 (0.0047, 0.0140)**	**0.0089 (0.0045, 0.0132)**	**0.0005 (0.0001, 0.0012)**	**5.0953 (0.8672, 10.6988)**
n-PFOS	**0.0126 (0.0080, 0.0175)**	**0.0121 (0.0075, 0.0166)**	**0.0005 (0.0001, 0.0015)**	**4.1924 (0.5583, 9.4508)**
Br-PFOS	**0.0323 (0.0241, 0.0405)**	**0.0308 (0.0230, 0.0389)**	**0.0015 (0.0003, 0.0030)**	**4.5713 (1.0628, 9.4475)**
1m-PFOS	**0.0485 (0.0090, 0.1130)**	**0.0435 (0.0053, 0.1069)**	**0.0050 (0.0010, 0.0109)**	**10.3718 (2.1783, 38.9089)**
iso-PFOS	**0.0575 (0.0243, 0.0928)**	**0.0536 (0.0217, 0.0888)**	**0.0039 (0.0007, 0.0078)**	**6.7626 (1.5973, 14.8594)**
3 + 4 + 5m-PFOS	**0.0441 (0.0286, 0.0615)**	**0.0432 (0.0278, 0.0603)**	**0.0009 (0.0002, 0.0020)**	**2.0235 (0.3889, 4.8763)**
∑m2-PFOS	**0.0706 (0.0114, 0.1537)**	**0.0626 (0.0079, 0.1444)**	**0.0080 (0.0015, 0.0162)**	**11.3968 (2.6771, 36.5881)**

PFHpS	**0.0828 (0.0237, 0.1657)**	**0.0752 (0.0185, 0.1572)**	**0.0076 (0.0020, 0.0153)**	**9.2056 (2.0483, 23.4612)**
Total PFHxS	**0.0378 (0.0059, 0.0839)**	**0.0331 (0.0026, 0.0764)**	**0.0047 (0.0012, 0.0093)**	**12.3806 (2.6567, 55.4311)**
n-PFHxS	**0.0343 (0.0037, 0.0789)**	**0.0297 (-0.0001, 0.0734)**	**0.0046 (0.0011, 0.0096)**	**13.4552 (2.6626, 62.5853)**
Br-PFHxS	**0.0443 (0.0027, 0.1116)**	**0.0418 (0.0017, 0.1078)**	0.0025 (-0.0007, 0.0074)	5.6495 (-2.8124, 26.9356)
PFOA	**0.0140 (0.0060, 0.0207)**	**0.0134 (0.0057, 0.0195)**	**0.0006 (0.0001, 0.0016)**	**4.5289 (1.1379, 8.7644)**
PFHpA	**0.0437 (0.0055, 0.1107)**	**0.0393 (0.0039, 0.1052)**	**0.0044 (0.0011, 0.0092)**	**10.0503 (2.1479, 38.8973)**
PFHxA	0.0007 (-0.0075, 0.0225)	0.0005 (-0.0077, 0.0221)	0.0003 (-0.0011, 0.0019)	36.0762 (-108.6505, 119.6740)
PFNA	**0.0823 (0.0468, 0.1248)**	**0.0768 (0.0425, 0.1191)**	**0.0055 (0.0017, 0.0100)**	**6.7246 (2.0100, 13.4495)**
PFDA	**0.0823 (0.0461, 0.1254)**	**0.0794 (0.0437, 0.1219)**	**0.0029 (0.0002, 0.0067)**	**3.5634 (0.2623, 8.1975)**
PFUnDA	**0.0765 (0.0345, 0.1260)**	**0.0744 (0.0331, 0.1234)**	0.0022 (-0.0008, 0.0057)	2.8215 (-1.2043, 7.9719)
PFDoDA	**0.1149 (0.0421, 0.1908)**	**0.1124 (0.0412, 0.1890)**	0.0025 (-0.0029, 0.0079)	2.1439 (-2.4592, 8.2114)
PFTrDA	**0.0844 (0.0324, 0.1731)**	**0.0823 (0.0311, 0.1702)**	0.0021 (-0.0013, 0.0066)	2.5002 (-2.0464, 8.9268)

Abbreviations: ALB, albumin; GLB, globin; TP, total protein; ALT, Alanine aminotransferase; AST, Aspartate Aminotransferase; GGT, Gamma-Glutamyl Transferase.

Model was adjusted for age, sex, education, alcohol drinking, smoking, family income, exercise, BMI, district.

Bolding indicates that associations were statistically significant ($P < 0{\cdot}05$).

[a] The variables were natural-log transformed.

[b] The complete mediation.

Table S12. The mediation of markers of liver function in the association between sarcopenia and PFAS in serum excluding CHD, stroke, and dyslipidemia(N=1155).

Pathways	Total effect	Direct effect	Indirect effect	Prop mediated
PFASa->ALB->Sarcopenia				
Total PFOS	**0.0137 (0.0076, 0.0187)**	**0.0121 (0.0066, 0.0181)**	0.0017 (-0.0020, 0.0065)	12.0823 (-15.1874, 41.4144)
n-PFOS	**0.0187 (0.0129, 0.0231)**	**0.0165 (0.0104, 0.0221)**	0.0022 (-0.0025, 0.0079)	11.9776 (-14.3270, 41.2230)
Br-PFOS	**0.0419 (0.0318, 0.0500)**	**0.0355 (0.0221, 0.0459)**	**0.0064 (0.0008, 0.0128)**	**15.2350 (1.9075, 34.3659)**
1m-PFOS	**0.0520 (0.0200, 0.1005)**	0.0216 (-0.0109, 0.0741)	**0.0304 (0.0131, 0.0495)**	**58.5004 (20.4204, 140.7309)**
iso-PFOS	**0.0667 (0.0348, 0.0987)**	**0.0418 (0.0023, 0.0789)**	**0.0249 (0.0051, 0.0475)**	**37.3482 (7.1861, 94.8326)**
3 + 4 + 5m-PFOS	**0.0358 (0.0193, 0.0604)**	**0.0330 (0.0160, 0.0588)**	**0.0028 (0.0009, 0.0048)**	**7.8275 (1.9152, 19.7421)**
∑m2-PFOS	**0.0810 (0.0224, 0.1440)**	0.0274 (-0.0238, 0.0980)	**0.0536 (0.0205, 0.0852)**	**66.1521 (23.6644, 183.6969)**
PFHpS	**0.0694 (0.0253, 0.1299)**	0.0296 (-0.0196, 0.0899)	**0.0398 (0.0126, 0.0684)**	**57.3316 (16.1939, 165.2700)**
Total PFHxS	**0.0306 (0.0078, 0.0674)**	-0.0012 (-0.0273, 0.0356)	**0.0318 (0.0159, 0.0494)**	**104.0587 (37.6439, 434.8688)**
n-PFHxS	**0.0287 (0.0043, 0.0662)**	-0.0038 (-0.0299, 0.0336)	**0.0325 (0.0163, 0.0491)**	**113.1175 (34.9545, 528.2268)**
Br-PFHxS	**0.0308 (0.0020, 0.0749)**	0.0079 (-0.0148, 0.0488)	**0.0229 (0.0107, 0.0359)**	**74.4042 (24.3963, 331.4903)**
PFOA	**0.0239 (0.0143, 0.0296)**	**0.0192 (0.0118, 0.0242)**	**0.0047 (0.0010, 0.0113)**	**19.5818 (5.1600, 41.9270)**
PFHpA	**0.0292 (0.0025, 0.0779)**	0.0105 (-0.0108, 0.0544)	**0.0186 (0.0088, 0.0289)**	**63.8446 (22.7071, 327.3829)**
PFHxA	0.0052 (-0.0064, 0.0301)	0.0017 (-0.0086, 0.0252)	**0.0035 (0.0010, 0.0073)**	67.5248 (-457.4920, 420.9510)
PFNA	**0.0871 (0.0496, 0.1286)**	**0.0618 (0.0201, 0.1074)**	**0.0253 (0.0024, 0.0479)**	**29.0138 (2.8240, 67.3480)**
PFDA	**0.0823 (0.0493, 0.1273)**	**0.0626 (0.0261, 0.1087)**	0.0197 (-0.0002, 0.0395)	23.9489 (-0.1564, 52.7999)
PFUnDA	**0.0622 (0.0330, 0.1038)**	**0.0391 (0.0076, 0.0843)**	**0.0231 (0.0067, 0.0388)**	**37.1879 (10.9667, 78.9305)**
PFDoDA	**0.0575 (0.0210, 0.1131)**	0.0244 (-0.0107, 0.0798)	**0.0331 (0.0149, 0.0549)**	**57.5545 (20.6659, 142.7481)**
PFTrDA	**0.0416 (0.0125, 0.0884)**	0.0186 (-0.0089, 0.0615)	**0.0230 (0.0110, 0.0375)**	**55.2634 (21.5151, 161.6328)**
PFASa->GLB->Sarcopenia				
Total PFOS	**0.0135 (0.0081, 0.0190)**	**0.0112 (0.0067, 0.0154)**	**0.0024 (0.0007, 0.0053)**	**17.6142 (6.7331, 31.8632)**
n-PFOS	**0.0187 (0.0133, 0.0233)**	**0.0155 (0.0110, 0.0192)**	**0.0032 (0.0011, 0.0067)**	**17.1579 (6.9952, 31.9332)**
Br-PFOS	**0.0425 (0.0303, 0.0508)**	**0.0381 (0.0257, 0.0467)**	**0.0044 (0.0022, 0.0077)**	**10.3947 (5.1333, 21.4462)**
1m-PFOS	**0.0548 (0.0179, 0.1082)**	**0.0408 (0.0057, 0.0913)**	**0.0140 (0.0074, 0.0220)**	**25.5940 (11.4037, 72.2578)**

iso-PFOS	**0.0651 (0.0306, 0.0988)**	**0.0497 (0.0141, 0.0841)**	**0.0154 (0.0079, 0.0249)**	**23.6799 (11.7179, 56.4467)**
3 + 4 + 5m-PFOS	**0.0385 (0.0227, 0.0620)**	**0.0371 (0.0207, 0.0607)**	**0.0015 (0.0005, 0.0027)**	**3.8028 (1.0518, 8.9206)**
∑m2-PFOS	**0.0757 (0.0165, 0.1390)**	0.0502 (-0.0002, 0.1111)	**0.0255 (0.0129, 0.0395)**	**33.6474 (14.5557, 97.8814)**
PFHpS	**0.0753 (0.0257, 0.1431)**	**0.0582 (0.0107, 0.1220)**	**0.0171 (0.0086, 0.0277)**	**22.7201 (10.3860, 60.1594)**
Total PFHxS	**0.0342 (0.0034, 0.0722)**	0.0229 (-0.0056, 0.0587)	**0.0113 (0.0060, 0.0180)**	**33.1473 (12.7809, 145.3797)**
n-PFHxS	**0.0316 (0.0042, 0.0725)**	0.0203 (-0.0059, 0.0594)	**0.0114 (0.0062, 0.0176)**	**35.9374 (14.0863, 153.2855)**
Br-PFHxS	**0.0326 (0.0032, 0.0824)**	0.0245 (-0.0028, 0.0704)	**0.0081 (0.0037, 0.0147)**	**24.9593 (8.7741, 131.9110)**
PFOA	**0.0246 (0.0151, 0.0295)**	**0.0217 (0.0137, 0.0255)**	**0.0029 (0.0010, 0.0065)**	**11.8436 (5.6461, 23.9231)**
PFHpA	**0.0241 (0.0013, 0.0636)**	0.0181 (-0.0030, 0.0563)	**0.0060 (0.0019, 0.0120)**	**24.8070 (6.8636, 136.6087)**
PFHxA	0.0050 (-0.0071, 0.0277)	0.0027 (-0.0088, 0.0242)	**0.0023 (0.0002, 0.0051)**	45.9460 (-320.7506, 335.7951)
PFNA	**0.0855 (0.0461, 0.1289)**	**0.0687 (0.0296, 0.1123)**	**0.0167 (0.0082, 0.0267)**	**19.5881 (8.7335, 38.2253)**
PFDA	**0.0824 (0.0482, 0.1230)**	**0.0668 (0.0339, 0.1072)**	**0.0155 (0.0071, 0.0256)**	**18.8584 (7.7714, 35.6052)**
PFUnDA	**0.0617 (0.0299, 0.1033)**	**0.0467 (0.0158, 0.0889)**	**0.0150 (0.0079, 0.0235)**	**24.3429 (11.3651, 49.7779)**
PFDoDA	**0.0544 (0.0147, 0.1163)**	**0.0384 (0.0036, 0.0971)**	**0.0160 (0.0079, 0.0267)**	**29.3520 (13.3034, 81.9713)**
PFTrDA	**0.0404 (0.0095, 0.0894)**	0.0268 (-0.0016, 0.0722)	**0.0135 (0.0069, 0.0227)**	**33.5131 (13.2106, 114.8376)**
PFASa->TP->Sarcopenia				
Total PFOS	**0.0137 (0.0082, 0.0188)**	**0.0137 (0.0081, 0.0189)**	0.0000 (-0.0005, 0.0005)	0.2526 (-3.5366, 3.6898)
n-PFOS	**0.0186 (0.0129, 0.0230)**	**0.0186 (0.0128, 0.0230)**	0.0000 (-0.0005, 0.0006)	0.2259 (-2.6254, 3.3424)
Br-PFOS	**0.0429 (0.0327, 0.0522)**	**0.0429 (0.0327, 0.0520)**	0.0001 (-0.0005, 0.0007)	0.1932 (-1.1225, 1.8552)
1m-PFOS	**0.0565 (0.0199, 0.1098)**	**0.0560 (0.0197, 0.1103)**	0.0004 (-0.0017, 0.0027)	0.7820 (-3.6107, 6.0194)
iso-PFOS	**0.0663 (0.0366, 0.1023)**	**0.0660 (0.0349, 0.1005)**	0.0003 (-0.0020, 0.0028)	0.4571 (-3.2054, 4.7101)
3 + 4 + 5m-PFOS	**0.0425 (0.0233, 0.0676)**	**0.0425 (0.0234, 0.0676)**	-0.0000 (-0.0003, 0.0003)	-0.0100 (-0.6604, 0.5603)
∑m2-PFOS	**0.0760 (0.0229, 0.1403)**	**0.0753 (0.0208, 0.1403)**	0.0008 (-0.0029, 0.0048)	1.0040 (-4.4426, 8.4699)
PFHpS	**0.0774 (0.0248, 0.1362)**	**0.0768 (0.0242, 0.1361)**	0.0006 (-0.0023, 0.0037)	0.7442 (-3.4991, 5.7538)
Total PFHxS	**0.0330 (0.0027, 0.0700)**	**0.0325 (0.0016, 0.0691)**	0.0004 (-0.0011, 0.0023)	1.3642 (-5.6828, 15.7529)
n-PFHxS	**0.0302 (0.0052, 0.0701)**	**0.0298 (0.0045, 0.0695)**	0.0005 (-0.0012, 0.0022)	1.5365 (-5.0506, 14.2565)
Br-PFHxS	**0.0271 (0.0016, 0.0735)**	**0.0269 (0.0012, 0.0736)**	0.0002 (-0.0007, 0.0015)	0.7001 (-4.3455, 10.6987)
PFOA	**0.0230 (0.0136, 0.0296)**	**0.0230 (0.0134, 0.0294)**	0.0001 (-0.0002, 0.0006)	0.3123 (-1.0841, 2.4502)

PFHpA	**0.0270 (0.0011, 0.0758)**	**0.0266 (0.0010, 0.0757)**	0.0004 (-0.0009, 0.0021)	1.4313 (-7.2080, 15.0744)
PFHxA	0.0045 (-0.0076, 0.0280)	0.0043 (-0.0078, 0.0279)	0.0001 (-0.0004, 0.0008)	2.8469 (-33.6116, 49.4905)
PFNA	**0.0896 (0.0520, 0.1323)**	**0.0892 (0.0507, 0.1320)**	0.0004 (-0.0021, 0.0031)	0.4098 (-2.5009, 3.5662)
PFDA	**0.0835 (0.0504, 0.1279)**	**0.0832 (0.0502, 0.1272)**	0.0003 (-0.0016, 0.0025)	0.3771 (-1.8807, 3.2345)
PFUnDA	**0.0633 (0.0305, 0.1072)**	**0.0630 (0.0297, 0.1082)**	0.0003 (-0.0012, 0.0018)	0.4766 (-2.1218, 3.3591)
PFDoDA	**0.0577 (0.0148, 0.1202)**	**0.0574 (0.0147, 0.1206)**	0.0002 (-0.0010, 0.0019)	0.4077 (-2.3303, 3.8585)
PFTrDA	**0.0451 (0.0100, 0.0965)**	**0.0449 (0.0099, 0.0960)**	0.0003 (-0.0010, 0.0019)	0.5610 (-2.5946, 5.4190)
PFASa->ALT->Sarcopenia				
Total PFOS	**0.0137 (0.0085, 0.0190)**	**0.0137 (0.0085, 0.0191)**	-0.0000 (-0.0009, 0.0004)	-0.2057 (-5.7296, 2.6763)
n-PFOS	**0.0186 (0.0124, 0.0229)**	**0.0186 (0.0124, 0.0233)**	-0.0000 (-0.0009, 0.0004)	-0.0624 (-4.7327, 2.0129)
Br-PFOS	**0.0430 (0.0319, 0.0517)**	**0.0431 (0.0326, 0.0522)**	-0.0001 (-0.0028, 0.0011)	-0.3248 (-6.4132, 2.6706)
1m-PFOS	**0.0566 (0.0203, 0.1066)**	**0.0564 (0.0205, 0.1095)**	0.0002 (-0.0061, 0.0036)	0.3022 (-14.6375, 7.7586)
iso-PFOS	**0.0663 (0.0355, 0.0992)**	**0.0662 (0.0354, 0.1000)**	0.0001 (-0.0043, 0.0025)	0.1592 (-6.8188, 4.3421)
3 + 4 + 5m-PFOS	**0.0430 (0.0244, 0.0674)**	**0.0430 (0.0245, 0.0675)**	-0.0000 (-0.0008, 0.0004)	-0.0455 (-2.1178, 1.2164)
∑m2-PFOS	**0.0762 (0.0213, 0.1369)**	**0.0759 (0.0218, 0.1379)**	0.0003 (-0.0075, 0.0045)	0.3818 (-11.7085, 7.5608)
PFHpS	**0.0776 (0.0301, 0.1353)**	**0.0774 (0.0314, 0.1367)**	0.0003 (-0.0067, 0.0046)	0.3391 (-10.4865, 6.0122)
Total PFHxS	**0.0332 (0.0058, 0.0740)**	**0.0326 (0.0057, 0.0738)**	0.0005 (-0.0041, 0.0037)	1.5936 (-21.7993, 15.1844)
n-PFHxS	**0.0305 (0.0033, 0.0726)**	**0.0299 (0.0032, 0.0714)**	0.0005 (-0.0034, 0.0038)	1.7542 (-24.5075, 19.4653)
Br-PFHxS	**0.0267 (0.0020, 0.0716)**	**0.0263 (0.0020, 0.0717)**	0.0003 (-0.0021, 0.0022)	1.2787 (-15.4965, 13.8090)
PFOA	**0.0230 (0.0140, 0.0296)**	**0.0229 (0.0141, 0.0300)**	0.0001 (-0.0014, 0.0009)	0.3667 (-5.7437, 3.6343)
PFHpA	**0.0271 (0.0010, 0.0726)**	**0.0268 (0.0012, 0.0731)**	0.0003 (-0.0033, 0.0025)	1.0453 (-20.3314, 19.3431)
PFHxA	0.0046 (-0.0075, 0.0293)	0.0046 (-0.0074, 0.0293)	0.0000 (-0.0006, 0.0006)	0.6653 (-21.9679, 38.5418)
PFNA	**0.0897 (0.0548, 0.1311)**	**0.0895 (0.0541, 0.1315)**	0.0002 (-0.0053, 0.0027)	0.2689 (-6.1918, 2.9411)
PFDA	**0.0832 (0.0497, 0.1296)**	**0.0831 (0.0495, 0.1300)**	0.0001 (-0.0020, 0.0011)	0.1063 (-2.4302, 1.4269)
PFUnDA	**0.0632 (0.0298, 0.1076)**	**0.0631 (0.0299, 0.1080)**	0.0001 (-0.0015, 0.0008)	0.0818 (-2.4540, 1.4453)
PFDoDA	**0.0576 (0.0150, 0.1230)**	**0.0575 (0.0155, 0.1232)**	0.0001 (-0.0019, 0.0014)	0.1830 (-3.4567, 2.7476)
PFTrDA	**0.0456 (0.0128, 0.0982)**	**0.0454 (0.0132, 0.0986)**	0.0002 (-0.0020, 0.0014)	0.4687 (-4.8197, 3.8901)
PFASa->AST->Sarcopenia				

Total PFOS	**0.0138 (0.0082, 0.0191)**	**0.0131 (0.0078, 0.0178)**	**0.0007 (0.0000, 0.0020)**	**5.1764 (0.1834, 11.8264)**
n-PFOS	**0.0188 (0.0127, 0.0232)**	**0.0179 (0.0121, 0.0222)**	**0.0009 (0.0000, 0.0022)**	**4.6443 (0.2553, 10.9004)**
Br-PFOS	**0.0425 (0.0319, 0.0511)**	**0.0407 (0.0294, 0.0494)**	**0.0018 (0.0002, 0.0039)**	**4.1346 (0.4806, 9.4928)**
1m-PFOS	**0.0545 (0.0187, 0.1106)**	**0.0487 (0.0137, 0.1044)**	**0.0058 (0.0007, 0.0117)**	**10.6214 (1.1043, 29.1065)**
iso-PFOS	**0.0653 (0.0322, 0.0978)**	**0.0605 (0.0289, 0.0920)**	**0.0048 (0.0007, 0.0098)**	**7.3168 (0.9854, 17.5210)**
3 + 4 + 5m-PFOS	**0.0407 (0.0237, 0.0670)**	**0.0399 (0.0231, 0.0664)**	**0.0008 (0.0001, 0.0019)**	**2.0557 (0.1894, 5.6124)**
∑m2-PFOS	**0.0727 (0.0213, 0.1321)**	**0.0627 (0.0122, 0.1215)**	**0.0099 (0.0021, 0.0197)**	**13.6417 (2.7416, 42.7776)**
PFHpS	**0.0750 (0.0268, 0.1412)**	**0.0679 (0.0205, 0.1305)**	**0.0071 (0.0009, 0.0145)**	**9.4633 (1.6652, 27.0042)**
Total PFHxS	**0.0317 (0.0028, 0.0700)**	0.0258 (-0.0025, 0.0652)	**0.0058 (0.0013, 0.0116)**	**18.4741 (1.5653, 118.1820)**
n-PFHxS	**0.0289 (0.0023, 0.0679)**	0.0231 (-0.0029, 0.0622)	**0.0058 (0.0013, 0.0112)**	**19.9925 (1.3210, 90.3264)**
Br-PFHxS	**0.0267 (0.0006, 0.0778)**	0.0216 (-0.0031, 0.0703)	**0.0051 (0.0012, 0.0109)**	**18.9503 (1.6256, 115.9786)**
PFOA	**0.0233 (0.0139, 0.0296)**	**0.0221 (0.0134, 0.0280)**	**0.0011 (0.0001, 0.0029)**	**4.8264 (0.5990, 11.2403)**
PFHpA	**0.0261 (0.0019, 0.0733)**	0.0218 (-0.0007, 0.0675)	**0.0043 (0.0010, 0.0083)**	**16.3428 (2.4762, 98.9978)**
PFHxA	0.0040 (-0.0072, 0.0262)	0.0023 (-0.0082, 0.0245)	**0.0016 (0.0002, 0.0043)**	41.2468 (-347.9799, 302.9363)
PFNA	**0.0860 (0.0522, 0.1284)**	**0.0810 (0.0477, 0.1231)**	**0.0049 (0.0002, 0.0104)**	**5.7361 (0.2756, 13.0655)**
PFDA	**0.0813 (0.0468, 0.1213)**	**0.0777 (0.0438, 0.1173)**	**0.0036 (0.0003, 0.0077)**	**4.4356 (0.4521, 10.5364)**
PFUnDA	**0.0597 (0.0301, 0.1051)**	**0.0564 (0.0267, 0.1026)**	**0.0032 (0.0004, 0.0071)**	**5.4378 (0.6602, 14.3670)**
PFDoDA	**0.0547 (0.0152, 0.1167)**	**0.0505 (0.0126, 0.1121)**	**0.0042 (0.0008, 0.0086)**	**7.7074 (1.4050, 23.4292)**
PFTrDA	**0.0424 (0.0122, 0.0881)**	**0.0392 (0.0094, 0.0834)**	**0.0032 (0.0004, 0.0073)**	**7.5992 (1.1970, 27.4044)**
PFASa->GGT->Sarcopenia				
Total PFOS	**0.0138 (0.0079, 0.0190)**	**0.0133 (0.0076, 0.0179)**	0.0006 (-0.0000, 0.0013)	4.2056 (-0.1869, 8.1588)
n-PFOS	**0.0187 (0.0128, 0.0232)**	**0.0180 (0.0125, 0.0222)**	0.0007 (-0.0000, 0.0016)	3.7378 (-0.0201, 7.9762)
Br-PFOS	**0.0425 (0.0313, 0.0512)**	**0.0409 (0.0296, 0.0500)**	0.0015 (-0.0000, 0.0033)	3.6321 (-0.0617, 8.2634)
1m-PFOS	**0.0550 (0.0186, 0.1025)**	**0.0505 (0.0147, 0.0996)**	**0.0044 (0.0006, 0.0083)**	**8.0361 (1.4072, 23.7760)**
iso-PFOS	**0.0655 (0.0342, 0.1016)**	**0.0614 (0.0311, 0.0969)**	**0.0042 (0.0003, 0.0075)**	**6.3359 (0.4040, 13.6474)**
3 + 4 + 5m-PFOS	**0.0411 (0.0233, 0.0646)**	**0.0403 (0.0225, 0.0640)**	**0.0008 (0.0000, 0.0017)**	**1.9237 (0.0123, 5.2014)**
∑m2-PFOS	**0.0755 (0.0225, 0.1369)**	**0.0677 (0.0172, 0.1285)**	**0.0078 (0.0009, 0.0143)**	**10.2827 (0.8927, 26.5185)**
PFHpS	**0.0754 (0.0257, 0.1411)**	**0.0693 (0.0209, 0.1329)**	**0.0060 (0.0004, 0.0111)**	**8.0198 (0.4700, 20.0869)**

Total PFHxS	**0.0325 (0.0067, 0.0708)**	**0.0281 (0.0028, 0.0647)**	**0.0044 (0.0011, 0.0081)**	**13.4619 (2.3728, 57.8139)**
n-PFHxS	**0.0298 (0.0035, 0.0687)**	0.0254 (-0.0005, 0.0638)	**0.0043 (0.0013, 0.0083)**	**14.5761 (2.4724, 71.9110)**
Br-PFHxS	**0.0264 (-0.0005, 0.0716)**	0.0234 (-0.0022, 0.0679)	**0.0030 (0.0005, 0.0066)**	11.3790 (-19.1597, 61.0925)
PFOA	**0.0230 (0.0137, 0.0292)**	**0.0221 (0.0133, 0.0278)**	**0.0009 (0.0001, 0.0019)**	**3.7549 (0.3561, 7.2588)**
PFHpA	**0.0268 (0.0011, 0.0707)**	0.0230 (-0.0016, 0.0661)	**0.0038 (0.0006, 0.0067)**	14.2436 (-2.5225, 83.1005)
PFHxA	0.0049 (-0.0072, 0.0287)	0.0047 (-0.0073, 0.0277)	0.0003 (-0.0011, 0.0017)	5.2416 (-71.4055, 89.6927)
PFNA	**0.0885 (0.0522, 0.1335)**	**0.0833 (0.0468, 0.1284)**	0.0053 (-0.0002, 0.0095)	5.9392 (-0.1828, 12.4433)
PFDA	**0.0815 (0.0506, 0.1283)**	**0.0785 (0.0473, 0.1243)**	**0.0030 (0.0002, 0.0061)**	**3.7392 (0.2594, 8.2398)**
PFUnDA	**0.0611 (0.0308, 0.1075)**	**0.0584 (0.0271, 0.1062)**	**0.0027 (0.0001, 0.0055)**	**4.3501 (0.1537, 12.3409)**
PFDoDA	**0.0537 (0.0145, 0.1184)**	**0.0512 (0.0121, 0.1154)**	0.0025 (-0.0001, 0.0058)	4.6833 (-0.1416, 17.8571)
PFTrDA	**0.0426 (0.0114, 0.0918)**	**0.0404 (0.0094, 0.0899)**	0.0022 (-0.0001, 0.0056)	5.2138 (-0.4619, 17.8895)

Abbreviations: ALB, albumin; GLB, globin; TP, total protein; ALT, Alanine aminotransferase; AST, Aspartate Aminotransferase; GGT, Gamma-Glutamyl Transferase.

Model was adjusted for age, sex, education, alcohol drinking, smoking, family income, exercise, BMI, district.

Bolding indicates that associations were statistically significant ($P < 0{\cdot}05$).

[a] The variables were natural-log transformed.

[b] The complete mediation.

Table S13. Odds ratios (ORs) and 95% confidence intervals (CIs) for association between sarcopenia and PFAS in serum by sex(N=1,261).

PFAS[a](ng/mL)	Men	Women	*P-difference*
Total PFOS	**2.21 (1.48, 3.40)**	**2.90 (1.87, 4.59)**	0.364
n-PFOS	**2.06 (1.40, 3.11)**	**2.71 (1.80, 4.18)**	0.340
Br-PFOS	**2.01 (1.44, 2.90)**	**2.65 (1.67, 4.32)**	0.360
1m-PFOS	1.06 (0.75, 1.57)	**1.88 (1.28, 2.82)**	**0.022**
iso-PFOS	**1.69 (1.16, 2.50)**	**1.82 (1.26, 2.67)**	0.783
3 + 4 + 5m-PFOS	**1.55 (1.27, 1.99)**	**2.98 (1.79, 5.14)**	**0.014**
∑m2-PFOS	1.34 (0.92, 1.96)	**1.44 (1.01, 2.06)**	0.771
PFHpS	1.31 (0.88, 1.98)	**1.90 (1.23, 3.00)**	0.213
Total PFHxS	0.92 (0.64, 1.38)	**1.69 (1.06, 2.74)**	**0.024**
n-PFHxS	0.90 (0.63, 1.34)	**1.62 (1.03, 2.60)**	**0.029**
Br-PFHxS	1.12 (0.85, 1.51)	1.34 (1.00, 1.81)	0.374
PFOA	**2.51 (1.42, 4.61)**	**3.48 (1.77, 7.08)**	0.477
PFHpA	**1.28 (1.01, 1.66)**	1.21 (0.90, 1.66)	0.755
PFHxA	1.05 (0.83, 1.33)	1.07 (0.86, 1.34)	0.907
PFNA	**2.15 (1.29, 3.68)**	**2.56 (1.55, 4.33)**	0.626
PFDA	**1.99 (1.30, 3.11)**	**2.44 (1.63, 3.78)**	0.487
PFUnDA	**1.60 (1.11, 2.36)**	**2.24 (1.49, 3.48)**	0.247
PFDoDA	**1.37 (1.03, 1.87)**	**1.92 (1.34, 2.84)**	0.189
PFTrDA	**1.44 (1.01, 2.09)**	**1.77 (1.21, 2.68)**	0.424

Model was adjusted for sex, education, alcohol drinking, smoking, family income, exercise, BMI, district.

Bolding indicates statistically significant ($P < 0{\cdot}05$).

[a] The variables were natural-log transformed.

Table S14. Odds ratios (ORs) and 95% confidence intervals (CIs) for association between sarcopenia and PFAS in serum by age (N=1261).

PFAS[a] (ng/mL)	Younger[c] (N=891)	Older[b] (N=370)	$P_{\text{-}difference}$
Total PFOS	**2.79 (1.99, 3.99)**	**3.19 (1.89, 5.63)**	0.681
n-PFOS	**2.59 (1.87, 3.67)**	**2.81 (1.74, 4.75)**	0.791
Br-PFOS	**2.56 (1.89, 3.57)**	**2.76 (1.65, 4.85)**	0.818
1m-PFOS	**2.03 (1.48, 2.85)**	1.41 (0.91, 2.26)	0.198
iso-PFOS	**2.11 (1.57, 2.88)**	**2.04 (1.29, 3.31)**	0.912
3 + 4 + 5m-PFOS	**1.85 (1.46, 2.46)**	**2.67 (1.60, 4.71)**	0.231
∑m2-PFOS	**1.78 (1.33, 2.38)**	**1.70 (1.09, 2.72)**	0.882
PFHpS	**2.07 (1.48, 2.96)**	**1.75 (1.06, 3.03)**	0.604
Total PFHxS	**1.85 (1.28, 2.75)**	1.23 (0.76, 2.07)	0.205
n-PFHxS	**1.81 (1.25, 2.66)**	1.17 (0.74, 1.93)	0.161
Br-PFHxS	1.29 (0.99, 1.72)	1.23 (0.92, 1.70)	0.826
PFOA	**4.15 (2.49, 7.13)**	2.10 (0.99, 4.70)	0.157
PFHpA	**1.52 (1.20, 1.97)**	1.24 (0.94, 1.69)	0.305
PFHxA	1.10 (0.90, 1.35)	0.90 (0.70, 1.16)	0.232
PFNA	**2.96 (1.95, 4.55)**	**2.73 (1.51, 5.19)**	0.832
PFDA	**2.65 (1.85, 3.86)**	**2.40 (1.51, 3.99)**	0.757
PFUnDA	**2.15 (1.54, 3.08)**	**1.90 (1.23, 3.05)**	0.667
PFDoDA	**1.91 (1.43, 2.62)**	1.42 (1.00, 2.08)	0.218
PFTrDA	**1.79 (1.30, 2.52)**	1.47 (0.98, 2.31)	0.474

Adjusted for age, sex, education, alcohol drinking, smoking, family income, exercise, BMI, district.

Bolding odds ratios indicate that associations were statistically significant ($P < 0.05$).

a The PFAS concentrations were natural-log transformed.

b younger (< 65); older (≥ 65).

Table S15. Odds ratios (ORs) and 95% confidence intervals (CIs) for association between sarcopenia and PFAS in serum excluding 206 alcohol drinkers (N=1,055).

PFAS[a] (ng/mL)	**OR (95% CI)**	***P***
Total PFOS	**2.43 (1.74, 3.43)**	< 0.001
n-PFOS	**2.25 (1.65, 3.11)**	< 0.001
Br-PFOS	**2.13 (1.56, 2.96)**	< 0.001
1m-PFOS	**1.54 (1.15, 2.09)**	0.005
iso-PFOS	**1.65 (1.23, 2.22)**	0.001
3 + 4 + 5m-PFOS	**1.62 (1.30, 2.15)**	0.000
∑m2-PFOS	1.32 (0.99, 1.75)	0.056
PFHpS	**1.60 (1.16, 2.26)**	0.006
Total PFHxS	1.31 (0.95, 1.85)	0.110
n-PFHxS	1.28 (0.93, 1.78)	0.142
Br-PFHxS	1.22 (0.97, 1.54)	0.089
PFOA	**2.80 (1.74, 4.59)**	< 0.001
PFHpA	1.18 (0.96, 1.48)	0.122
PFHxA	1.07 (0.91, 1.28)	0.417
PFNA	**2.28 (1.55, 3.40)**	< 0.001
PFDA	**2.09 (1.53, 2.90)**	< 0.001
PFUnDA	**1.72 (1.29, 2.33)**	< 0.001
PFDoDA	**1.50 (1.17, 1.95)**	0.002
PFTrDA	**1.44 (1.11, 1.91)**	0.009

The crude model did not adjust for any covariates. Adjusted model was adjusted for age, sex, education, smoking, family income, exercise, BMI, district.

Bolding indicates that associations were statistically significant ($P < 0{\cdot}05$).

[a] The PFAS concentrations were natural-log transformed.

Table S16. Odds ratios (ORs) and 95% confidence intervals (CIs) for association between sarcopenia and PFAS in serum excluding 270 smokers (N=991).

PFAS[a] (ng/mL)	**OR (95% CI)**	***P***
Total PFOS	**2.53 (1.78, 3.63)**	< 0.001
n-PFOS	**2.33 (1.68, 3.27)**	< 0.001
Br-PFOS	**2.26 (1.62, 3.23)**	< 0.001
1m-PFOS	**1.75 (1.27, 2.43)**	0.001
iso-PFOS	**1.76 (1.29, 2.41)**	< 0.001
3 + 4 + 5m-PFOS	**1.68 (1.30, 2.32)**	0.001
∑m2-PFOS	**1.38 (1.03, 1.85)**	0.031
PFHpS	**1.67 (1.18, 2.40)**	0.005
Total PFHxS	**1.44 (1.01, 2.10)**	0.048
n-PFHxS	1.41 (1.00, 2.03)	0.059
Br-PFHxS	1.18 (0.94, 1.50)	0.173
PFOA	**2.97 (1.81, 5.01)**	< 0.001
PFHpA	1.15 (0.92, 1.46)	0.223
PFHxA	1.08 (0.91, 1.31)	0.381
PFNA	**2.35 (1.57, 3.57)**	< 0.001
PFDA	**2.12 (1.53, 2.99)**	< 0.001
PFUnDA	**1.84 (1.35, 2.56)**	< 0.001
PFDoDA	**1.49 (1.15, 1.97)**	0.003
PFTrDA	**1.50 (1.13, 2.03)**	0.007

The crude model did not adjust for any covariates. Adjusted model was adjusted for age, sex, education, drinking, family income, exercise, BMI, district.
Bolding indicates that associations were statistically significant ($P < 0·05$).
[a] The PFAS concentrations were natural-log transformed.

Table S17. Odds ratios (ORs) and 95% confidence intervals (CIs) for association between sarcopenia and PFAS in serum excluding 118 participants with diabetes (N=1,143).

PFAS[a] (ng/mL)	**OR (95% CI)**	***P***
Total PFOS	**2.29 (1.71, 3.11)**	< 0.001
n-PFOS	**2.15 (1.63, 2.87)**	< 0.001
Br-PFOS	**2.08 (1.59, 2.76)**	< 0.001
1m-PFOS	**1.42 (1.08, 1.88)**	0.014
iso-PFOS	**1.68 (1.29, 2.19)**	< 0.001
3 + 4 + 5m-PFOS	**1.67 (1.36, 2.13)**	< 0.001
∑m2-PFOS	**1.43 (1.10, 1.87)**	0.007
PFHpS	**1.52 (1.13, 2.06)**	0.006
Total PFHxS	1.21 (0.89, 1.67)	0.229
n-PFHxS	1.17 (0.87, 1.60)	0.313
Br-PFHxS	**1.24 (1.01, 1.54)**	0.041
PFOA	**2.82 (1.81, 4.47)**	< 0.001
PFHpA	**1.24 (1.03, 1.50)**	0.029
PFHxA	1.03 (0.88, 1.21)	0.708
PFNA	**2.18 (1.52, 3.14)**	< 0.001
PFDA	**2.03 (1.51, 2.76)**	< 0.001
PFUnDA	**1.73 (1.31, 2.30)**	< 0.001
PFDoDA	**1.47 (1.17, 1.86)**	0.001
PFTrDA	**1.50 (1.16, 1.96)**	0.003

The crude model did not adjust for any covariates. Adjusted model was adjusted for age, sex, education, alcohol drinking, smoking, family income, exercise, BMI, district.
Bolding indicates that associations were statistically significant ($P < 0{\cdot}05$).
[a] The PFAS concentrations were natural-log transformed.

Table S18. Odds ratios (ORs) and 95% confidence intervals (CIs) for association between sarcopenia and PFAS in serum excluding 217 participants with osteoporosis (N=1,044).

PFAS[a] (ng/mL)	**OR (95% CI)**	***P***
Total PFOS	**2.54 (1.82, 3.60)**	< 0.001
n-PFOS	**2.39 (1.74, 3.34)**	< 0.001
Br-PFOS	**2.17 (1.62, 2.96)**	< 0.001
1m-PFOS	**1.45 (1.08, 2.00)**	0.017
iso-PFOS	**1.82 (1.35, 2.47)**	< 0.001
3 + 4 + 5m-PFOS	**1.64 (1.34, 2.11)**	< 0.001
∑m2-PFOS	**1.41 (1.05, 1.88)**	0.020
PFHpS	**1.66 (1.20, 2.32)**	0.003
Total PFHxS	1.23 (0.89, 1.74)	0.223
n-PFHxS	1.20 (0.88, 1.68)	0.265
Br-PFHxS	1.18 (0.94, 1.50)	0.176
PFOA	**3.41 (2.09, 5.69)**	< 0.001
PFHpA	**1.32 (1.08, 1.64)**	0.010
PFHxA	1.13 (0.94, 1.36)	0.192
PFNA	**2.71 (1.79, 4.16)**	< 0.001
PFDA	**2.41 (1.70, 3.46)**	< 0.001
PFUnDA	**1.98 (1.44, 2.77)**	< 0.001
PFDoDA	**1.69 (1.29, 2.25)**	< 0.001
PFTrDA	**1.69 (1.26, 2.32)**	0.001

The crude model did not adjust for any covariates. Adjusted model was adjusted for age, sex, education, alcohol drinking, smoking, family income, exercise, BMI, district.

Bolding indicates that associations were statistically significant ($P < 0·05$).

[a] The PFAS concentrations were natural-log transformed.

Table S19. Odds ratios (ORs) and 95% confidence intervals (CIs) for association between sarcopenia and PFAS in serum excluding 64 participants with malnutrition (N=1,197).

PFAS[a] (ng/mL)	**OR (95% CI)**	***P***
Total PFOS	**2.72 (2.00, 3.76)**	< 0.001
n-PFOS	**2.56 (1.91, 3.48)**	< 0.001
Br-PFOS	**2.29 (1.72, 3.10)**	< 0.001
1m-PFOS	**1.55 (1.17, 2.07)**	0.003
iso-PFOS	**1.95 (1.48, 2.57)**	< 0.001
3 + 4 + 5m-PFOS	**1.87 (1.46, 2.48)**	< 0.001
∑m2-PFOS	**1.51 (1.16, 1.97)**	0.002
PFHpS	**1.77 (1.30, 2.44)**	< 0.001
Total PFHxS	1.36 (0.99, 1.90)	0.062
n-PFHxS	1.32 (0.97, 1.83)	0.083
Br-PFHxS	**1.27 (1.02, 1.61)**	0.039
PFOA	**3.15 (1.98, 5.11)**	< 0.001
PFHpA	**1.34 (1.09, 1.65)**	0.006
PFHxA	1.07 (0.90, 1.27)	0.446
PFNA	**2.77 (1.89, 4.10)**	< 0.001
PFDA	**2.56 (1.86, 3.57)**	< 0.001
PFUnDA	**2.11 (1.57, 2.87)**	< 0.001
PFDoDA	**1.71 (1.33, 2.24)**	< 0.001
PFTrDA	**1.81 (1.36, 2.44)**	< 0.001

The crude model did not adjust for any covariates. Adjusted model was adjusted for age, sex, education, alcohol drinking, smoking, family income, exercise, BMI, district.

Bolding indicates that associations were statistically significant ($P < 0·05$).

[a] The PFAS concentrations were natural-log transformed.

Table S20. Odds ratios (ORs) and 95% confidence intervals (CIs) for association between sarcopenia and PFAS in serum excluding 35 participants with CHD and stroke (N=1,226).

PFAS[a] (ng/mL)	**OR (95% CI)**	***P***
Total PFOS	**2.61 (1.92, 3.59)**	< 0.001
n-PFOS	**2.43 (1.82, 3.29)**	< 0.001
Br-PFOS	**2.26 (1.70, 3.05)**	< 0.001
1m-PFOS	**1.64 (1.24, 2.20)**	0.001
iso-PFOS	**1.90 (1.45, 2.50)**	< 0.001
3 + 4 + 5m-PFOS	**1.72 (1.38, 2.22)**	< 0.001
∑m2-PFOS	**1.57 (1.20, 2.07)**	0.001
PFHpS	**1.78 (1.31, 2.45)**	< 0.001
Total PFHxS	**1.41 (1.03, 1.97)**	0.035
n-PFHxS	**1.37 (1.01, 1.90)**	0.047
Br-PFHxS	1.22 (0.99, 1.52)	0.066
PFOA	**3.26 (2.06, 5.26)**	< 0.001
PFHpA	**1.24 (1.02, 1.51)**	0.031
PFHxA	1.05 (0.89, 1.24)	0.561
PFNA	**2.50 (1.73, 3.65)**	< 0.001
PFDA	**2.22 (1.64, 3.04)**	< 0.001
PFUnDA	**1.82 (1.38, 2.42)**	< 0.001
PFDoDA	**1.47 (1.17, 1.88)**	0.001
PFTrDA	**1.47 (1.14, 1.92)**	0.004

The crude model did not adjust for any covariates. Adjusted model was adjusted for age, sex, education, alcohol drinking, smoking, family income, exercise, BMI, district.
Bolding indicates that associations were statistically significant ($P < 0·05$).
[a] The PFAS concentrations were natural-log transformed.

Table S21. Odds ratios (ORs) and 95% confidence intervals (CIs) for association between sarcopenia and PFAS in serum excluding 80 participants with dyslipidemia (N=1,181).

PFAS[a] (ng/mL)	**OR (95% CI)**	***P***
Total PFOS	**2.86 (2.03, 4.13)**	< 0.001
n-PFOS	**2.58 (1.87, 3.64)**	< 0.001
Br-PFOS	**2.58 (1.86, 3.67)**	< 0.001
1m-PFOS	**1.90 (1.36, 2.72)**	< 0.001
iso-PFOS	**2.14 (1.57, 2.95)**	< 0.001
3 + 4 + 5m-PFOS	**1.76 (1.39, 2.37)**	< 0.001
∑m2-PFOS	**1.87 (1.34, 2.62)**	< 0.001
PFHpS	**1.99 (1.39, 2.88)**	< 0.001
Total PFHxS	**1.48 (1.03, 2.18)**	0.039
n-PFHxS	1.43 (1.00, 2.07)	0.057
Br-PFHxS	**1.41 (1.05, 1.90)**	0.024
PFOA	**3.64 (2.20, 6.17)**	< 0.001
PFHpA	**1.67 (1.24, 2.27)**	0.001
PFHxA	1.05 (0.85, 1.30)	0.647
PFNA	**2.93 (1.91, 4.59)**	< 0.001
PFDA	**2.47 (1.73, 3.58)**	< 0.001
PFUnDA	**1.91 (1.39, 2.68)**	< 0.001
PFDoDA	**1.94 (1.40, 2.75)**	< 0.001
PFTrDA	**1.65 (1.22, 2.26)**	0.001

The crude model did not adjust for any covariates. Adjusted model was adjusted for age, sex, education, alcohol drinking, smoking, family income, exercise, BMI, district.

Bolding indicates that associations were statistically significant ($P < 0{\cdot}05$).

[a] The PFAS concentrations were natural-log transformed.

Table S22. Odds ratios (ORs) and 95% confidence intervals (CIs) for association between sarcopenia and PFAS in serum excluding participants with PFAS levels below the limit of quantitation (LOQ) (N = 735).

PFAS[a] (ng/mL)	**OR (95% CI)**	***P***
Total PFOS	**2.31 (1.61, 3.37)**	< 0.001
n-PFOS	**2.17 (1.54, 3.10)**	< 0.001
Br-PFOS	**2.17 (1.53, 3.14)**	< 0.001
1m-PFOS	1.37 (0.99, 1.92)	0.059
iso-PFOS	**1.62 (1.18, 2.22)**	0.003
3 + 4 + 5m-PFOS	**2.31 (1.65, 3.32)**	< 0.001
∑m2-PFOS	**1.39 (1.02, 1.89)**	0.034
PFHpS	**1.58 (1.08, 2.30)**	0.017
Total PFHxS	1.19 (0.81, 1.76)	0.376
n-PFHxS	1.14 (0.78, 1.66)	0.510
Br-PFHxS	1.34 (0.96, 1.87)	0.086
PFOA	**5.09 (2.88, 9.30)**	< 0.001
PFHpA	**1.72 (1.26, 2.37)**	0.001
PFHxA	1.00 (0.71, 1.40)	0.998
PFNA	**2.47 (1.57, 3.94)**	< 0.001
PFDA	**2.16 (1.47, 3.21)**	< 0.001
PFUnDA	**1.71 (1.20, 2.46)**	0.003
PFDoDA	**1.76 (1.22, 2.57)**	0.003
PFTrDA	**1.61 (1.11, 2.35)**	0.013

The crude model did not adjust for any covariates. Adjusted model was adjusted for age, sex, education, alcohol drinking, smoking, family income, exercise, BMI, district.
Bolding indicates that associations were statistically significant ($P < 0{\cdot}05$).
[a] The PFAS concentrations were natural-log transformed.

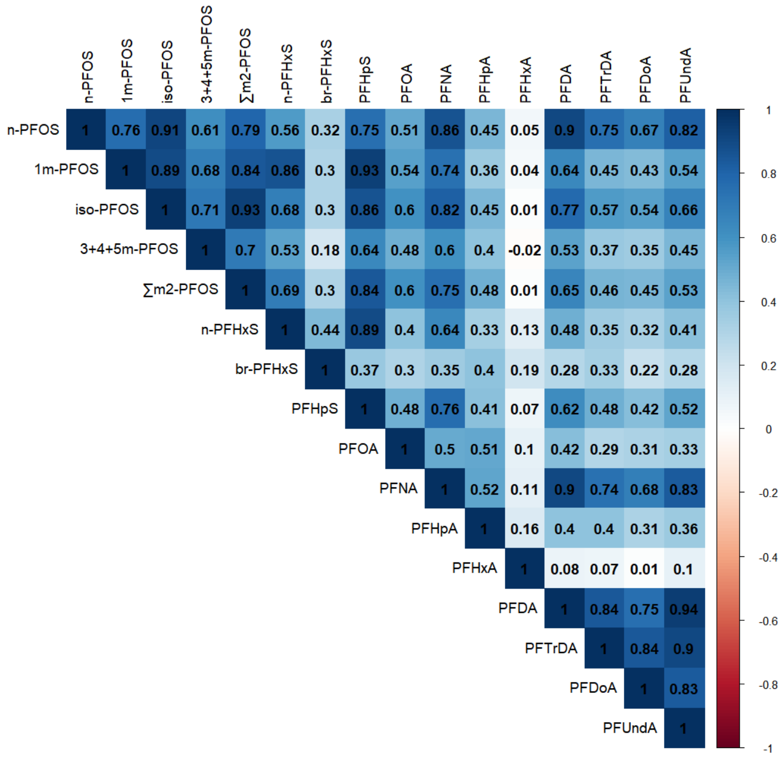

Figure S1 Spearman correlation coefficients among serum PFAS.a
